# Continuous UV irradiation played an inconclusive role in selecting the canonical genetic alphabet

**DOI:** 10.1073/pnas.2602433123

**Published:** 2026-09-28

**Authors:** Marcos Jusino-Maldonado, William C. Platten, Henry J. Wolfe, Zoe R. Todd

**Affiliations:** ^a^ Department of Chemistry, University of Wisconsin-Madison, Madison, WI 53706; ^b^ Department of Astronomy, University of Wisconsin-Madison, Madison, WI 53706

**Keywords:** origins of life, UV radiation, photochemistry, prebiotic chemistry

## Abstract

An abundance of heterocyclic molecules analogous to canonical nucleobases (i.e., adenine, thymine, guanine, cytosine, and uracil) used as the genetic alphabet in DNA and RNA could have been present in various prebiotic environments on early Earth. However, extant biology generally uses the canonical nucleobases and nucleosides as the genetic alphabet, and not any number of possible noncanonical counterparts. Ultraviolet (UV) irradiation was likely abundant on the surface of the early Earth and has been suggested to play a role in the selection of the canonical nucleobases and nucleosides as the genetic alphabet of life. Previous studies examining the photophysics of various canonical and noncanonical nucleobases and nucleosides have suggested that the canonical molecules have shorter excited-state lifetimes and may therefore be less prone to photodamage. Here, we evaluate the photostabilities of various canonical and noncanonical nucleobases and nucleosides under continuous UV irradiation at λ=254 nm to determine their rates of degradation and compare how photostable they are. Additionally, we estimate the relative photostabilities of these molecules under a plausible early Earth UV environment. We find that the canonical nucleobases and nucleosides span nearly the entire range of observed rates of photodegradation. These results indicate that the selective factors for the genetic alphabet may be more complex than purely due to selection by UV photostability.

## Significance

Ultraviolet (UV) light was likely to be present in abundance on the surface of the early Earth. UV light has been suggested to influence prebiotic chemistry, either through driving productive synthesis or degrading photounstable molecules, potentially driving the selection of the building blocks of life. Here, we investigate the photostability of potential nucleic acid building blocks of life under continuous UV irradiation. We find that the canonical molecules used by life today are not necessarily more photostable under continuous UV irradiation than various noncanonical analogues. This work suggests that the selection of life’s genetic alphabet was not purely driven by the photostability of molecular building blocks and other factors may have played a role.

Chemistry and physics precede life as we know it, and understanding their complex interplay may help shed light on the processes involved in the emergence and evolution of life. The ways by which life was abiotically set to begin on Earth still remains unresolved. One source of energy possibly available for driving prebiotic synthesis on the early Earth is ultraviolet (UV) light (1–4). Before the Great Oxidation Event (5), the Earth’s atmosphere lacked the UV shielding benefits that oxygen-containing molecules (e.g. O_2_ and O_3_) provide nowadays. Studies have suggested that, although the Sun was approximately 70% as luminous as its present-self during the time when prebiotic chemistry and origins of life are estimated to have occurred, it likely emitted a higher fractional output in the UV range, yielding an approximately similar photon flux from 200 to 300 nm as the modern Sun (6, 7). Therefore, it is likely that the surface of the Earth was exposed to greater UV irradiation early during the planet’s history due to the lack of O_2_ and O_3_ (8, 9), potentially influencing prebiotic chemistry occurring on the surficial bodies of water on the planet.

UV irradiation is capable of altering and damaging biomolecules, such as RNA and DNA, which may lead to detrimental consequences on life, including harmful mutations at a cellular level that may lead to cancer and cell death (10–12). UV irradiation of pyrimidine monomers is known to result in photohydration, where a water molecule adds to the C(5) and C(6) positions in the heterocyclic ring upon UV irradiation (13–15). Prebiotically relevant molecules like members of the 2-aminoazole family, which have been suggested to have played key roles in the synthesis and chemical activation of ribonucleotides, also suffer photodestruction upon UV irradiation (16). Additionally, vital biomolecules such as proteins and amino acids can also undergo photodamage by UV irradiation (17, 18). Another common way for UV light to damage DNA and RNA is by the production of cyclobutane pyrimidine dimers (CPDs), where two adjacent pyrimidine bases (e.g., cytosine–cytosine and thymine–thymine) in oligomers get interconnected by a four-membered ring structure (11). At the same time, some beneficial properties of UV irradiation have been demonstrated in studies regarding the emergence of life. UV radiation may provide an important abiotic mechanism for the synthesis of important building blocks of life, such as amino acids, lipids, sugars, and ribonucleotides (3, 4, 19–22). In some cases, photoexcitation of adjacent nucleobases in select DNA or RNA sequences can allow charge transfer of electrons to repair CPDs photolesions in the absence of repair enzymes (23, 24), reinforcing the idea that such UV fluxes may also have been beneficial for prebiotic chemistry.

DNA is composed of the Watson–Crick–Franklin bases adenine, thymine, guanine, and cytosine; in RNA, thymine is replaced with uracil. Most hypotheses for the origins of life advocate for RNA preceding DNA as genetic material as part of the so-called “RNA World” (25). The RNA World hypothesis posits that RNA preceded DNA based on its capacity to act both as an information-storing polymer and as a catalyst (26), potentially for its own formation (27–29). While this is not yet a solved problem, in vitro evolution experiments have demonstrated that an RNA polymerase ribozyme can catalyze the synthesis of its evolutionary ancestor (30, 31). For the purposes of this study, we focus on the nucleobase and nucleoside monomeric building blocks of RNA and DNA. These molecules comprise the genome of all life as we know on Earth, and are therefore known as canonical nucleobases and nucleosides. However, additional heterocyclic analogs exist, some of which are utilized by organisms in biological processes (32, 33). These compounds could have plausibly been synthesized alongside the canonical nucleobases on the primitive Earth or have been detected in meteorites (34, 35). These are known as noncanonical nucleobases and nucleosides, and they include common modifications to the canonical nucleobases and nucleosides which can be found in RNA (36, 37).

Fialho et al. (38) provided a thorough review on the prebiotic syntheses of various noncanonical nucleobases and nucleosides. All of the noncanonical nucleobases and nucleosides under the scope of this study have been invoked for prebiotic settings due to their synthetic pathways, plausible prebiotic sources, and/or known roles in extant biology. Noncanonical nucleobases like melamine and cyanuric acid were reported to be synthesized alongside the canonical nucleobases adenine, cytosine, and uracil in Miller–Urey experiments subjected to freeze–thaw cycles (39). Xanthine and hypoxanthine, the nucleobase forms of xanthosine and inosine respectively, along with 2,6-diaminopurine have been found in samples of multiple carbonaceous meteorites (e.g., Murchison) alongside canonical nucleobases (34, 35). Xanthine has been suggested to form under prebiotically relevant conditions via computational calculations (40), and solid state heating of xanthine with D-ribose has been shown to yield xanthosine (41). Hypoxanthine, the nucleobase of inosine, can be synthesized from HCN-driven chemistry (42) and via dehydration of glycinamide and N,N^′^-diformylurea (43). Inosine can be formed from hypoxanthine via reaction with carbohydrate phosphates (44) or solid state heating with D-ribose (41). Barbituric acid, cyanuric acid, and melamine can be synthesized under potentially prebiotic conditions, from cyanide as a feedstock molecule under various hydrothermal conditions (45). Cyanuric acid and melamine can also be synthesized from freeze–thaw cycles of urea-containing solutions where spark discharge was used (39). 2,4,6-triaminopyrimidine has been shown to form spontaneous glycosidic bonds with ribose, forming nucleoside structures at higher yields than the canonical nucleobases (46). Similarly, melamine and barbituric acid also react with ribose to form their respective nucleosides, with yields of 27% and 90%, respectively (47). Urazole has been invoked as a plausible component of a pre-RNA, as its prebiotic synthesis was likely, and potentially favorable (48). Zebularine, the nucleoside of 2-pyrimidinone (or 2-hydroxypyrimidine as the tautomeric form), has been synthesized under drying and heating conditions at yields which are higher than known adenine and hypoxanthine syntheses under similar conditions (49). 2-thiocytosine has been synthesized from chemical pathways starting with thiourea and cyanoacetaldehyde; subsequent hydrolysis can yield 2-thiouracil, cytosine, and uracil (50). The thiopyrimidine nucleosides and nucleotides can undergo photochemical processing to yield canonical nucleosides and nucleotides through photoanomerization (51).

UV light has been suggested to have played a role in the selection of the canonical genetic alphabet over the noncanonical genetic alphabet for encoding the genome of life on Earth (52, 53). Photophysical experiments have suggested differences in excited-state lifetimes between canonical and noncanonical nucleobases and nucleosides after UV irradiation, mainly driven by factors such as functional groups, the nature of the solvent, and the presence of conical intersections within canonical nucleobases (54). Beckstead et al. (52) showed that the excited states of canonical nucleobases and nucleosides produced by UV irradiation have significantly shorter lifetimes than those of noncanonical nucleobases and nucleosides. The presence of conical intersections allows them to quickly redistribute excess energy toward their surrounding environment via nonradiative decay, ultimately returning the molecule to its electronic ground state faster and thus potentially limiting photodegradation. Importantly, Beckstead et al. (52) primarily focus on modified canonical nucleobases and nucleosides as noncanonical molecules, rather than more exotic noncanonical nucleobases and nucleosides such as cyanuric acid, melamine, etc. In this study, we explore the photostability of canonical and noncanonical nucleobases and nucleosides under continuous UV irradiation in aqueous solutions and determine their rates of photodegradation to assess their lifetimes. Given the potential for UV light to be both productive and destructive to prebiotic chemistry, and the prevalence of UV fluxes on the surface of the early Earth, it is critical to understand the photostability of prebiotically relevant molecules to UV irradiation to determine whether UV light was a key factor in the selection of the extant genetic alphabet.

## Materials and Methods

We screened 24 commercially available canonical and noncanonical nucleobases and nucleosides at target concentrations of 50 μM (actual concentrations ranged from 30 to 68.8, see *SI Appendix*, Table S4) under UV irradiation in a Rayonet RPR-200 photochemical reactor with 254 nm mercury lamps. Solutions were prepared as described in *SI Appendix*, section I, including a degassing step to ensure minimal oxygen presence before irradiation. The irradiation geometry and determination of the photon flux via chemical actinometry, $(1.67\;\pm \;0.033)\times 10^{16}\frac{\text{photons}}{\text{s}\;{\text{cm}}^{\text{2}}}$, is described in *SI Appendix*, section VI. Periodically throughout the irradiation, samples were removed from the irradiation source and their UV–Vis spectra were measured in a Agilent Cary 60 spectrophotometer. Standard curves were constructed for each molecule (*SI Appendix*, section II) to convert absorbance at the peak wavelength to concentration. First-order kinetics plots of ln(concentration) vs. time were observed to be initially linear. Occasionally, nonlinear behavior was observed at later timepoints during the irradiation, in which case we used a criterion for where and when to truncate nonlinear data (described further in *SI Appendix*, section III). Rate constants were determined from the slopes of fits of ln(concentration) vs. time for all experiments. All molecules were tested in triplicate; *SI Appendix*, section III shows all spectra and kinetics plots. The average rate constant was calculated for each triplicate set. Errors in the rate constants were calculated as described in *SI Appendix*, section VIII. The pH was measured before and after irradiation to ensure significant drift did not occur (*SI Appendix*, section V).

## Results

**Fig. 1.**
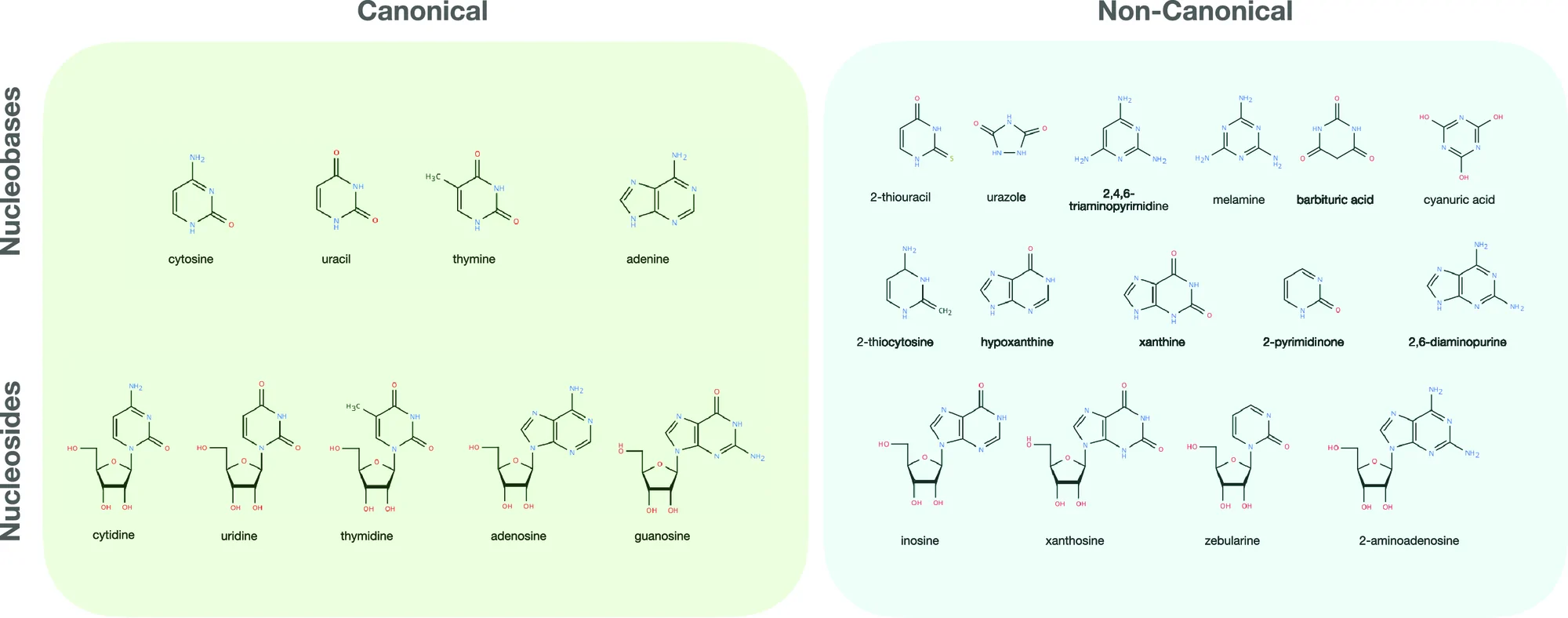
Categorization of heterocyclical molecules probed within this study. Green indicates the canonical nucleobases/sides and blue shows the noncanonical nucleobases/sides. Nucleobases (heterocyclic compound) are located at the top and nucleosides (heterocyclic compound + ribose) are located in the bottom row.

**Fig. 2.**
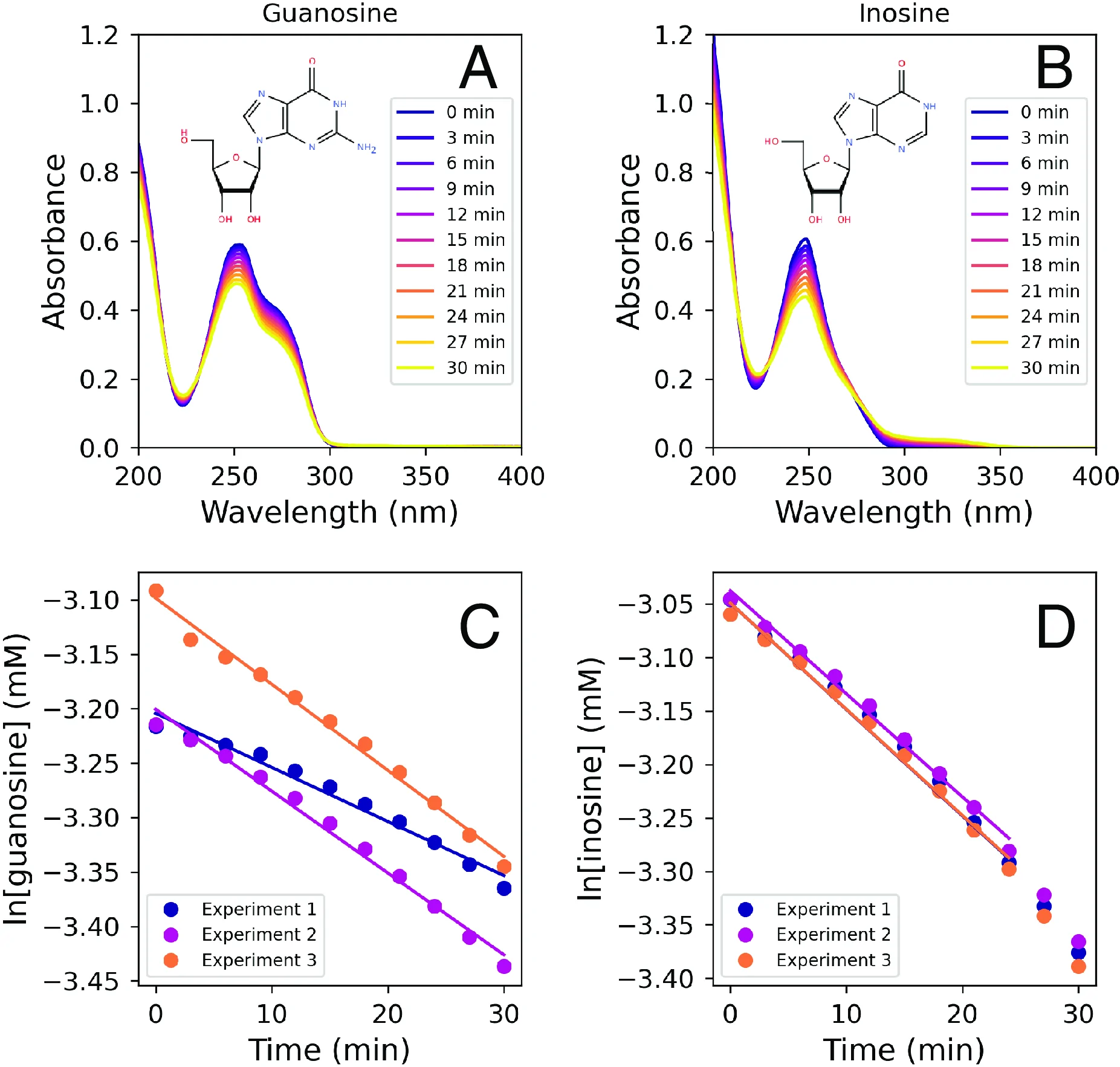
Example canonical and noncanonical purine nucleoside photodegradation through time at 254 nm continuous irradiation. (*A*) Guanosine (peak at $\lambda_{\max}=252$ nm) and (*B*) inosine (peak at $\lambda_{\max}=249$ nm) UV–Vis photodegradation spectra with irradiation time indicated by color. (*C*) Guanosine and (*D*) inosine first-order kinetics through irradiation time of triplicates. The value of the slope in the best-fit lines of (*C*) and (*D*) gives the experimentally measured values of the guanosine, k = $\left(6.80\;\pm \;1.10\right)\times 10^{-3}$ min^−1^, and inosine, k = $\left(9.84\;\pm \;0.87\right)\times 10^{-3}$ min^−1^, rate constants. Guanosine is ≈1.4 times more photostable than inosine.

We subjected various canonical and noncanonical nucleobases and nucleosides, including the canonical nucleobases (with the exception of guanine, due to solubility issues), the canonical nucleosides, and thirteen noncanonical nucleobases and nucleosides (see Table 1 and Fig. 1 for complete list) to continuous UV irradiation at λ=254 nm to determine their rate constants of photodegradation. Fig. 2 *A* and *B* show the absorbance spectra of guanosine and inosine throughout the course of the irradiation. The absorbance at the maximum wavelength (252 nm and 249 nm for guanosine and inosine, respectively), was used to calculate concentration at each timepoint. Plots of ln(concentration) vs. time are observed to be linear (Fig. 2 *C* and *D*), consistent with first-order kinetics. The negative slopes of the trend lines correspond to the rate constants of degradation, *k*. Guanosine, $k=\left(6.80\;\pm \;1.10\right)\;\times \;10^{-3}$ min^−1^ (as determined from the triplicate set), degrades at a slower rate than inosine, k = $\left(9.84\;\pm \;0.87\right)\;\times \;10^{-3}$ min^−1^ (again calculated from the triplicate set), as observed in the decrease of their respective concentrations through time.

**Table 1.** Rate constants and concentrations for each molecule, with associated SE from each triplicate irradiation

Molecule	$\lambda_{\max}$ (nm)	k (min^−1^)	Conc. (μM)
2,4,6-triamino-pyrimidine	270	$\left(5.73\;\pm \;1.02\right)\;\times \;10^{-2}$	48.6±0.33
2,6-diaminopurine	280	$\left(1.58\;\pm \;0.14\right)\;\times \;10^{-2}$	55.6±0.10
2-aminoadenosine	280	$\left(9.22\;\pm \;0.86\right)\;\times \;10^{-3}$	48.9±0.70
2-pyrimidinone	299	$\left(3.86\;\pm \;0.47\right)\;\times \;10^{-1}$	50.3±0.83
2-thiocytosine	268	$\left(8.95\;\pm \;0.81\right)\;\times \;10^{-2}$	37.0±0.37
2-thiouracil	274	$\left(4.41\;\pm \;0.41\right)\;\times \;10^{-2}$	46.9±0.30
Adenine	261	$\left(2.19\;\pm \;0.22\right)\;\times \;10^{-3}$	51.7±0.30
Adenosine	259	$\left(8.38\;\pm \;1.45\right)\;\times \;10^{-4}$	45.6±0.39
Barbituric acid	257	$\left(6.03\;\pm \;1.01\right)\;\times \;10^{-2}$	45.2±0.07
Cyanuric acid	213	$\left(8.07\;\pm \;1.16\right)\;\times \;10^{-3}$	59.1±4.0
Cytidine	271	$\left(2.18\;\pm \;0.21\right)\;\times \;10^{-1}$	40.0±0.26
Cytosine	271	$\left(3.76\;\pm \;0.36\right)\;\times \;10^{-2}$	32.8±0.51
Guanosine	252	$\left(6.80\;\pm \;1.10\right)\;\times \;10^{-3}$	41.9±1.8
Hypoxanthine	249	$\left(5.79\;\pm \;0.62\right)\;\times \;10^{-3}$	56.5±0.59
Inosine	249	$\left(9.84\;\pm \;0.87\right)\;\times \;10^{-3}$	47.4±0.22
Melamine	203	$\left(1.20\;\pm \;0.12\right)\;\times \;10^{-3}$	34.4±0.69
Thymidine	267	$\left(8.16\;\pm \;0.90\right)\;\times \;10^{-3}$	49.4±0.65
Thymine	264	$\left(9.76\;\pm \;1.07\right)\;\times \;10^{-3}$	31.1±0.59
Uracil	258	$\left(6.95\;\pm \;0.62\right)\;\times \;10^{-2}$	45.5±0.31
Urazole	200	$\left(6.83\;\pm \;0.95\right)\;\times \;10^{-3}$	59.6±1.4
Uridine	262	$\left(5.48\;\pm \;0.51\right)\;\times \;10^{-1}$	54.5±0.23
Xanthine	268	$\left(1.33\;\pm \;0.45\right)\;\times \;10^{-2}$	50.7±0.74
Xanthosine	261	$\left(1.83\;\pm \;0.19\right)\;\times \;10^{-2}$	33.8±0.27
Zebularine	304	$\left(5.55\;\pm \;0.50\right)\;\times \;10^{-2}$	68.1±0.41

**Fig. 3.**
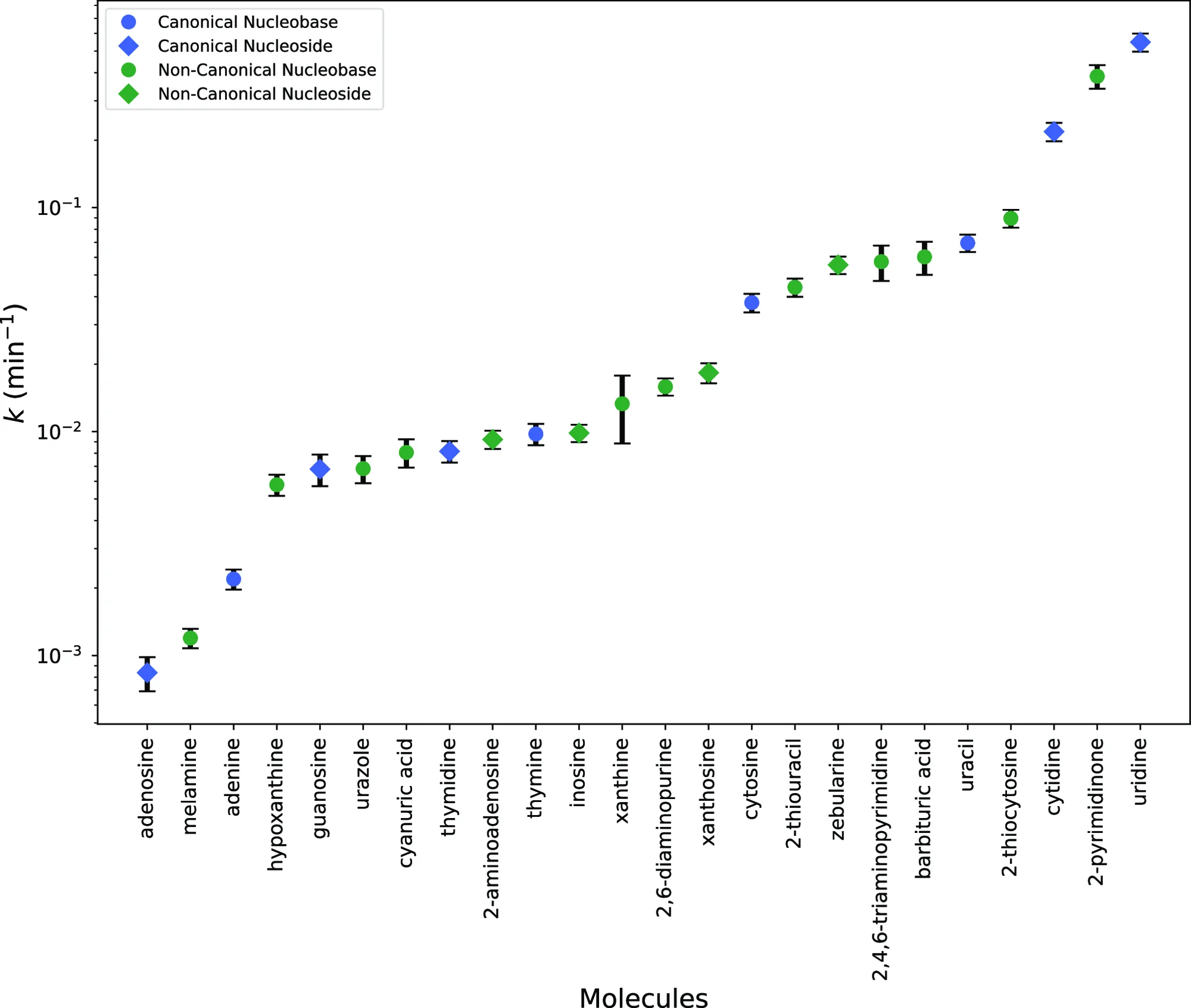
Canonical and noncanonical nucleobase and nucleoside rate constants *k*, ordered from most photostable (*Left*) to least photostable (*Right*). Blue indicates canonical molecules while green shows noncanonical molecules. Circles represent nucleobases and diamonds show nucleosides.

The same procedure was performed for a total of 24 molecules, including four canonical nucleobases (excluding guanine due to solubility issues), five canonical nucleosides, and 15 noncanonical nucleobases and nucleosides (Fig. 1). Two molecules, 5-methylcytosine and 5-methylcytidine, showed shifting absorption features upon irradiation, making it difficult to accurately quantify the rates of photodegradation (see *SI Appendix*, Figs. S49 and S50), buffered in 16 mM phosphate, pH ≈7.4 to mimic conditions in Hoehn et al. (55). Irradiation of each molecule was performed at least three times to create replicate sets, enabling the calculation of the average and SE for each rate constant. Fig. 3 shows the measured rate constants, arranged from smallest (most photostable molecules) to largest (least photostable molecules). The measured rate constants spanned several orders of magnitude: from ≈10^−4^ to $10^{-1}$ min^−1^, indicating a wide difference in the photostability of these molecules (Table 1). The two most photostable molecules were adenosine and melamine at $k=\left(8.38\;\pm \;1.45\right)\;\times \;10^{-4}$ min^−1^ and $k=\left(1.20\;\pm \;0.12\right)\;\times \;10^{-3}$ min^−1^, respectively. The two least photostable molecules were 2-pyrimidinone and uridine, with $k=\left(3.86\;\pm \;0.47\right)\;\times \;10^{-1}$ min^−1^ and $k=\left(5.48\;\pm \;0.51\right)\;\times \;10^{-1}$ min^−1^, respectively. Cytidine followed with a $k=\left(2.18\;\pm \;0.21\right)\;\times \;10^{-1}$ min^−1^ rate constant. The findings regarding cytidine and uridine are particularly of interest because, in a prebiotic setting with all canonical nucleobases and nucleosides present, cytidine and uridine would degrade at much faster rates than their other canonical counterparts. We observe that the canonical nucleobases and nucleosides fall across nearly the entire range of observed rate constants.

Comparing among only the canonical nucleosides, adenosine is the most photostable, followed by guanosine, thymidine, and finally cytidine and uridine. By calculating the rate constant ratios of the canonical nucleobases and nucleosides, we see that adenosine is ≈8.1 times more photoresistant than guanosine (from $k_{\mathrm{guanosine}}/k_{\mathrm{adenosine}}$), ≈9.7 times more than thymidine (from $k_{\mathrm{thymidine}}/k_{\mathrm{adenosine}}$), ≈261 times more than cytidine (from $k_{\mathrm{cytidine}}/k_{\mathrm{adenosine}}$), and ≈654 times more than uridine (from $k_{\mathrm{uridine}}/k_{\mathrm{adenosine}}$). We also determined the rate constants for photodegradation of the canonical nucleobases (adenine, cytosine, uracil, and thymine), excluding guanine due to solubility issues. Interestingly, when comparing the rate constants from the nucleobases cytosine and uracil to their nucleosides cytidine and uridine, we found that their nucleobase forms are more photo stable than their nucleoside counterparts. Cytosine was found to be ≈5.8 times more photoresistant than cytidine, and uracil ≈7.9 times more than uridine. In the case of A and T, differences in the photostabilities of the nucleobases and nucleosides were not as pronounced ($k_{\mathrm{adenosine}}/k_{\mathrm{adenine}}\approx 0.38$; $k_{\mathrm{thymidine}}/k_{\mathrm{thymine}}\approx 0.84$).

## Discussion

Cytidine and uridine are of interest due to their comparatively quick rates of photodegradation as compared to other canonical and noncanonical nucleobases and nucleosides, but also because of the observed difference in rates of degradation between their nucleobase and nucleoside forms. Upon UV irradiation, cytidine and uridine are known to form photohydrates, e.g., 6-hydroxy-5,6-dihydro-cytidine (14) and 6-hydroxy-5,6-dihydrouridine (13). Uridine can be recovered either through thermal dehydration or by addition of acid (56). The photohydrate of cytidine derivatives is unstable: it can either be dehydrated to recover cytidine or it can deaminate to form the uridine photohydrate (19, 57, 58). The production of these photohydrates and their respective intermediates can be tracked within its UV–Vis spectrum as the peak that increases around 240 nm with cytidine irradiation time (*SI Appendix*, Fig. S35), a phenomenon that was also observed by Todd et al. (58). The rates of photochemical conversion determined here likely correspond to the degradation to the photohydrated species for C and U. Since some amount of C and U can be recovered from the photohydrates upon heating under dark conditions, these rates may not be entirely indicative of the prebiotic availability of these molecules compared to the other nucleobases and nucleosides examined here. However, this does indicate an important potential caveat that must be considered when addressing the prebiotic availability of C and U nucleobases/sides/tides [see, e.g., Todd et al. (58)]. Photohydration could also explain the differing rates determined for cytosine vs. cytidine and uracil vs. uridine. When the sugar is present in the nucleosides, the hydroxyl group on the 5^′^ carbon could attack the 5–6 double bond in the pyrimidine nucleobase to form an intramolecular cyclic structure. Powner et al. (19) postulated a similar structure for the product of irradiation of β-D-ribocytidine-2^′^,3^′^-cyclic phosphate, leading to potentially different rates of photodegradation between the nucleobase and nucleoside/tide, and the apparent difference in photohydrate formation marked in their respective UV–Vis spectra. Determining these potential mechanisms of photohydration, however, is beyond the scope of this study.

Considering their abundance in extant biology, one possible explanation for the prevalence of cytidine and uridine could be that other chemical and/or physical factors contributed to the selection of these molecules, such as UV shielding—a phenomenon that has been previously observed among other prebiotically relevant chemical species (16, 59, 60). Other prebiotic mechanisms like encapsulation events, such as lipid vesicles (61, 62) or hydrothermally derived rock pores (63, 64), may have played a role in the protection and preservation of these molecules, though this is still an area of active research. Additionally, some prebiotic chemical scenarios favor a UV-free environment (65, 66), which would render this seeming contradiction resolved. Some suggest that prior to the RNA world, a period of precursor molecules assembling into primitive RNA existed, known as the pre-RNA world (67–69). In this prebiotic scenario, the molecular layout of life could have started off with different molecules, then progressively switched individual components with time and eventually led to the present-day molecular layout. In the lines of this idea, 2-thiouridine has been suggested to have acted as a structural ancestor to uridine due to its RNA strand thermodynamic-stabilizing properties (70) and its capability to improve the rate and fidelity of nonenzymatic RNA primer extension (71). Furthermore, the presence of cytidine and uridine in extant life may have been facilitated via the thiolysis of α-anhydrocytidine with formamide present to form α-2-thiocytidine, which can then photoanomerize into β-2-thiocytidine with UV light (51, 72). Hydrolysis of β-2-thiocytidine ultimately yields cytidine and uridine. Because we only studied the noncanonical nucleobases 2-thiocytosine and 2-thiouracil, we cannot directly compare the photostabilities of 2-thiocytidine and 2-thiouridine to cytidine and uridine. However, comparing the rate constant ratio for the nucleobases, we see that cytosine is ≈2.4 times more photostable than 2-thiocytosine, and 2-thiocytosine is the fourth least photostable molecules, as seen in Figure 3. Interestingly, 2-thiouracil was shown to be more photostable than uracil and 2-thiocytosine, with rate constant ratios of ≈1.6 and 2.0, respectively, but less photostable than cytosine ($k_{\mathrm{cytosine}}/k_{2-\mathrm{thiouracil}}\approx 0.85$).

The noncanonical nucleobases and nucleosides tested here had a wide range of observed photodegradation constants, from ≈10^−3^ min^−1^ (melamine) to ≈10^−1^ min^−1^ (2-pyrimidinone). Melamine, a 1,3,5-triazine heterocycle, was the most photostable noncanonical molecule investigated in this study and the second most photostable molecule overall. Melamine is of potential interest for prebiotic chemistry due to its ability to form supramolecular noncovalent assemblies with barbituric acid (47) and cyanuric acid (73, 74). Cyanuric acid and modified 2,4,6-triaminopyrimidine have also been shown to spontaneously form supramolecular assemblies (74) that have been suggested as possible alternative genetic polymers to DNA and RNA. We find cyanuric acid, barbituric acid, and 2,4,6-triaminopyrimidine to be less photostable than melamine by factors of ≈6.7, 50, and 48, respectively.

Inosine, xanthosine, and 2,6-diaminopurine are structurally similar to the canonical purine nucleosides (adenosine and guanosine) and have roles in extant biology. Inosine is produced by the deamination of adenosine, where the exocyclic amine group in C(6) of the adenosine is removed and C(6) is then converted to a ketone group. Inosine commonly replaces adenosine posttranscriptionally in the wobble positions of tRNA anticodons, where it can attach to a variety of codons in mRNA counterparts, driving translation flexibility and increasing tRNA diversity (75). Given the structural similarities between inosine and guanosine, how inosine resembles guanosine in biochemical systems (32, 76), and how inosine improves the fidelity of nonenzymatic RNA replication (77), inosine has been suggested to have served as a structural ancestor to guanosine in primitive RNA (38, 77). 2,6-diaminopurine has been shown to replace adenine in the genetic alphabet of some bacteriophages (78). In resemblance to studies on inosine, 2,6-diaminopurine has been shown to improve the rates of nonenzymatic RNA oligomerization when replacing adenine (79). Szabla et al. (80) additionally found that 2,6-diaminopurine is capable of repairing CPDs where, considering its likely availability on the prebiotic Earth, it may have provided beneficial effects on surrounding photodamaged RNA and DNA strands. Xanthosine, an important molecule in purine metabolism in extant biology, can be formed through deamination of guanosine. Past work has shown that issues with purine metabolism can lead to significant incorporation of xanthine and hypoxanthine into nucleic acids (81). The properties of xanthosine-containing RNA were studied by Mair et al. (82).

Interestingly, we find that adenosine and guanosine are more photostable than these noncanonical purine nucleosides. 2-aminoadenosine is the most photostable among the noncanonicals, with inosine following, then xanthosine. Adenosine is ≈8.1 times more photostable than guanosine; ≈11 times more photostable than 2-aminoadenosine; ≈12 times more photostable than inosine; ≈22 times more photostable than xanthosine. Among the purine nucleosides, it does appear as if the canonical letters used in life today are more photostable than noncanonical purine derivatives.

We can compare our eight nucleobase and nucleoside pairs to assess if there are patterns in the relative stability of these categories of molecules. In four cases, the nucleobase is more stable than the corresponding nucleoside (uracil/uridine, cytosine/cytidine, hypoxanthine/inosine, xanthine/xanthosine). In the other four cases, the nucleoside is more stable than the nucleobase (adenosine/adenine, thymidine/thymine, 2-amino-adenosine/2,6-diaminopurine, zebularine/2-pyrimidinone). Overall, there is not a clear trend in the photostability of nucleobases versus nucleosides.

The molecules used in this study belong to three broad classes: pyrimidines, purines, and triazine derivatives. We can compare the general trends in photostability among these three classes of molecules. In general, pyrimidine molecules are observed to be less photostable than purine molecules. Of the 24 molecules for which we measured rate constants here, nine are purines, twelve are pyrimidines, and three are triazines or triazine-derivatives. Ten of the pyrimidines are in the 12 most photounstable molecules, while seven purines are found in the 12 most photostable molecules. The three triazine derivatives fall into the 12 most photostable molecules. In general, it appears as though pyrimidine molecules are less photostable than purines. This trend is also observed for the canonical nucleobases and nucleosides, where the pyrimidines (C, U, and T) are less photostable than the purines (A and G). Thymine/thymidine being significantly more photostable than uracil/uridine raises interesting prebiotic implications. If UV light was indeed a factor in the selection of the genetic alphabet, then the resulting degradation rate constants suggests that thymine/thymidine could have been selected over uracil/uridine due to their higher photostabilities ($k_{\mathrm{uracil}}$/$k_{\mathrm{thymine}}\;\approx \;7.1$ and $k_{\mathrm{uridine}}/k_{\mathrm{thymidine}}\;\approx \;67$). This may have influenced the transition from uracil/uridine to thymine/thymidine in the case where a transition from an RNA world toward an environment dominated by the presence of DNA happened, or an environment where the concomitant synthesis of RNA and DNA occurred (72).

5-methylcytosine and 5-methylcytidine are cytosine- and cytidine-methylated derivatives that can be commonly found in both DNA and RNA, and are major epigenetic modifications that have been linked to many biological mechanisms, such as gene expression (83). Additionally, the photochemical deamination of 5-methylcytosine can result in the canonical nucleobase thymine (84). Hoehn et al. (55) compared the photostability of 5-methylcytidine against cytidine using femtosecond transient absorption spectroscopy, finding that 5-methylcytidine is three times more photostable than cytidine upon UV irradiation. We performed multiple UV irradiation experiments for 5-methylcytosine and 5-methylcytidine attempting to measure their photodegradation rate constants. However, the kinetics of photodegradation of these molecules cannot be easily monitored by UV–Vis spectroscopy due to shifting absorption features throughout the course of the irradiation. Rather, the UV–Vis spectra (*SI Appendix*, Figs. S49 and S50) for both molecules indicate potentially multiple products with competing absorption features produced upon UV irradiation. Privat and Sower (84) showed that the UV irradiation of aqueous 5-methylcytosine could undergo a variety of UV-induced reactions which could yield a plethora of photoproducts. If UV irradiation results in a photoproduct with significant absorption in a similar wavelength range as the initial molecule, quantification of the rate of degradation will be difficult. Because such mechanisms and the characterization of photoproducts are outside of the purpose of this study, rate constants for 5-methylcytosine and 5-methylcytidine were not reported.

Previous authors have investigated the photodynamics of various canonical and noncanonical nucleobases, nucleosides, and nucleotides. Ortiz-Rodríguez et al. (85) used density function theory calculations to predict the photoabsorption and photoexcitation pathways of cyanuric acid. They find that cyanuric acid has an absorption maximum at ≈160 nm, with a tail extending to 200 nm. From the quantum chemical calculations of the excitation states and pathways to return to the ground state, cyanuric acid was predicted to be highly photostable under the UV-environment likely to be present on the early Earth (85). Rankine (86) investigated the excited-state dynamics of 2,4,6-triaminopyrimidine using quantum chemical calculations. They find a short excited-state lifetime comparable to the canonical pyrimidine nucleobases, which they state makes 2,4,6-triaminopyrimidine likely to be of comparable superior photostability as the canonical nucleobases, as indicated from the short excited-state lifetimes and predicted fast internal conversion to the ground state. Pepino et al. (87) studied the ultrafast dynamics of the canonical pyrimidines (thymidine, uridine, and cytidine) via quantum chemical calculations; they find a number of decay paths with ultrafast timescales but do not explicitly consider longer-lived decay channels. The excited-state lifetime of barbituric acid was calculated by Romeo-Gella et al. (88) and found to be sub-picoseconds, which is similar in magnitude to the other canonical pyrimidines. The ultrafast photophysics of barbituric acid and 2,4,6-triaminopyrimidine were investigated experimentally by Brister et al. (89), who found short lifetimes on the order of picoseconds for various transient absorption features in both molecules, which they claim supports the plausible photostability of these molecules. The excited-state dynamics of melamine were considered by Zhang et al. (90) by experimental transient absorption spectroscopy. Most excited states were found to decay with timescales of approximately 13 ps, which is suggested to be short enough to confer melamine with a significant amount of photostability. Femtosecond transient absorption spectroscopy of inosine, hypoxanthine, and inosine monophosphate revealed excited-state lifetimes of ps or less for all three molecules (91). The excited-state dynamics of 2,6-diaminopurine were experimentally determined by femtosecond transient absorption spectroscopy to be sub-ps (92). Measurements of the nucleotide xanthosine monophosphate reveal an excited-state lifetime of ≈1 ps (93). Zebularine has been suggested to undergo intramolecular hydrogen abstraction upon photoexcitation to a triplet state (94). Mai et al. (95) investigated the photodynamics of 2-thiouracil using quantum chemical calculations; excitation to a singlet or triplet states are possible. The excited singlet state (S_2_) is predicted to de-excite back to S_1_ in short timescales (200 fs); triplet states are populated via intersystem crossing on a similar timescale of a few hundred femtoseconds (95). At 1 ps, the calculations show the majority of the population is in the triplet state. The photochemistry of 2-thiocytosine has been studied previously through both experimental and theoretical methods (95, 96). Efficient conversion to the long-lived triplet state is observed, though solvation is suggested to play a significant role in the excited-state photodynamics of 2-thiocytosine (96, 97).

The canonical nucleobases and nucleosides have been suggested to have enhanced photostability due to their ability to effectively de-excite through internal conversion processes with various conical intersections. Excited-state lifetimes of the canonical nucleobases and nucleosides are reported to be on the order of picoseconds [see Crespo-Hernandez (54), Kufner et al. (98), Beckstead et al. (52) and references therein].

While there is a vast amount of literature studying the ultrafast photodynamics of many canonical and noncanonical nucleobases, nucleosides, and nucleotides, it should be noted that many of the experimental studies use differing excitation conditions and various theoretical studies utilize different methods and assumptions, which may create difficulties when comparing among different molecules. Our study mitigates this issue by systematically and consistently measuring the degradation of aqueous noncanonical and canonical nucleobases and nucleosides. Furthermore, we investigate photostability under continuous UV irradiation by monitoring the degradation of the aqueous parent molecule by UV–Vis spectroscopy, which presents additional information arguably more relevant for planetary conditions than using excited-state lifetimes to infer photostability.

One major caveat is that the 254 nm irradiation used in our experiments is not necessarily representative of the UV environment (total power and spectral flux) expected on the early Earth. Although the 254 nm mercury lamps are experimentally practical, the early Sun output a range of UV flux from approximately 200 to 300 nm during the estimated times of the emergence of life (8, 9). Photochemical reactions rates can depend on the wavelength of the irradiation, e.g., refs. 3, 16, and 58, with generally faster rates of photoreactions occurring when the irradiation wavelength overlaps with an absorption band in the molecule. The canonical nucleobases and nucleosides all have similar absorption features ($\lambda_{\max}$ ranging from 252 to 271 nm) consistent with the 254 nm irradiation used experimentally here. Some of the noncanonical nucleobases and nucleosides investigated in this study had absorption maxima distinctly different from this wavelength (e.g., melamine, $\lambda_{\max}=203$ nm; urazole, $\lambda_{\max}=200$ nm; and 2-thiocytosine, 2-thiouracil, 2-pyrimidinone, and zebularine, which showed broader absorption features extending out to >280 nm). It would thus be reasonable to expect that melamine and urazole would be more efficiently degraded by shorter wavelengths of UV light [following wavelength-dependent irradiation behaviors as seen in Todd et al. (16)], though these wavelengths would likely be less present on the surface of the early Earth.

**Fig. 4.**
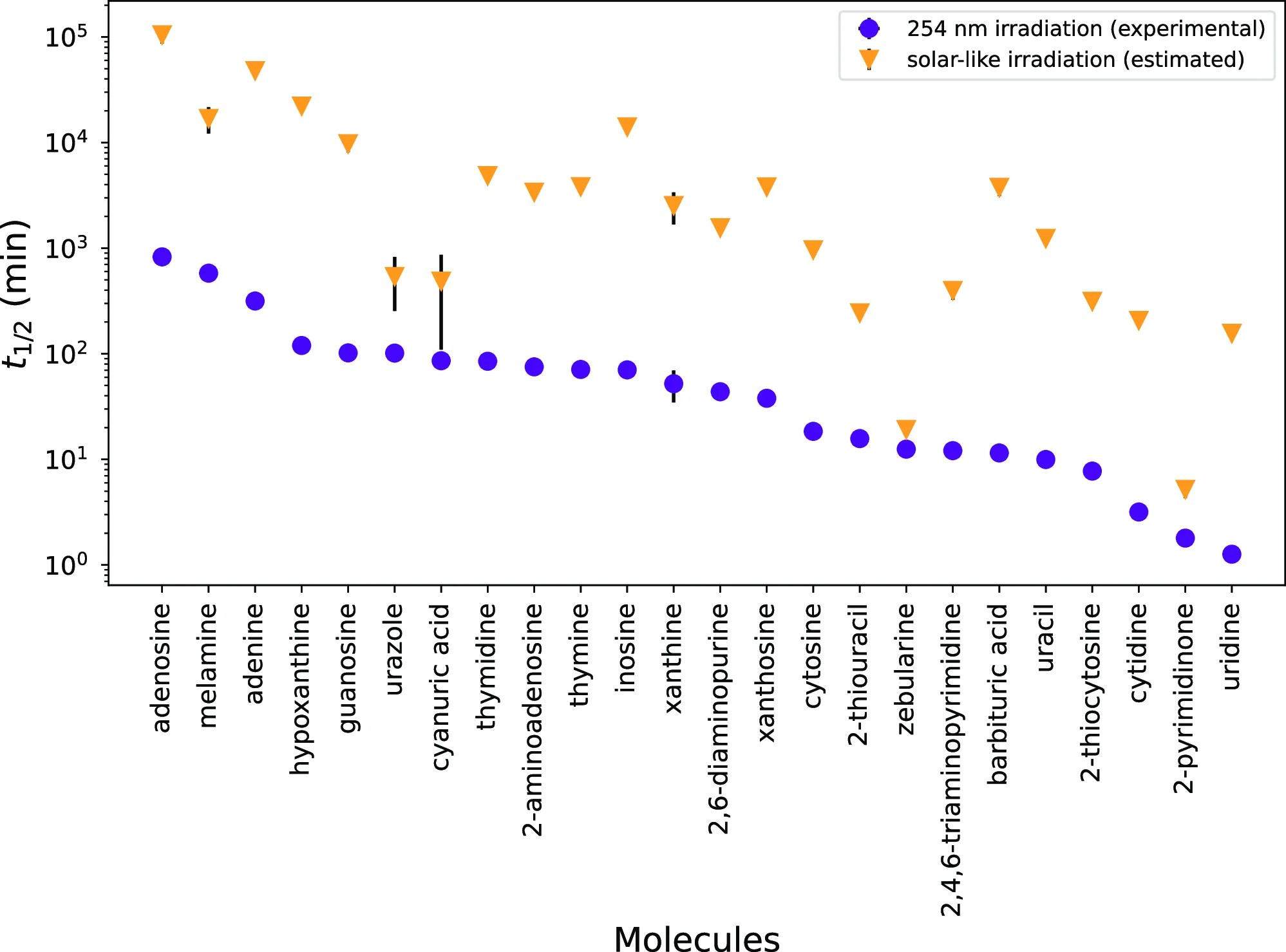
Half-lives t_1/2_ for our experiments carried out at 254 nm in the Rayonet RPR-200 (purple) and approximate values estimated under a plausible early Earth UV environment (orange, see Eq. **1**). Ordered from most stable (*Left*) to least stable (*Right*) under experimental conditions.

To address the possible changes in photostabilities of molecules under a more realistic UV environment, we have calculated an estimated total photodegradation rate constant for each molecule under the expected surface intensity of UV irradiation on the early Earth ($k_{\mathrm{tot}}$) as follows:

[1] $$\begin{array}{c} k_{\mathrm{tot}}=k_{254}\frac{\int_{200}^{400}A_{\lambda }SI_{\lambda }d\lambda }{I_{0,254}A_{254}}, \end{array}$$

where $k_{254}$ is our experimentally determined photodegradation rate constant under 254 nm irradiation, $A_{\lambda }$ is the wavelength-dependent absorbance of a given molecule, $SI_{\lambda }$ is the surface intensity estimated on the surface of the early Earth through a sample N_2_/CO_2_ atmosphere (8, 9, 99), $I_{0,254}$ is the incident photon flux in our irradiation experiments determined by chemical actinometry, and $A_{254}$ is the absorbance of each molecule at 254 nm. Since the absorption spectra were measured from 200 to 400 nm, this necessarily sets the wavelengths we integrate over (see *SI Appendix*, section VII for details). From the degradation rate constants, we calculate the half-lives for each molecule under our 254 nm irradiation experimental setup and under a more prebiotically realistic UV environment (Fig. 4). We see general agreement in the photostability of molecules under both conditions, but with a few notable outliers. In particular, those molecules with absorption peaks far from 250 to 260 nm show differences in expected photostability under the UV environment expected on the early Earth. Molecules such as zebularine and 2-pyrimidinone, with absorption maxima at longer wavelengths (≈300 nm) have decreased half-lives under early Earth UV irradiation. This can be rationalized because of the larger photon fluxes at longer wavelengths, which inflates the estimated rate constant under solar-like UV irradiation. Similarly, some molecules with absorption peaks near 200 nm also show decreases in estimated photostability under broadband UV irradiation compared to other molecules. These molecules generally absorb minimally at 254 nm, so the experimentally determined rate constants at this wavelength may overestimate their photostability. Though there are comparatively fewer photons expected on the surface of the early Earth at these shorter wavelengths, when taken into account with absorbance spectra in our calculation, we see elevated comparative estimated rates of photodegradation for these molecules.

It is important to note that this calculation involves several important assumptions: 1) wavelength-dependent photodegradation rates will be proportional to the absorbance spectrum of a molecule; 2) photons of all wavelengths lead to the same channel of photodegradation. The first assumption has generally been found to be true (e.g., refs. 3, 16, and 58). The second assumption may not necessarily be true. Many photochemical reactions are known to undergo different channels based on irradiation wavelength (e.g., ferrocyanide is photooxidized at λ<300 nm and photoaquated at λ>300 nm, ref. 100 and references therein.) Given these assumptions, our estimated total rate constants under an early Earth UV environment should be treated with caution. Experimental tests of photodegradation under broadband irradiation should be carried out to validate these estimates; indeed, future work is planned to do so.

Last, it is also important to emphasize that shielding factors, such as water column depth and composition (60), complex dark/light chemistry cycles (4), the presence of other prebiotically relevant species (101), and concentration-dependent self-shielding (59, 100) are not under the scope of this study, but could influence the relative photostabilities and half-lives of these molecules under planetary conditions.

## Conclusions

In this study, we measured the rate constants of UV-driven photodegradation of various canonical and noncanonical nucleobases and nucleosides. Since more than just the five canonical nucleobases and nucleosides were possibly available on the early Earth, it is unclear what mechanism may have led to the genetic alphabet seen in life on Earth today. One possibility has suggested that selection due to UV photostability may have played a role, with some evidence from the generally shorter excited-state lifetimes of canonical nucleosides compared to various modified nucleobases and nucleosides. However, this hypothesis had not yet been systematically addressed under continuous UV irradiation conditions, which we have done here. We did not find clear indications that the canonical nucleobases and nucleosides have longer lifetimes under continuous UV irradiation when compared to various noncanonical analogs. Instead, the story may be more complicated, potentially invoking a variety of chemical (e.g., intrinsic photostability) and physical (e.g., environmental/geochemical) properties. Alternatively, it is possible that the selection of the canonical nucleobases and nucleosides was due to alternative reasons, perhaps due to their ability to undergo RNA copying, the yields that are available under planetary conditions, or maybe even just random chance.

## Definitions

- **Nucleobase**: Heterocyclic, single-, or double-ringed molecule with nitrogen. Canonical nucleobases are those used in biology—these include adenine, guanine, cytosine, thymine, and uracil.
- **Nucleoside**: Nucleobase unit attached to a sugar molecule. The canonical nucleosides used by biology consist of the canonical nucleobases attached to deoxyribose (in DNA) or ribose (in RNA).
- **Nucleotide**: Nucleobase unit attached to sugar and a phosphate group. Nucleotides are the basic units of RNA and DNA used in Earth biology.
- **Canonical nucleobase/side/tide**: Basic units of nucleic acids used by Earth biology—e.g., in DNA, the canonical nucleosides are adenosine, guanosine, cytidine, and thymidine. In RNA, uridine replaces thymidine.
- **Noncanonical nucleobase/side/tide**: Heterocyclic molecules similar in structure to canonical nucleobases/sides/tides, but not found in biology as part of the genetic alphabet in DNA or RNA.

## Supplementary Material

Appendix 01 (PDF)

## Data Availability

Data and analysis scripts are available in Zenodo (https://doi.org/10.5281/zenodo.20708893) (102). All other data are included in the manuscript and/or *SI Appendix*.
