# Supplementary material for: Continuous UV irradiation played an inconclusive role in selecting the canonical genetic alphabet
Source: Proc Natl Acad Sci U S A. 2026 Sep 28;123(41):e2602433123. doi: 10.1073/pnas.2602433123 (PMC13663812; doi:10.1073/pnas.2602433123)
Supplement: Supplementary file 1 [file pnas.2602433123.sapp.pdf]

Supporting Information

Continuous UV irradiation played an inconclusive role in selecting the canonical genetic alphabet

Marcos Jusino-Maldonado, William C. Platten, Henry J. Wolfe, and Zoe R. Todd

Table of Contents

I. Materials and Sample Preparation
II. Standard Curves
III. UV-Vis spectra and kinetics plots
IV. Rate constants and concentrations from individual experiments
V. pH of samples before and after irradiation
VI. Irradiation geometry and actinometry to determine photon flux
VII. Calculation of estimated photodegradation under a suggested UV environment on early Earth
VIII. Calculation of errors
IX. Penetration depths of 254 nm photons in samples
X. Dilution spectra of all molecules
XI. References

I. Materials and Sample Preparation

5-methyl uracil (thymine, 97%), adenine hydrochloride (98%), cytosine (98%), uracil (98%), uridine (98%), melamine (98%), 2,6-diaminopurine (98%), zebularine (95%), urazole (98%), inosine (98%), 5-methylcytosine (97%), 5-methylcytidine (97%), and cyanuric acid (98%) were purchased from Combi-Blocks. Thymidine (98%), guanosine (98%), adenosine (99%), cytidine (99%), 2-thiocytosine (97%), 2-thiouracil (98%), hypoxanthine (99%), and xanthine sodium salt (99%) were purchased from Sigma Aldrich. Barbituric acid (99%) was purchased from Beantown Chemicals, xanthosine dihydrate (99%) was purchased from Thermo Scientific, and 2,4,6-triaminopyrimidine (99.99%) was purchased from Ambeed. 2-hydroxypyrimidine hydrochloride (98%) and 2-aminoadenosine (98%) were purchased from TCI Chemicals. All chemicals were used without further purification.

10, 15, or 20 mM stocks for each molecule were initially prepared by weighing commercially-available reactants using a Sartorius 1702 analytical balance and dissolving with ultra-pure water purified by a Thermo Scientific Barnstead GenPure water purification system to obtain the stock solution concentrations (see Table S1). Original 10 – 20 mM stocks were diluted with ultra-pure water to generate 1 mM samples. From these 1 mM samples, three 50 μM, 3 mL samples were prepared for each molecule by mixing 150 μL of the 1 mM sample with 2850 μL ultra-pure water. When initial UV-Vis measurements indicated these solutions deviated significantly from the intended 50 uM concentrations (according to our standard curves), adjustments were made to achieve the desired concentration as determined by absorption and standard curves. The initial pH for each molecule was measured using an Apera Instruments PH700 pH meter, and an average initial pH was calculated (see Table S5). We tested pH measurements before and after sparging the samples with N_2 (g) to determine whether sparging affected the pH of the solutions and found no significant pH differences. All samples were sparged with N_2 (g) at 1 minute per 1 mL of sample (3 minutes total) to remove potential influence from O_2 photolysis and subsequent radical generation on the photodegradation kinetics and to resemble the lack of diffused oxygen due to the anoxic Hadean-Archean eons. 1 mL of each sparged sample was transferred into 1 mL quartz cuvettes (Starna cells part # 9-Q-10-GL14-C), and their initial UV-Visible spectra were measured using an Agilent Cary-60 UV-Visible spectrophotometer. After the final irradiation timesteps, the triplicate volumes from the quartz cuvettes were transferred into a separate container, and the triplicate, average final pH was measured. SI section V contains Table S5 with the initial and final pH values and descriptions of control experiments conducted to ensure pH variations do not significantly influence the degradation rates of molecules.

Melamine, due to its higher extinction coefficient at the maximum wavelength λ_{max} = 203 nm, was irradiated at ~25 μM concentrations to ensure absorbance values not greatly exceeding 1.0, to stay within the linear region of the UV-Vis detector (see Figure S40 for more details). Although guanine is a canonical nucleobase, it was not probed in this study due to its significantly low solubility in water, which renders its aqueous solution preparation inefficient. Because cyanuric acid shows a pH-dependent UV absorbance, we adjusted the solution pH to 7.3 using 0.1 M NaOH. All solutions were irradiated for fixed periods of time with a Rayonet RPR-200 Photoreactor with 254 nm lamps. Samples were placed in a RMA-500 sample holder, which was spinning during experiments to allow for uniform irradiation of all samples. The photon flux incident upon samples in this

geometry was calculated by actinometry to be (1.67 ± 0.033) x 10^{16} $\frac{photons}{s \cdot cm2}$, as described in SI Section VI.

Due to the observed varying rates of degradation in different molecules, we chose appropriate irradiation timesteps for each molecule to enable accurate determination of their rate constants. Total irradiation times ranged from a minimum of 90 seconds to a maximum of 40 minutes for the most photo-unstable and photostable molecules, respectively.

The UV-Vis spectra were measured from 200 - 400 nm using an Agilent Cary-60 UV-Vis spectrometer throughout the irradiation to measure the kinetics of the reactions. The absorbance at each timepoint was converted to a concentration by using standard curves (see Table S2 and Figures S1-S24). Plots of ln(concentration) vs. time were observed to be initially linear, which is consistent with first order or pseudo-first order kinetics. The slope of the trendline fitting ln(concentration) vs. time is equal to the negative rate constant, k, of the reaction, assuming first order kinetics. In some cases, non-linear behavior was observed at later timepoints during the irradiation. In these cases, we truncated data to the initial linear portion and used this to find the rate constants. See SI section III for a detailed description of the criteria for truncation. Rate constants were determined from sets of triplicate experiments for each molecule. Averages and standard errors were determined from the replicates. To determine the total error in the rate constants and concentrations, we combined the errors from the triplicate experiments with an estimated 7% error from pipettes, a 5% error from weighing out solids with the balance, and a 1.97% error from the photon flux. Errors were propagated as described in SI section VIII. The half-lives, $t_{1/2}$, were calculated using expression below for first-order kinetics, where k is the average rate constant experimentally measured here:

$$t_{1/2} = \frac{ln(2)}{k}$$

Table S1: Masses measured and volumes used for stock solutions of each molecule.

**Molecule**	**Weighted (grams)**	**Stock (H_2O) volume**
2,4,6-triaminopyrimidine	0.017	13590 µL (for **10 mM** stock)
2,6-diaminopurine	0.013	8661 µL (for **10 mM** stock)
2-aminoadenosine	0.0313	11100 µL (for **10 mM** stock)
2-hydroxypyrimidine	0.0141	10600 µL (for **10 mM** stock)
2-thiocytosine	0.013	10220 µL (for **10 mM** stock)
2-thiouracil	0.010	7803 µL (for **10 mM** stock)
5-methylcytidine	0.022	8554 µL (for **10 mM** stock)
5-methylcytosine	0.024	12790 µL (for **15 mM** stock)
Adenine	0.023	8936 µL (for **15 mM** stock)
Adenosine	0.024	8981 µL (for **10 mM** stock)
Barbituric acid	0.022	11450 µL (for **10 mM** stock)
Cyanuric acid	0.0212	10948 µL (for **15 mM** stock)
Cytidine	0.0116	4770 µL (for **10 mM** stock)
Cytosine	0.033	14851 µL (for **20 mM** stock)
Guanosine	0.020	7062 µL (for **10 mM** stock)
Hypoxanthine	0.0177	13000 (for **10 mM stock**)

Inosine	0.0153	5705 µL (for **10 mM** stock)
Melamine	0.026	12160 µL (for **15 mM** stock)
Thymidine	0.011	4540 µL (for **10 mM** stock)
Thymine	0.020	10570 µL (for **15 mM** stock)
Uracil	0.024	10700 µL (for **20 mM** stock)
Urazole	0.012	11870 µL (for **10 mM** stock)
Uridine	0.014	5733 µL (for **10 mM** stock)
Xanthine	0.014	8041 (for **10 mM** stock)
Xanthosine	0.016	4996 µL (for **10 mM** stock)
Zebularine	0.012	5259 µL (for **10 mM** stock)

II. Standard Curves

To convert the measured absorbances to concentrations when determining kinetics, we created standard curves for each molecule by performing a set of dilutions of known concentrations of the molecule and measuring the absorbance at the maximum wavelength. Dilutions for each molecule were repeated in triplicate. When performing the serial dilutions, only concentrations that gave a peak absorbance value below 1.0 were taken, to ensure we remained in the linear region of the UV-Vis spectrophotometer. Absorbances for each concentration were averaged together and the standard deviation was calculated. These averages were fit with a linear trendline (taking into account the errors on individual concentrations) using python to determine the relationship between concentration and absorbance at the peak wavelength. The standard curves for each molecule and their associated equations are presented below.

Table S2: Standard curve equations for each molecule, relating absorbance at the peak wavelength with concentration. All concentrations are in mM.

Molecule	Peak wavelength (nm)	Standard curve
2,4,6-triaminopyrimidine	270	$A_{270} = 12.7[2,4,6 - triaminopyrimidine] + 0.0808$
2,6-diaminopurine	280	$A_{280} = 9.64[2,6 - diaminopurine] - 0.000937$
2-aminoadenosine	280	$A_{280} = 10.4[2 - aminoadenosine] - 0.00288$
2-hydroxypyrimidine	299	$A_{299} = 4.54[2 - hydroxypyrimidine] + 0.000822$
2-thiocytosine	268	$A_{268} = 17.9[2 - thiocytosine] + 1.47 \times 10^{-5}$
2-thiouracil	274	$A_{274} = 15.1[2 - thiouracil] + 0.0197$

Adenine	261	$A_{261} = 12.8[Adenine] + 0.00250$
Adenosine	259	$A_{259} = 13.4[Adenosine] - 0.00946$
Barbituric acid	257	$A_{257} = 13.8[Barbituric\ acid] + 0.0771$
Cyanuric acid	213	$A_{213} = 6.90[Cyanuric\ acid] - 0.0411$
Cytidine	271	$A_{271} = 8.81[Cytidine] - 0.00526$
Cytosine	271	$A_{271} = 8.87[Cytosine] - 0.000472$
Guanosine	252	$A_{252} = 14.2[Guanosine] + 0.0189$
Hypoxanthine	249	$A_{249} = 9.28[Hypoxanthine] + 0.00437$
Inosine	249	$A_{249} = 12.5[Inosine] + 0.0109$
Melamine	203	$A_{203} = 39.0[Melamine] + 0.0210$
Thymidine	267	$A_{267} = 9.92[Thymidine] - 0.00432$
Thymine	264	$A_{264} = 8.79[Thymine] - 0.000336$
Uracil	258	$A_{258} = 9.73[Uracil] + 0.00138$
Urazole	200	$A_{200} = 6.11[Urazole] - 0.00653$
Uridine	262	$A_{262} = 8.91[Uridine] + 4.98 \times 10^{-5}$
Xanthine	268	$A_{268} = 7.66[Xanthine] + 0.0651$
Xanthosine	261	$A_{261} = 11.5[Xanthosine] + 0.106$
Zebularine	304	$A_{304} = 5.66[Zebularine] + 0.00437$

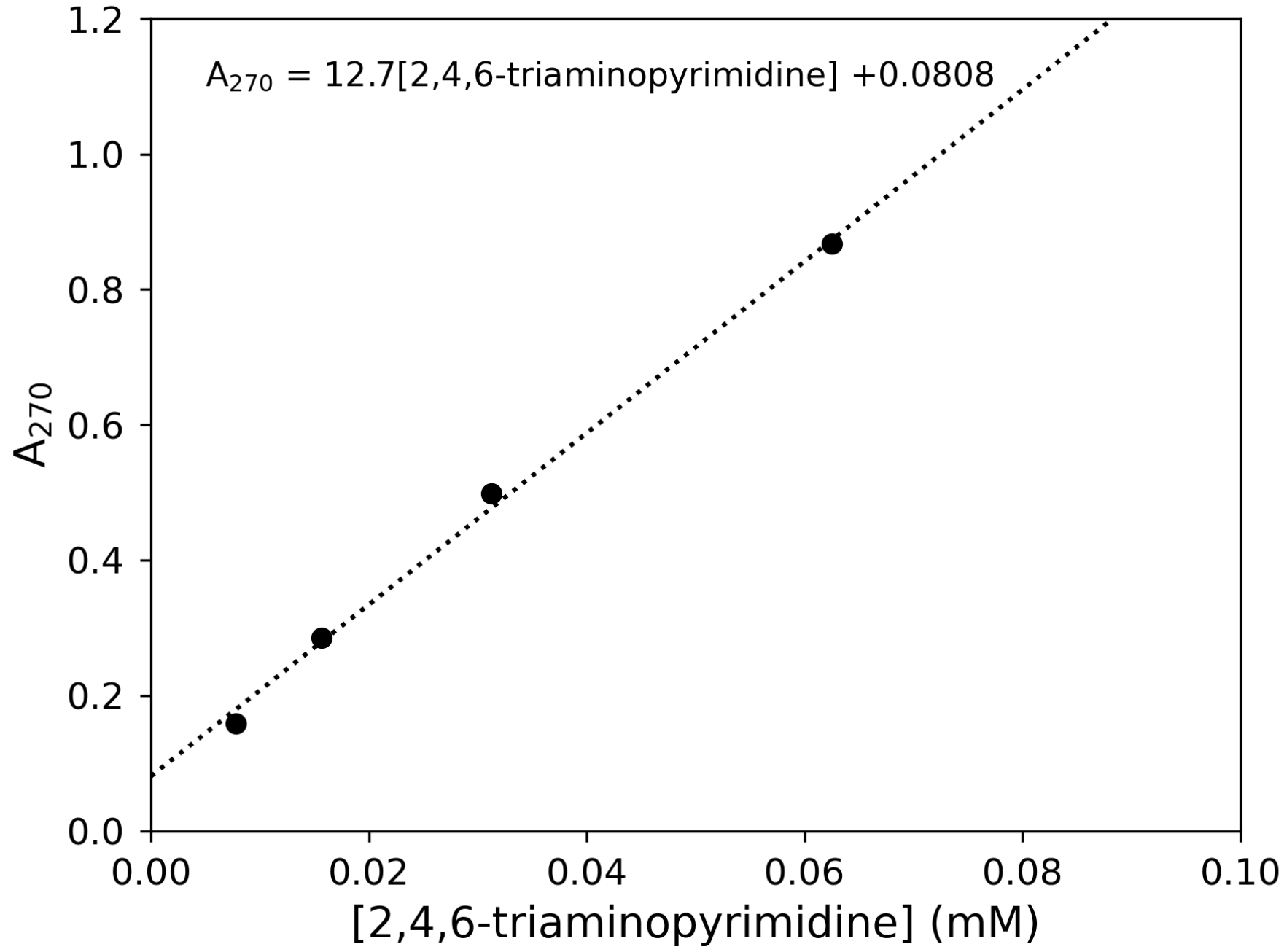

Figure S1: Standard curve for 2,4,6-triaminopyrimidine.

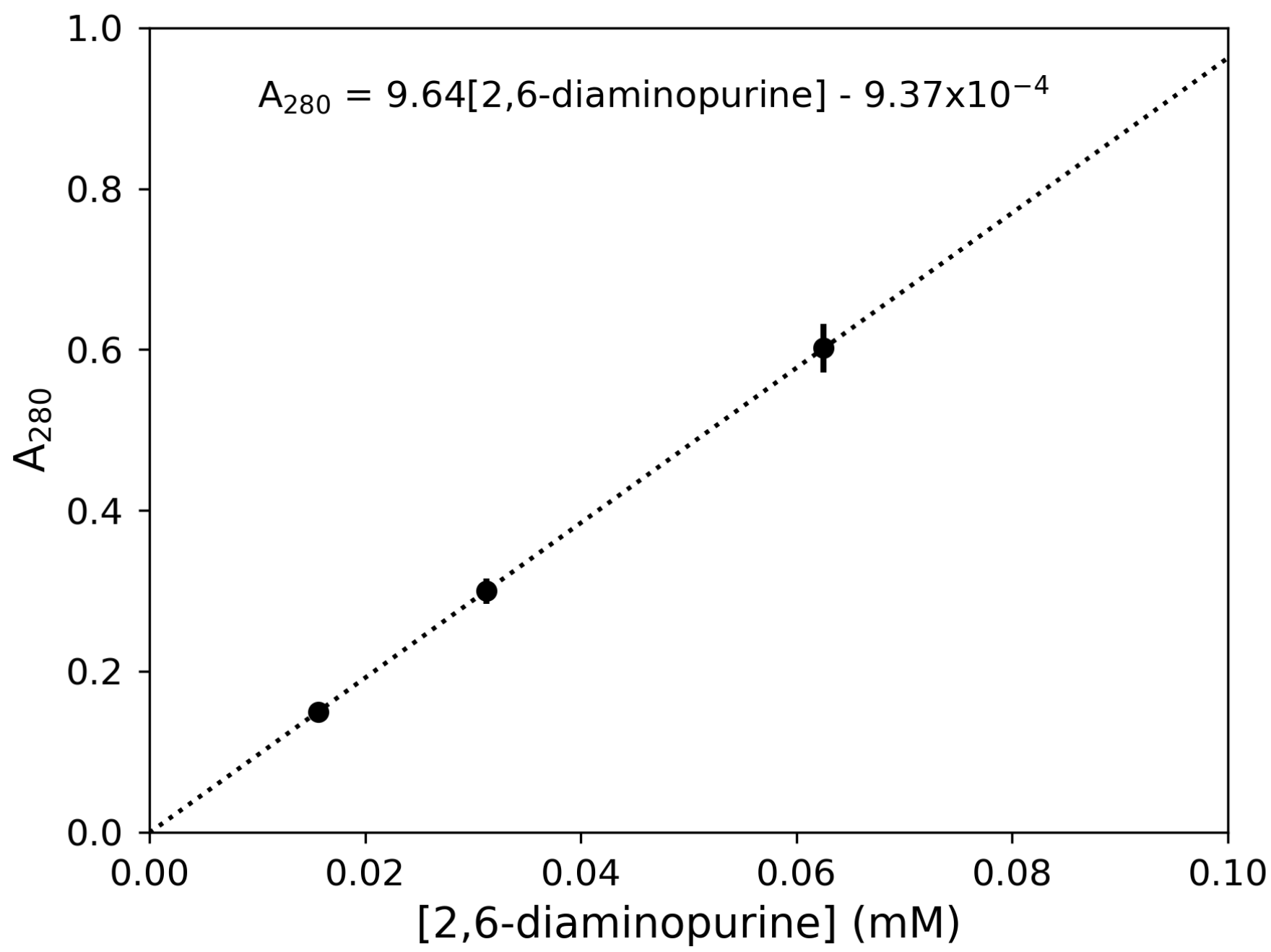

Figure S2: Standard curve for 2,6-diaminopurine.

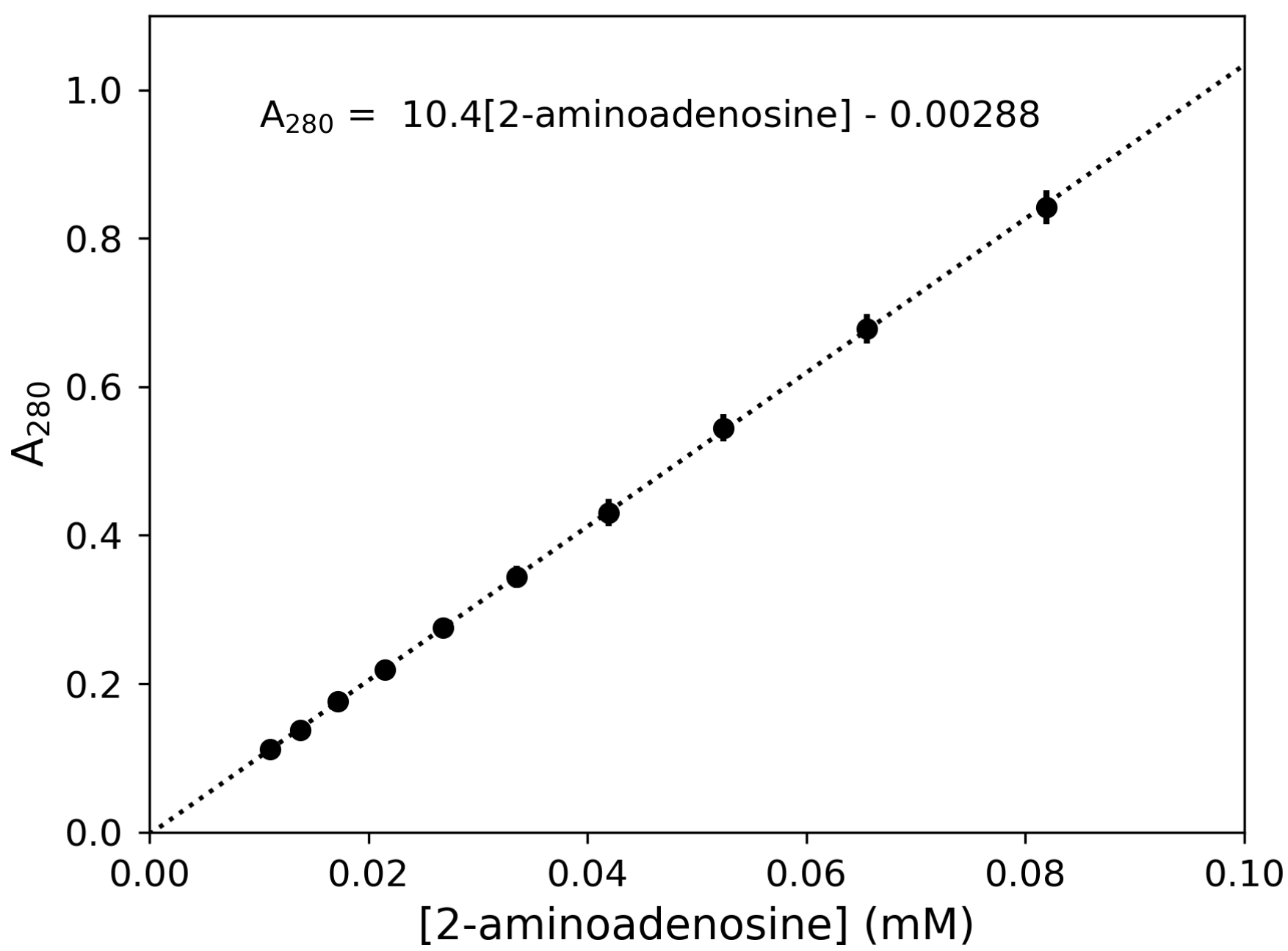

Figure S3: Standard curve for 2-aminoadenosine.

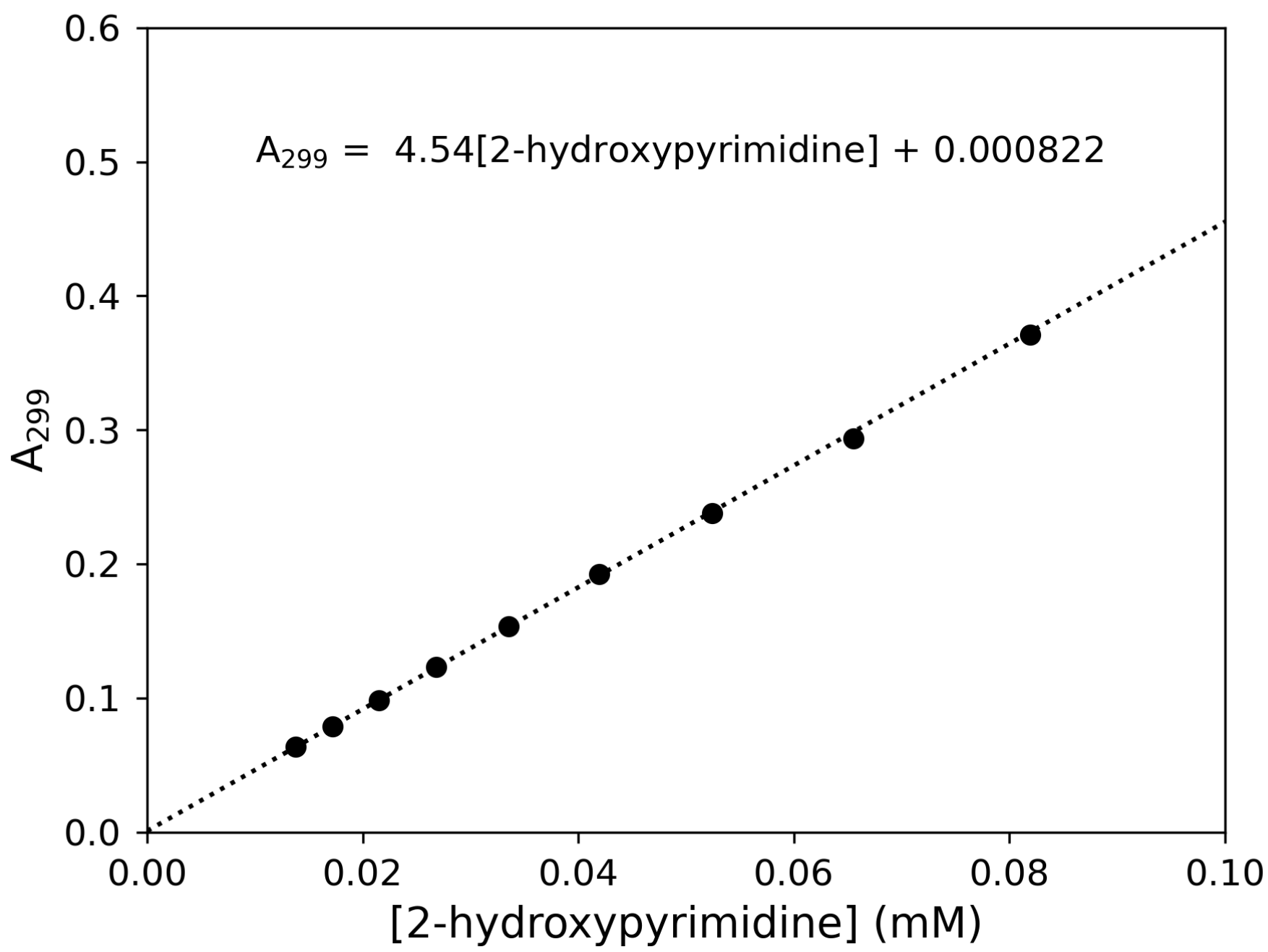

Figure S4: Standard curve for 2-hydroxypyrimidine (2-pyrimidinone).

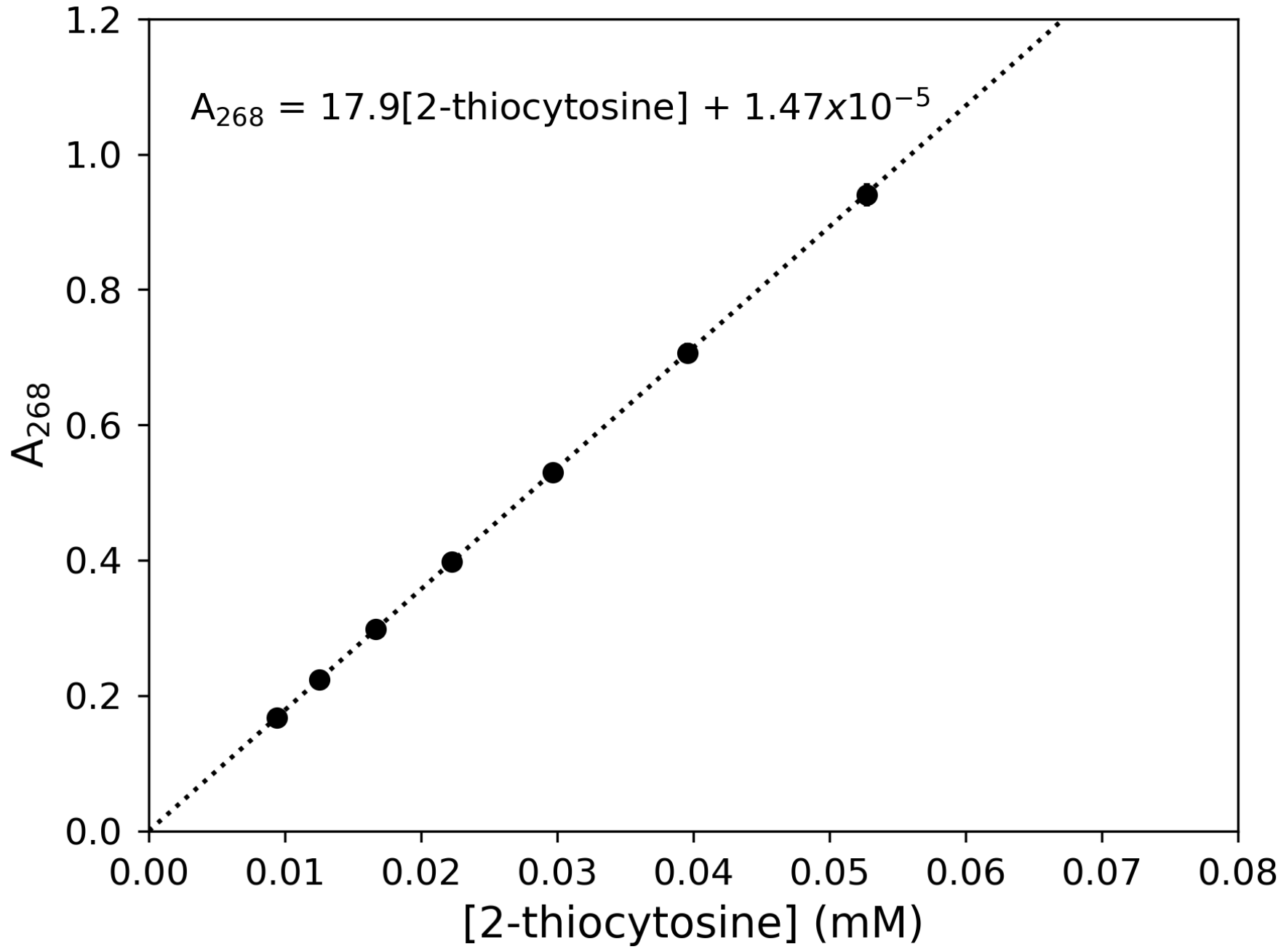

Figure S5: Standard curve for 2-thiocytosine.

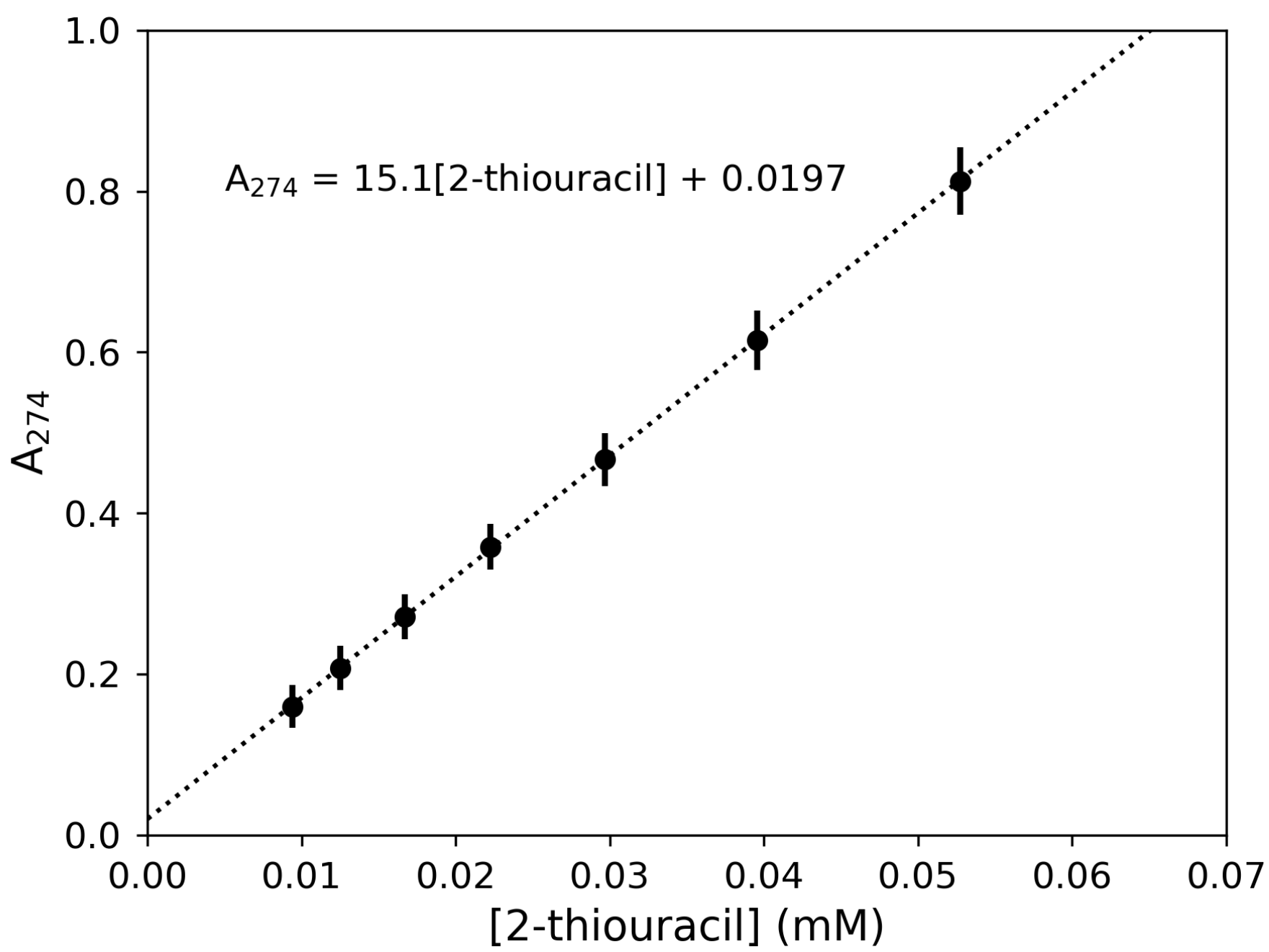

Figure S6: Standard curve for 2-thiouracil.

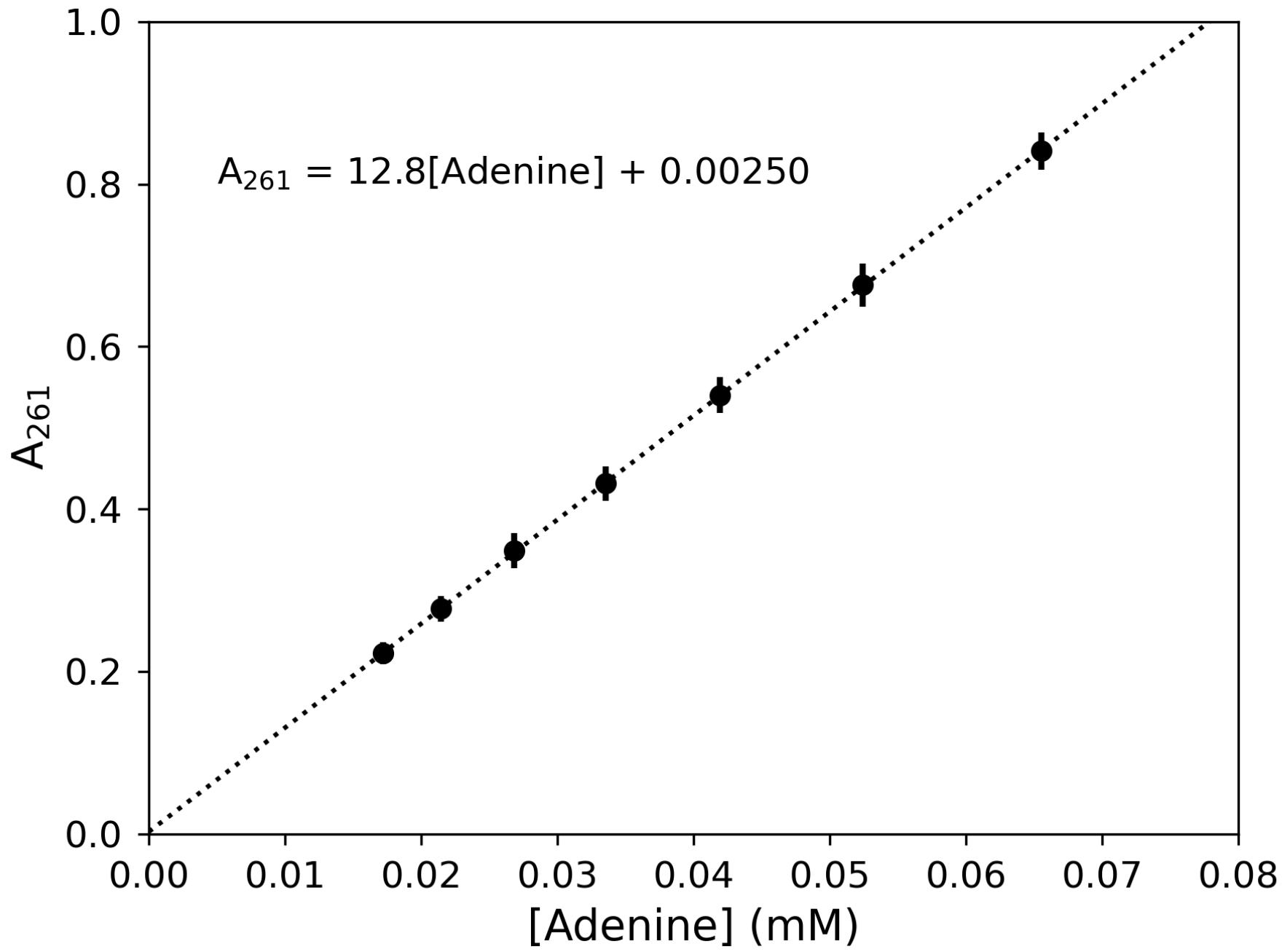

Figure S7: Standard curve for adenine.

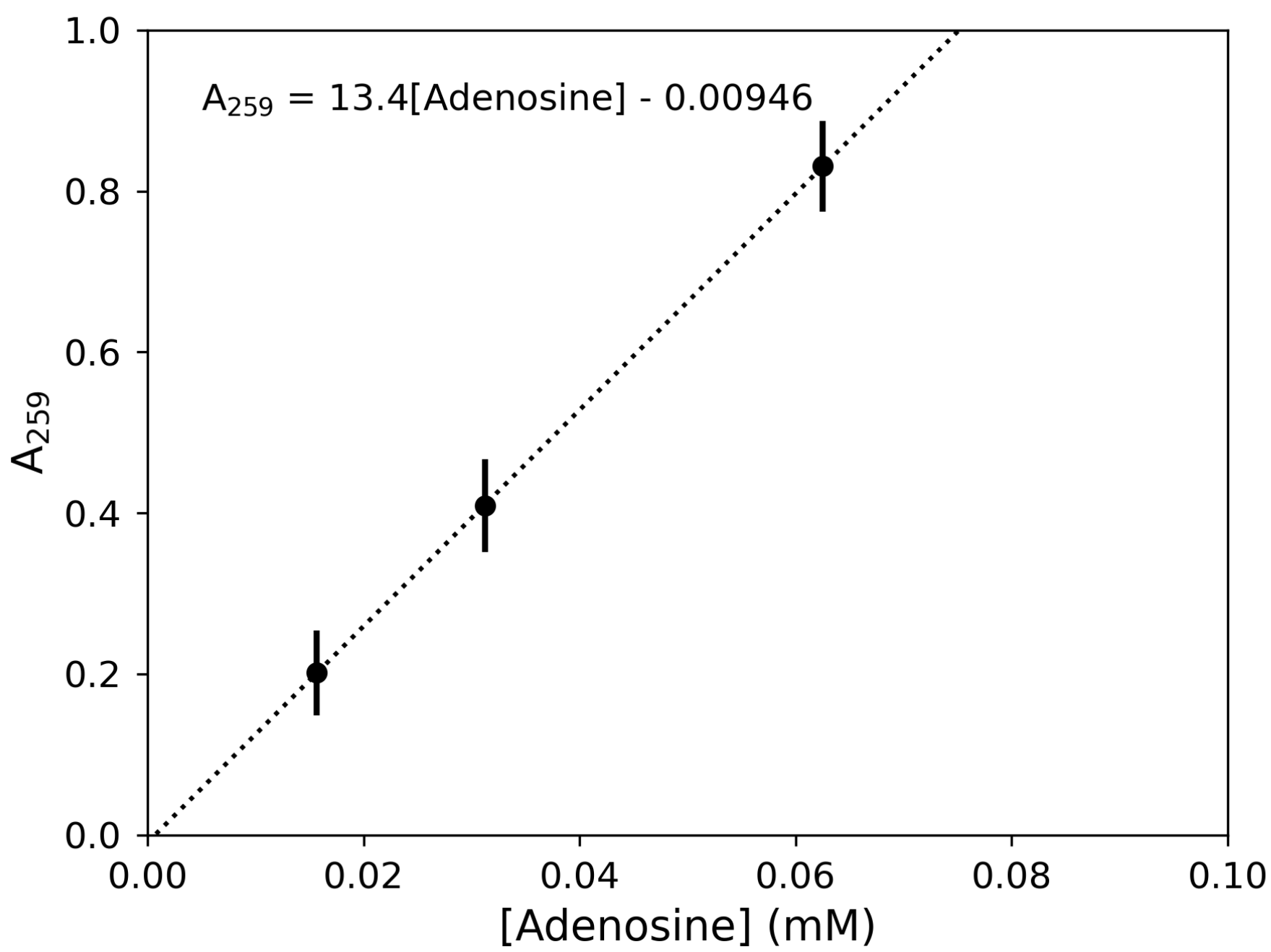

Figure S8: Standard curve for adenosine.

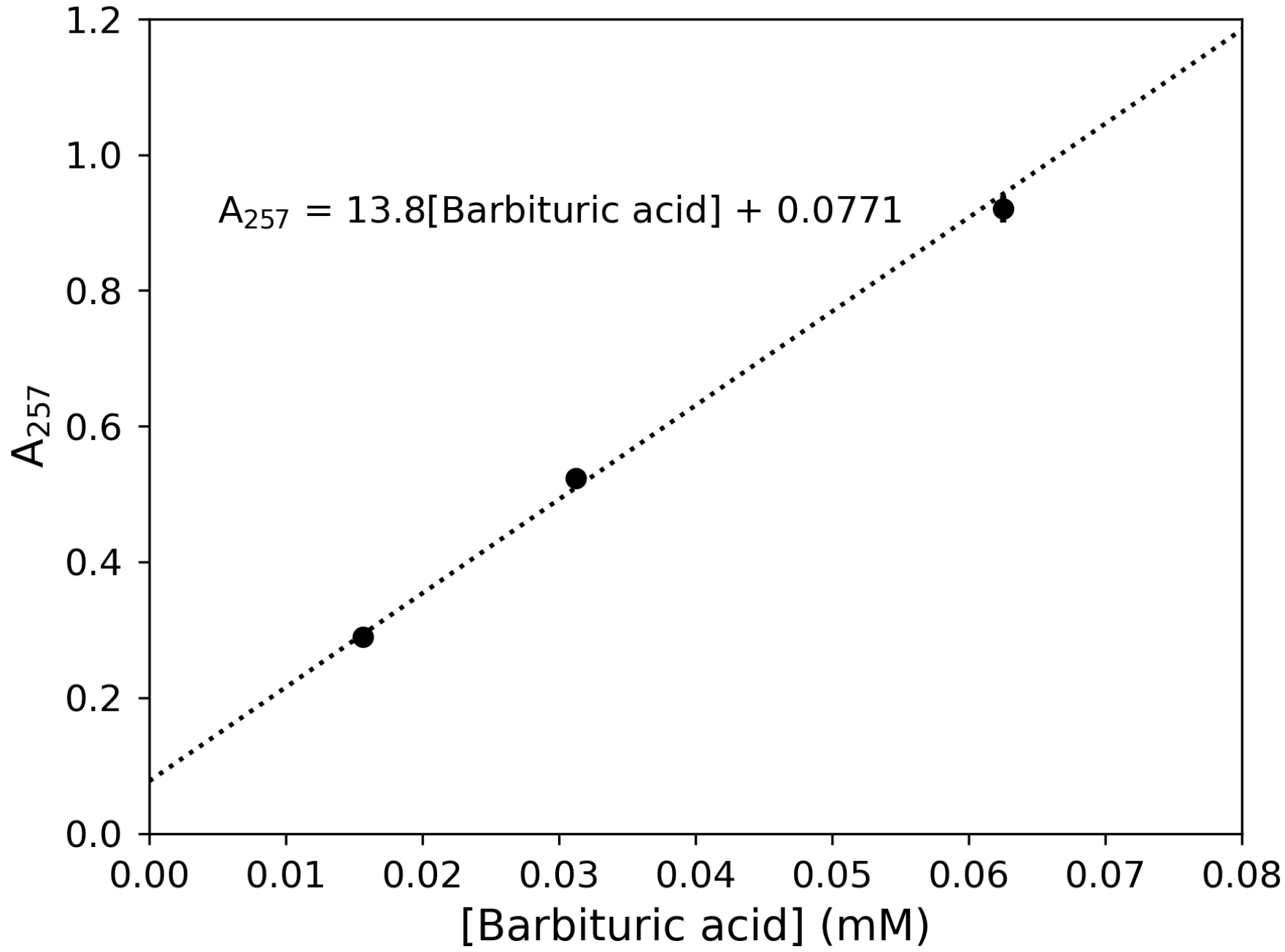

Figure S9: Standard curve for barbituric acid.

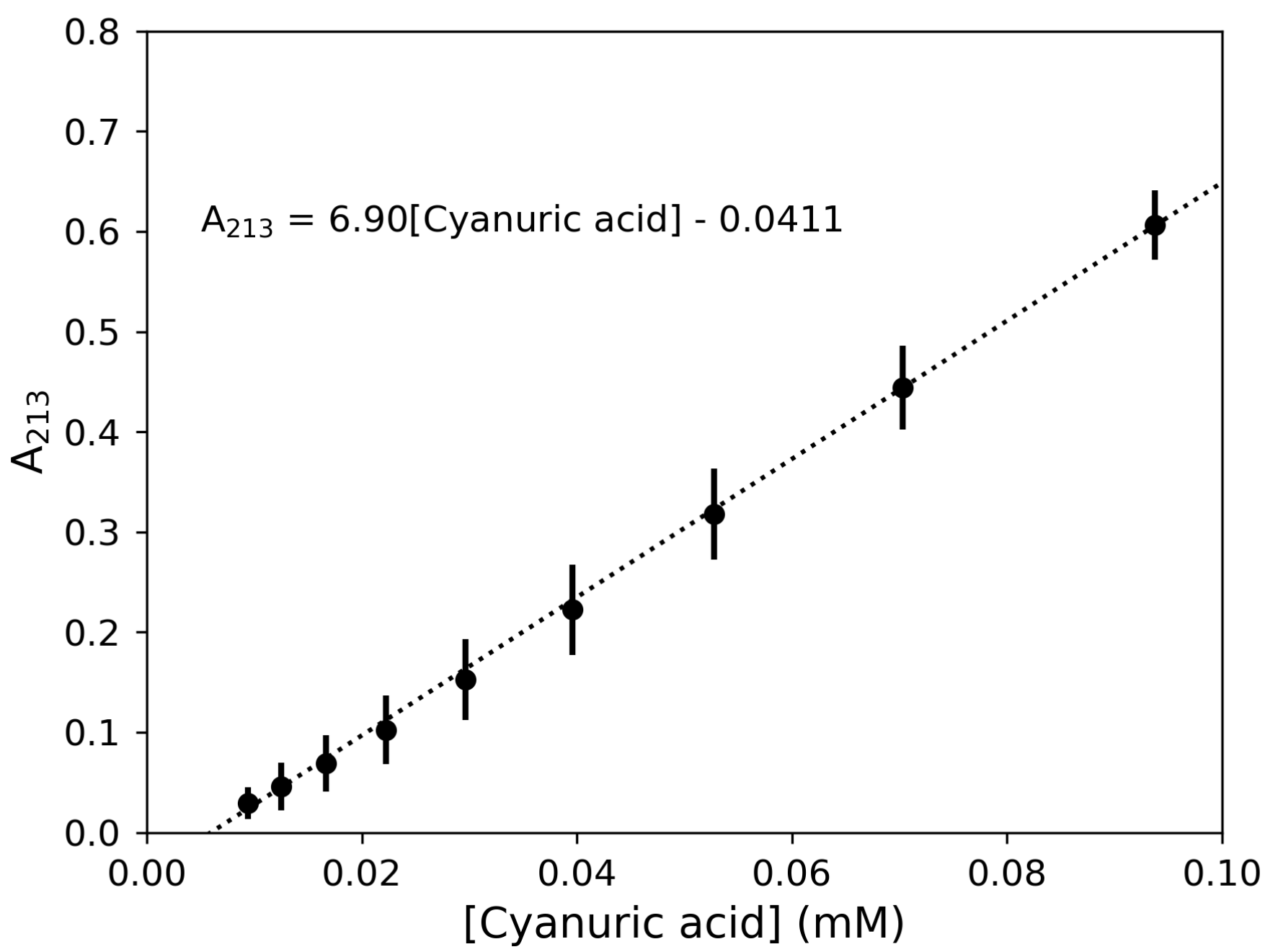

Figure S10: Standard curve for cyanuric acid.

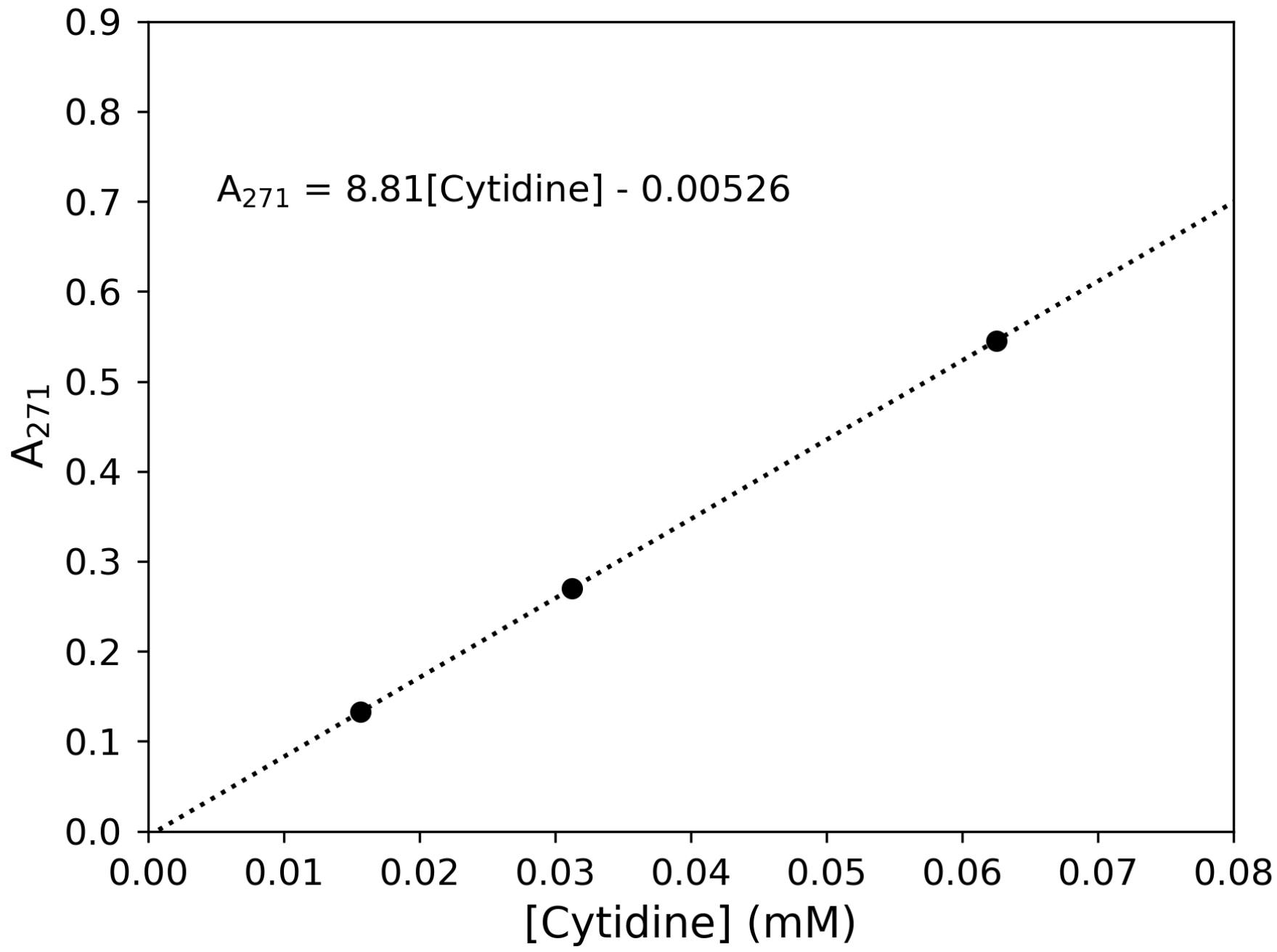

Figure S11: Standard curve for cytidine.

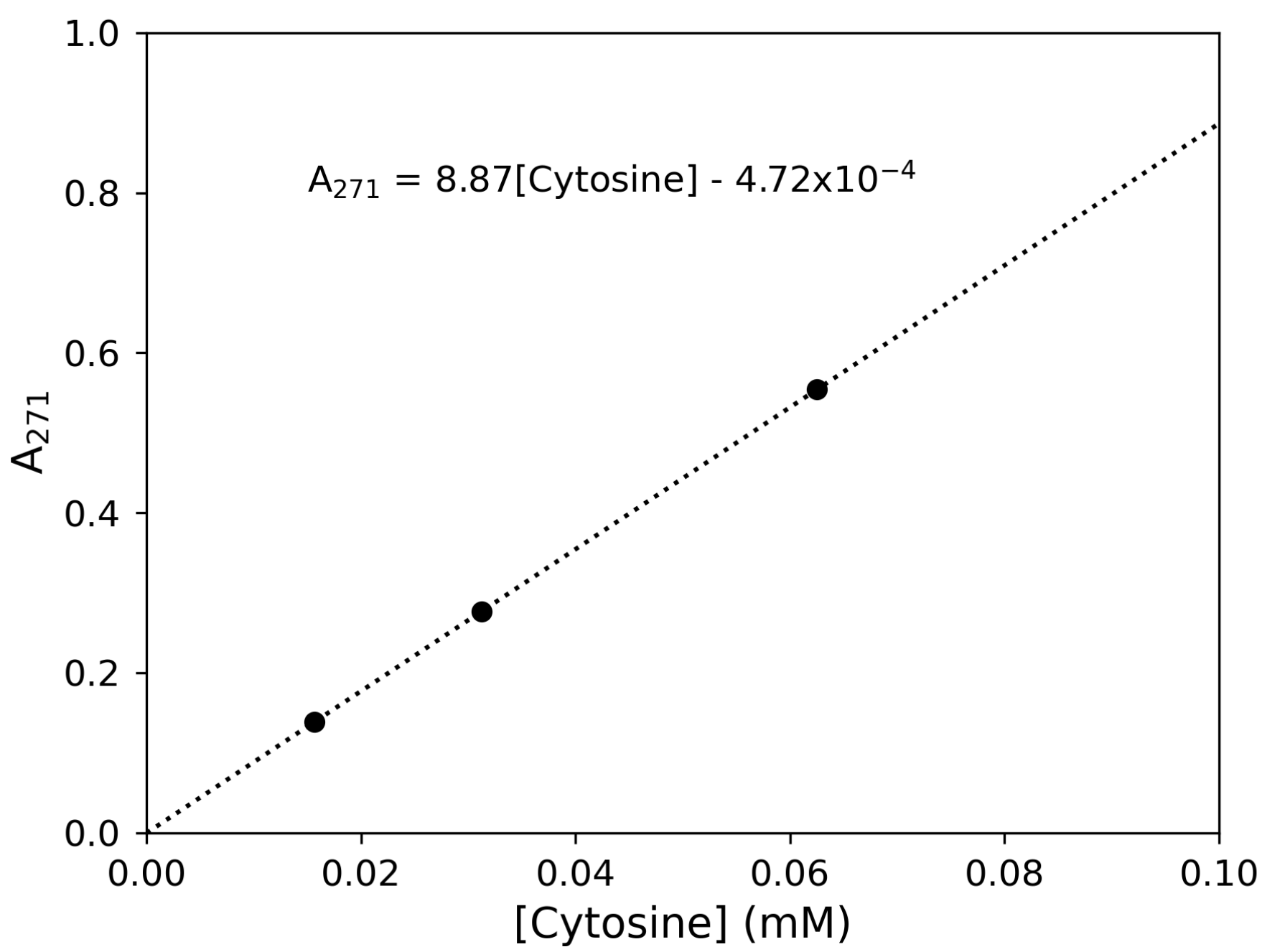

Figure S12: Standard curve for cytosine.

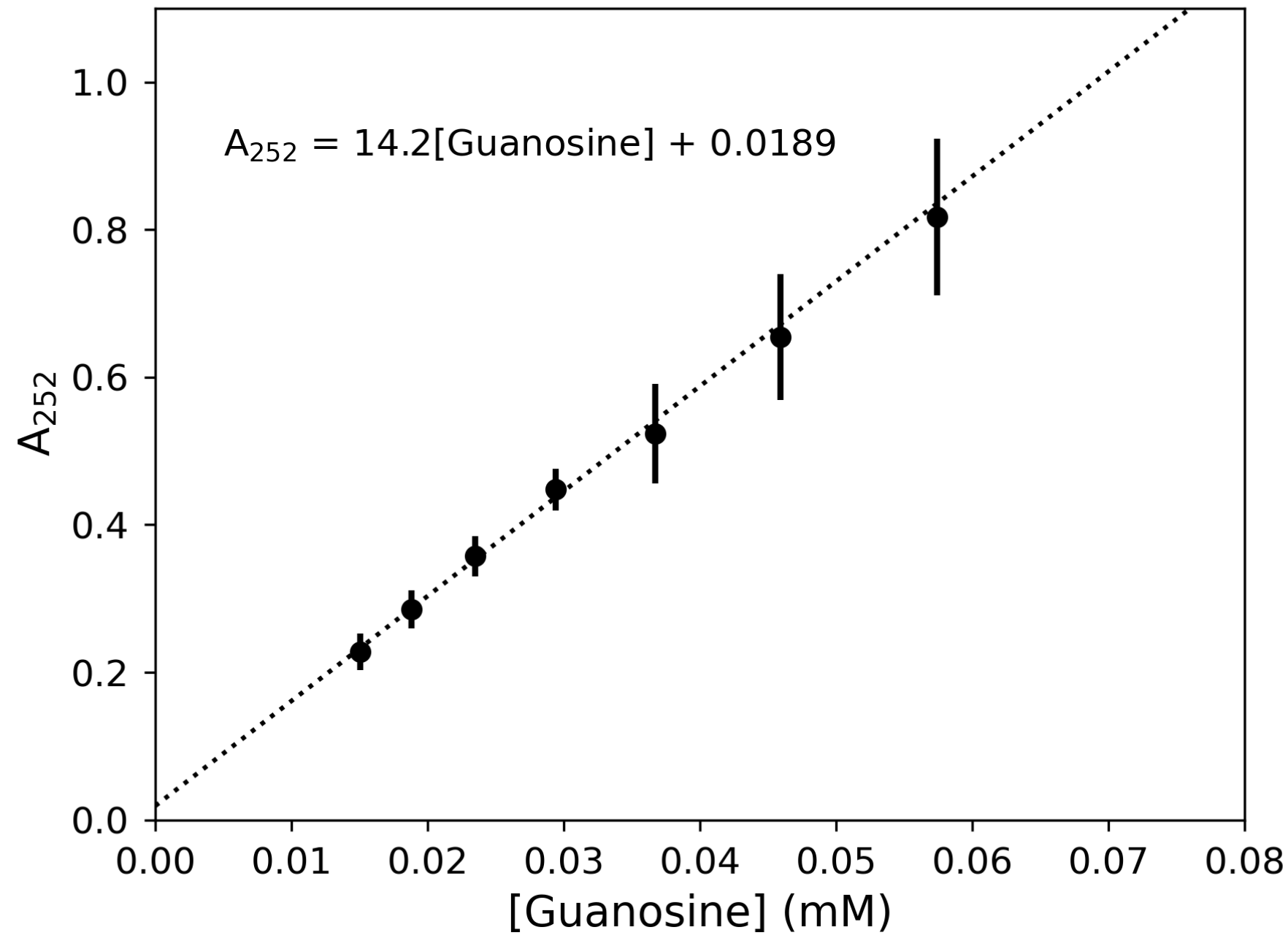

Figure S13: Standard curve for guanosine.

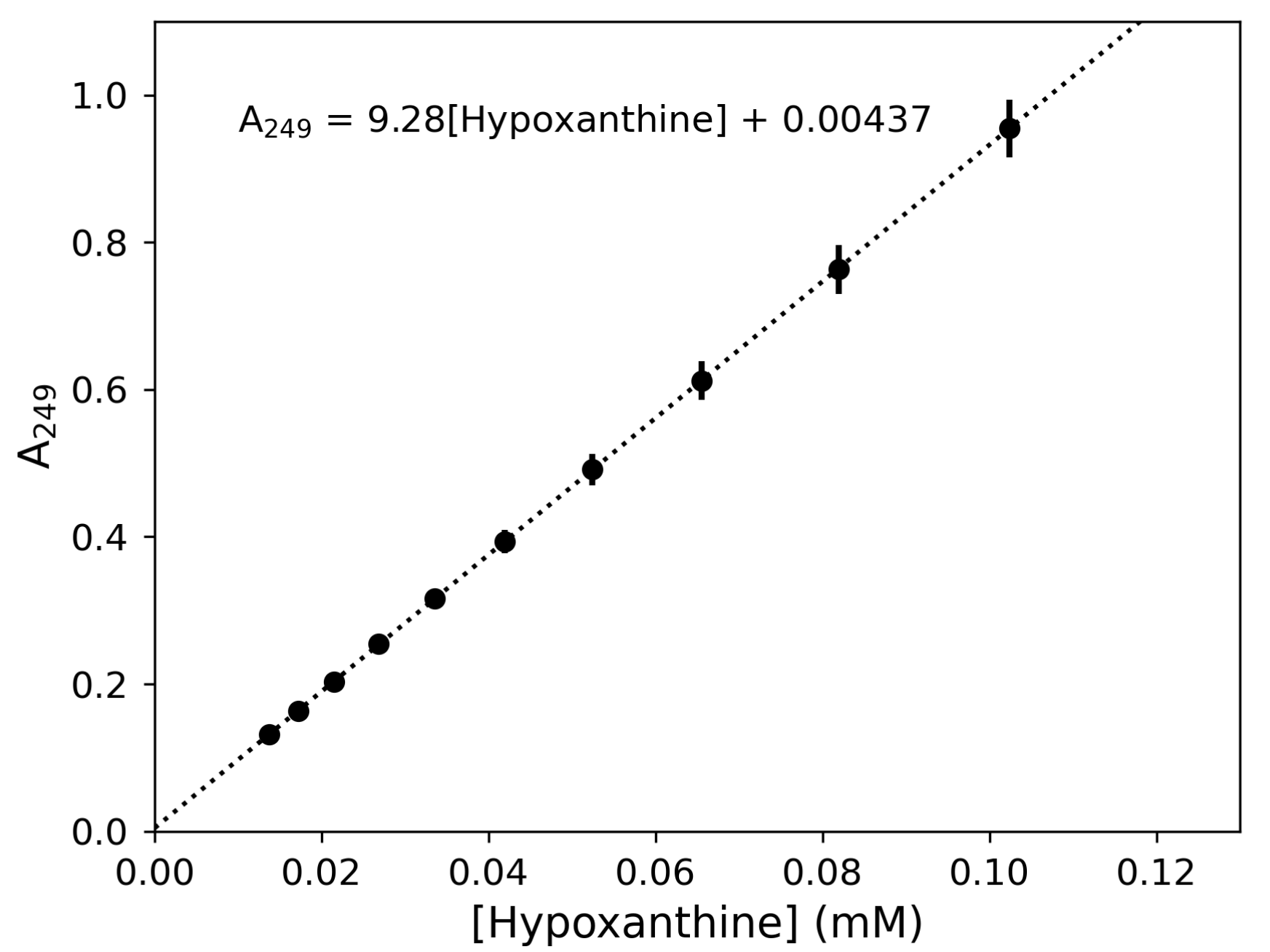

Figure S14: Standard curve for hypoxanthine.

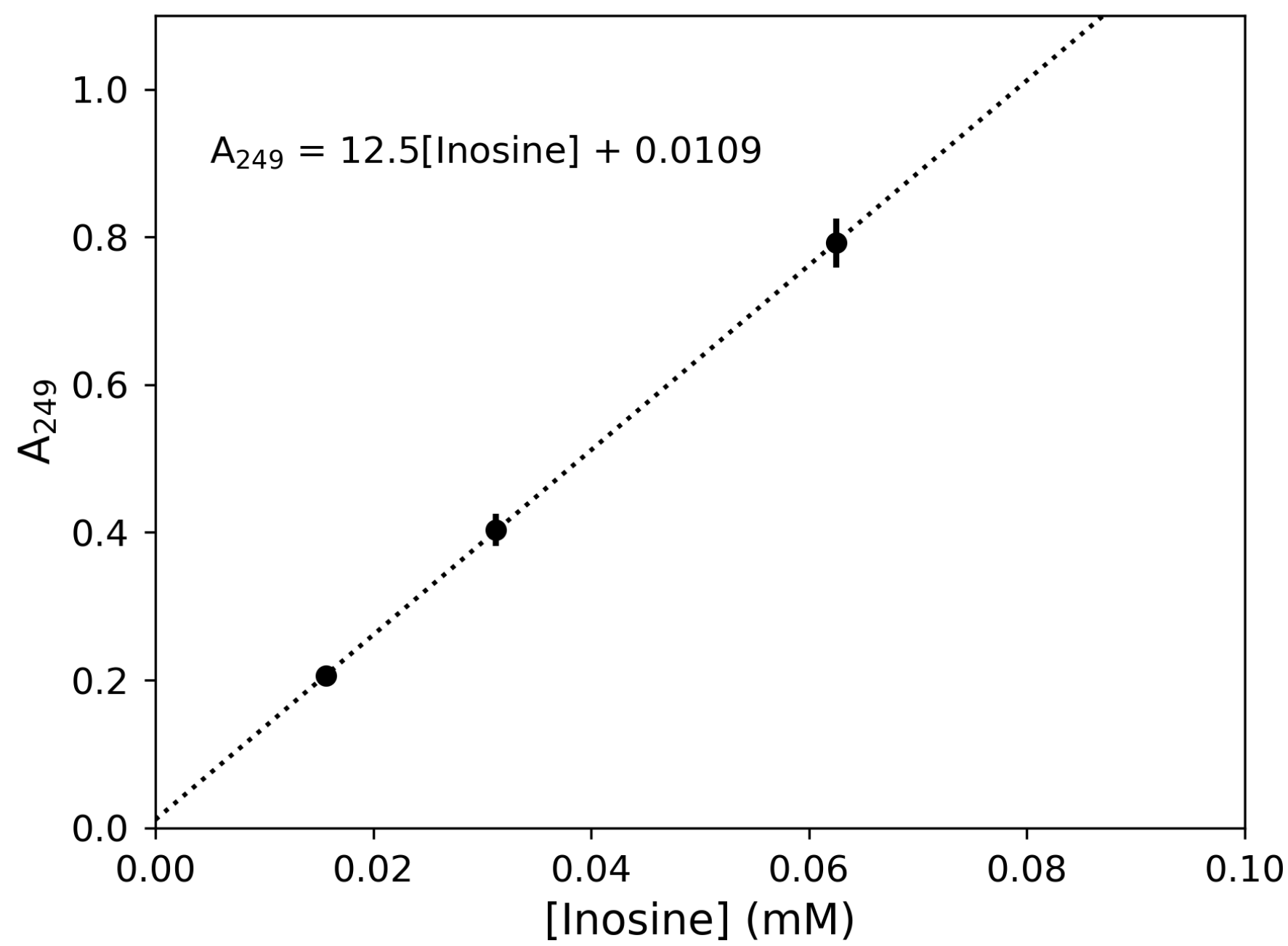

Figure S15: Standard curve for inosine.

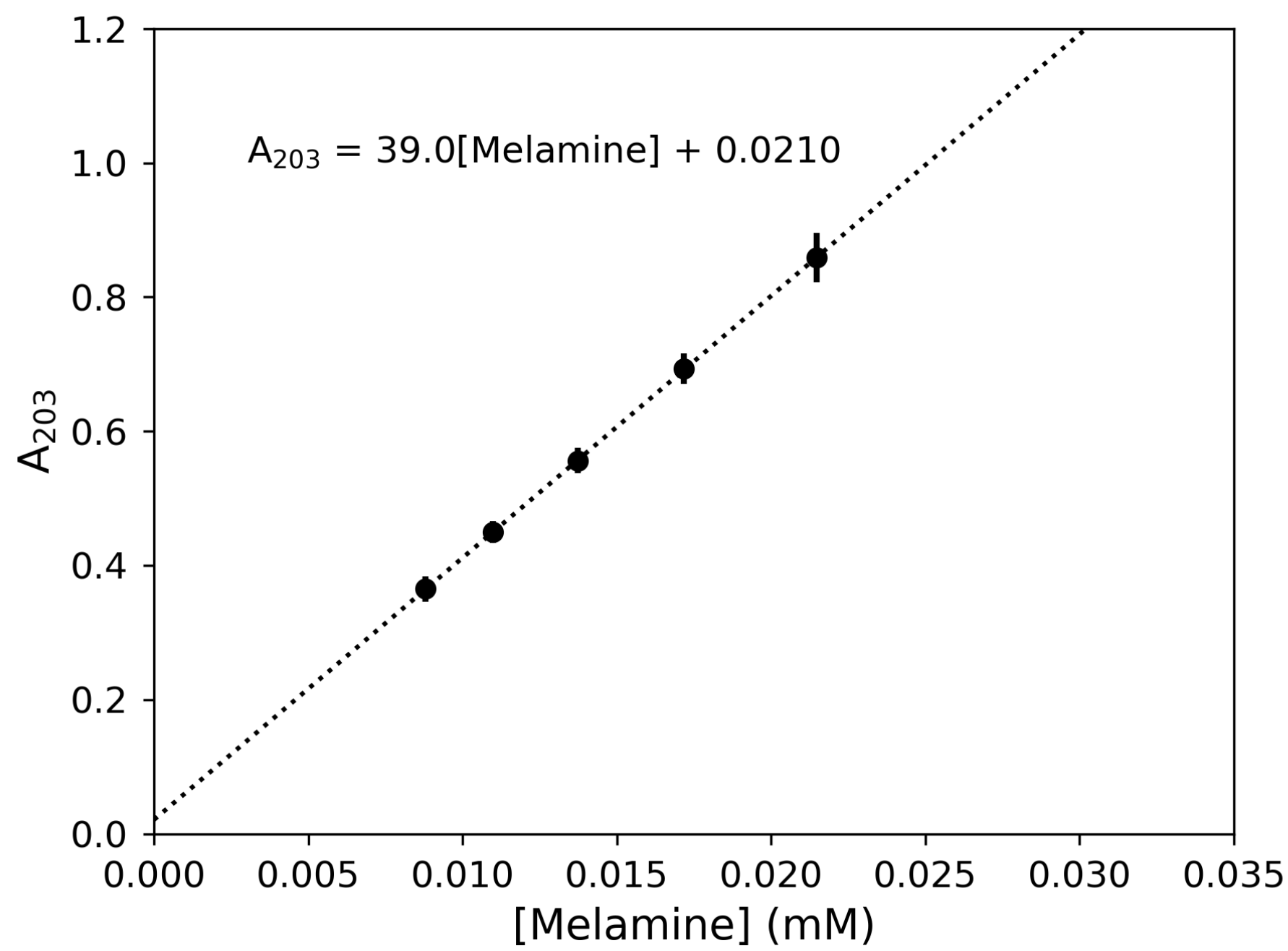

Figure S16: Standard curve for melamine.

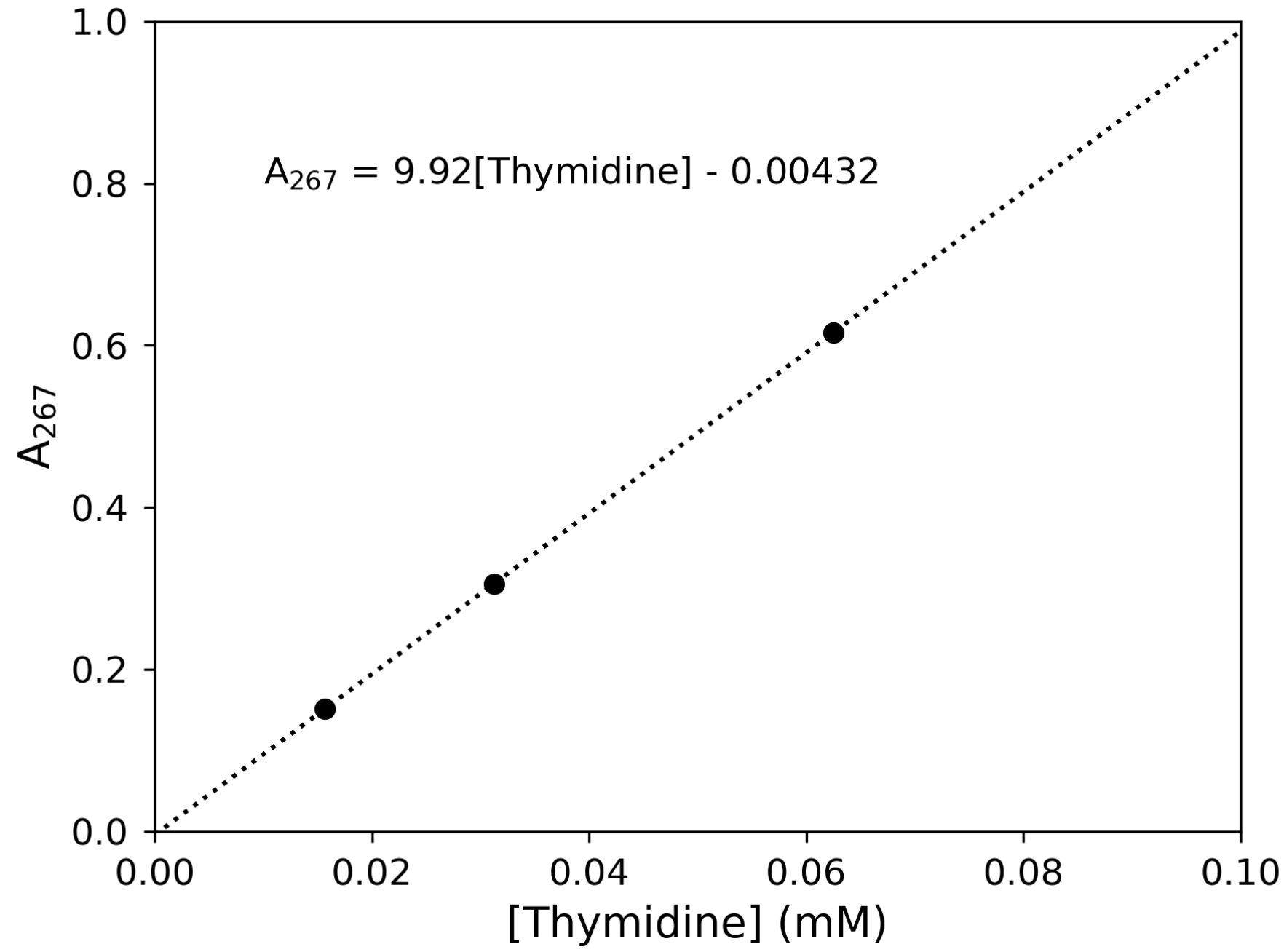

Figure S17: Standard curve for thymidine.

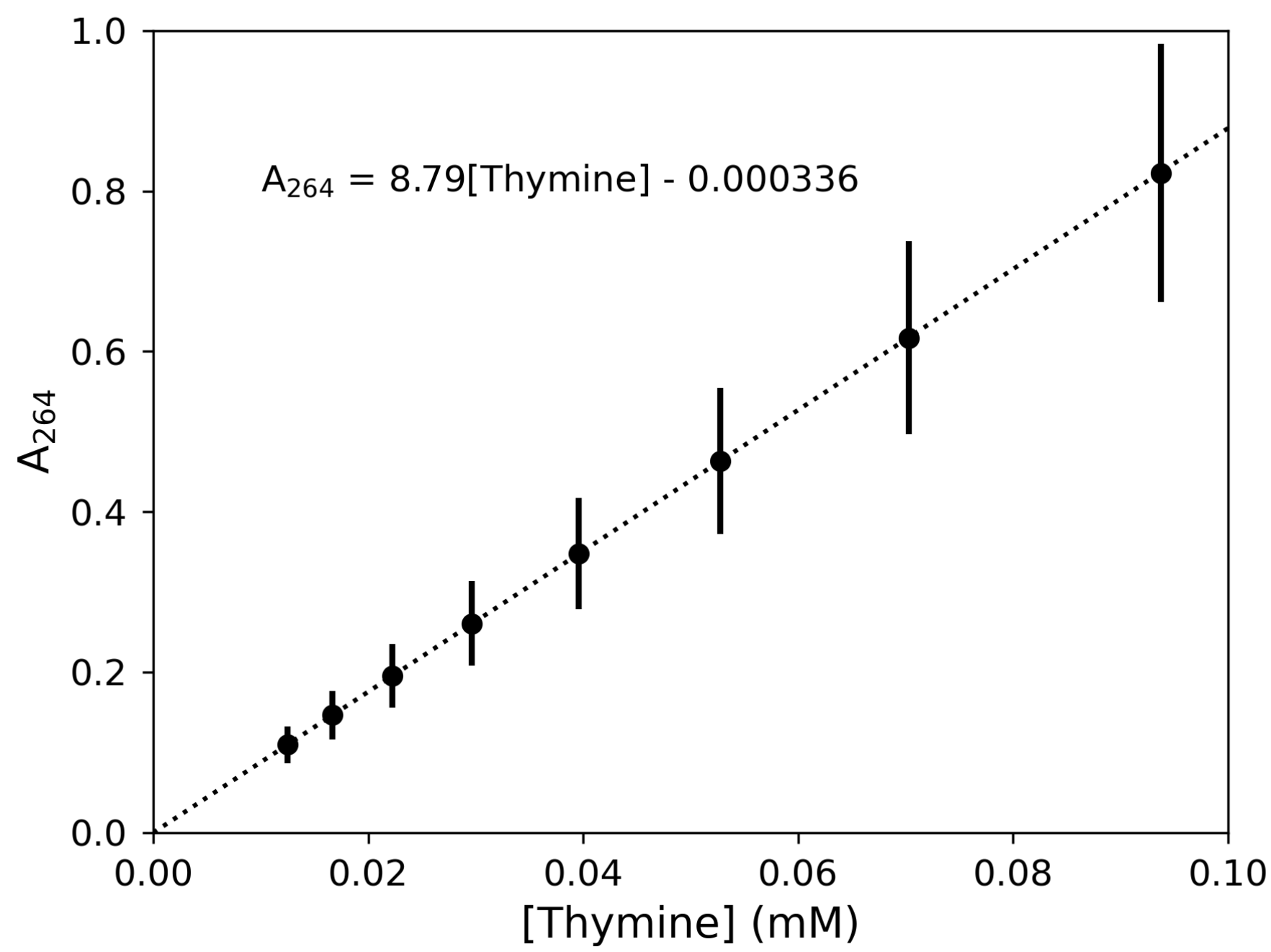

Figure S18: Standard curve for thymine.

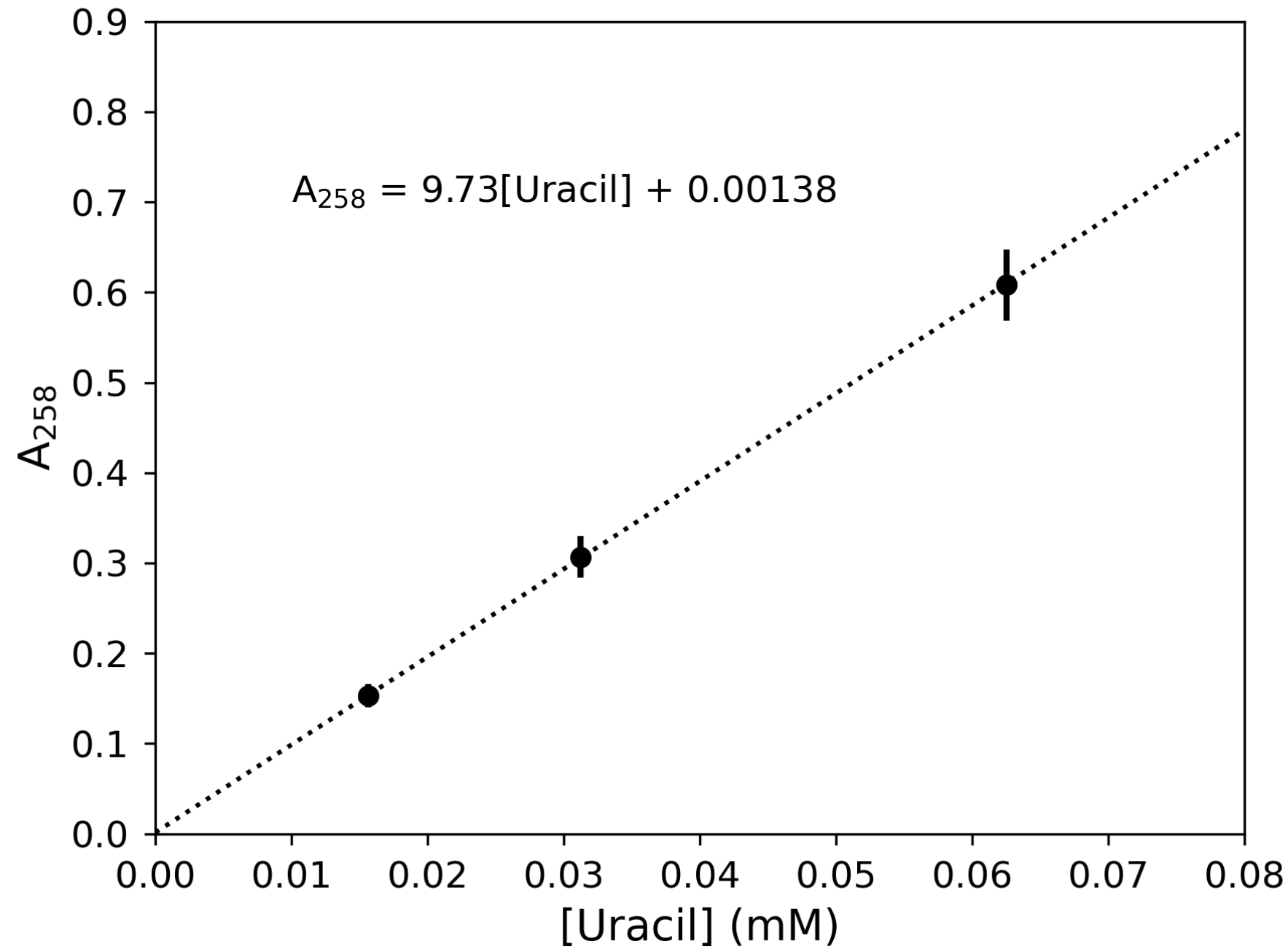

Figure S19: Standard curve for uracil.

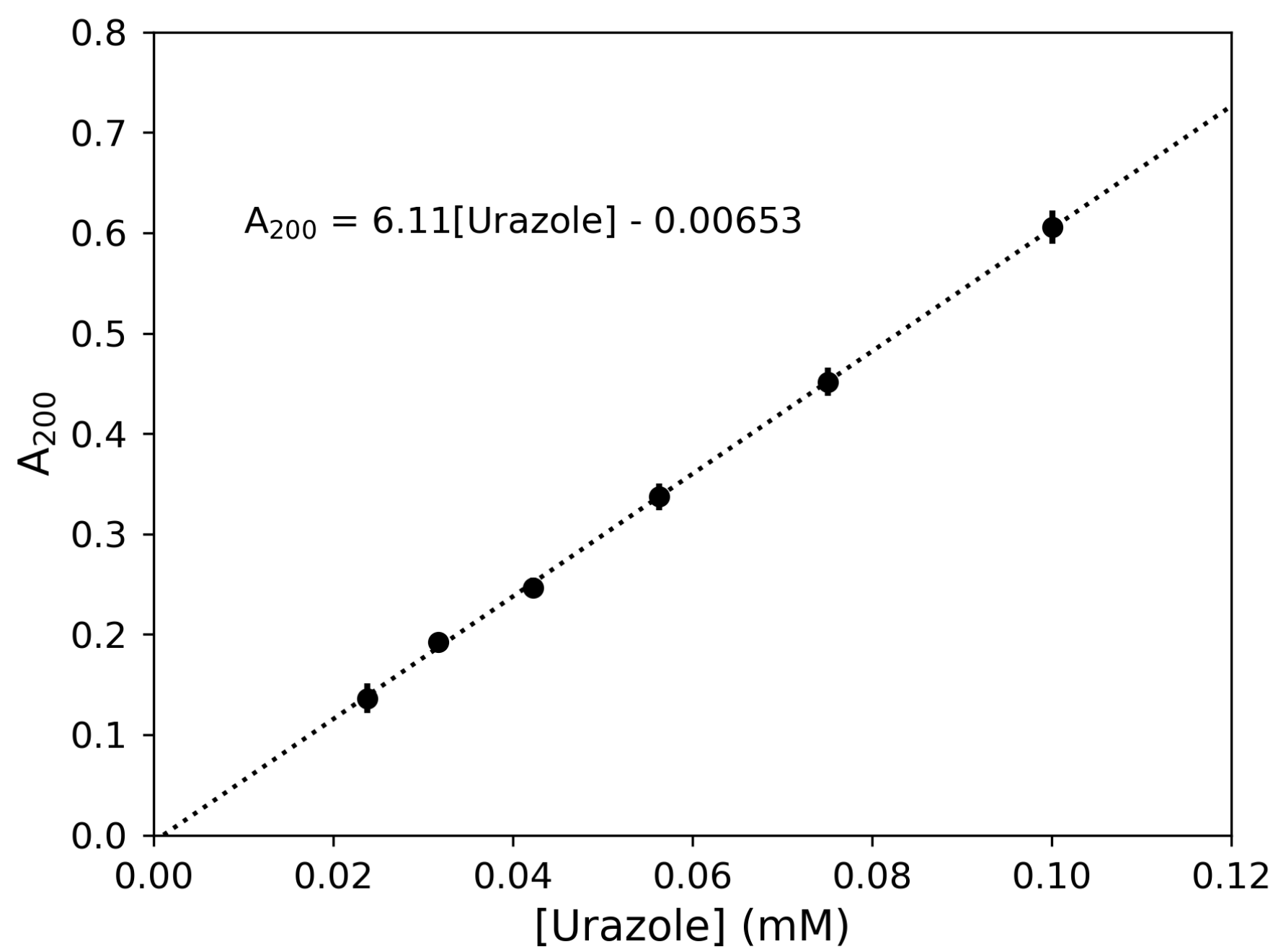

Figure S20: Standard curve for urazole.

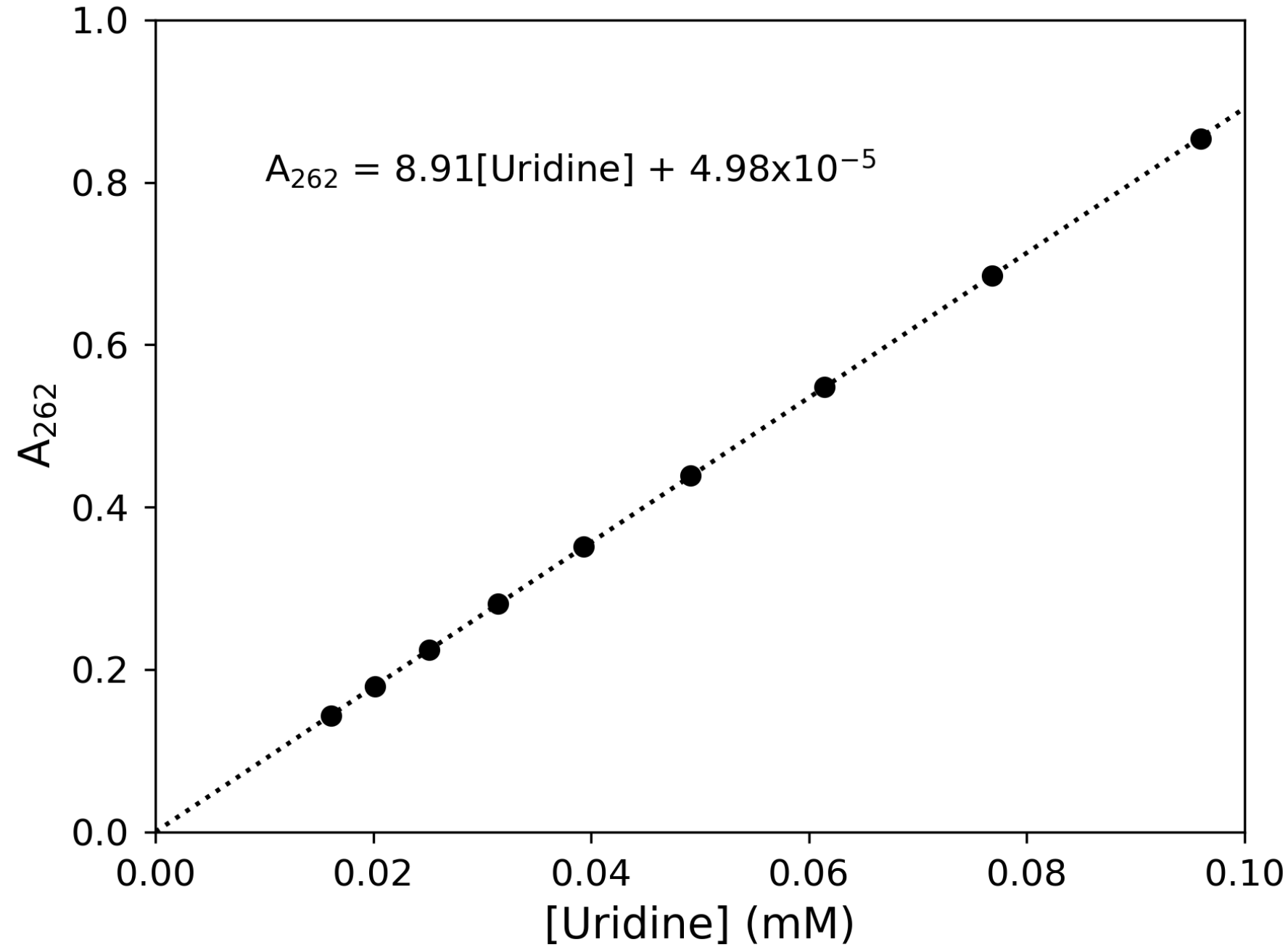

Figure S21: Standard curve for uridine.

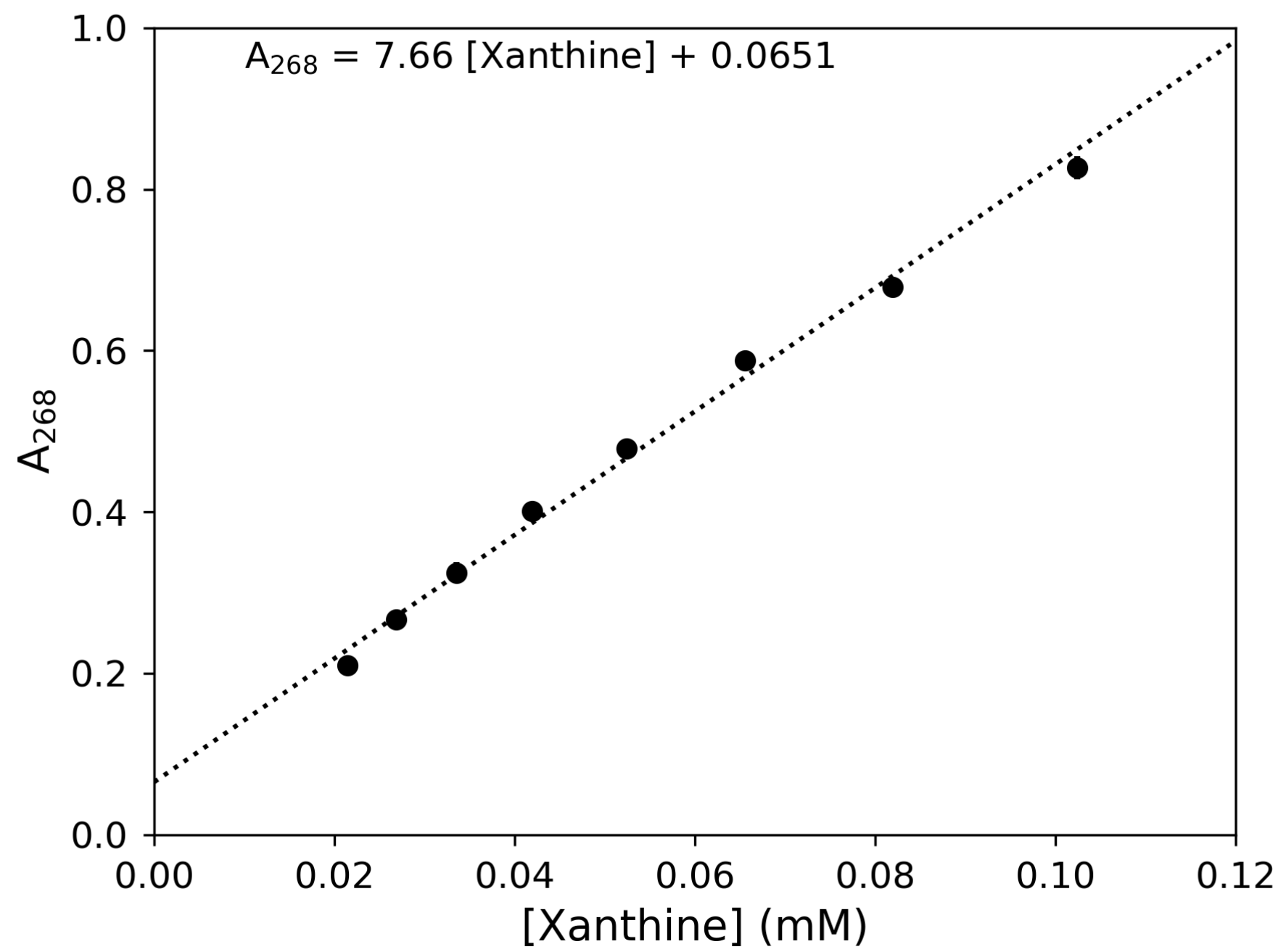

Figure S22: Standard curve for xanthine.

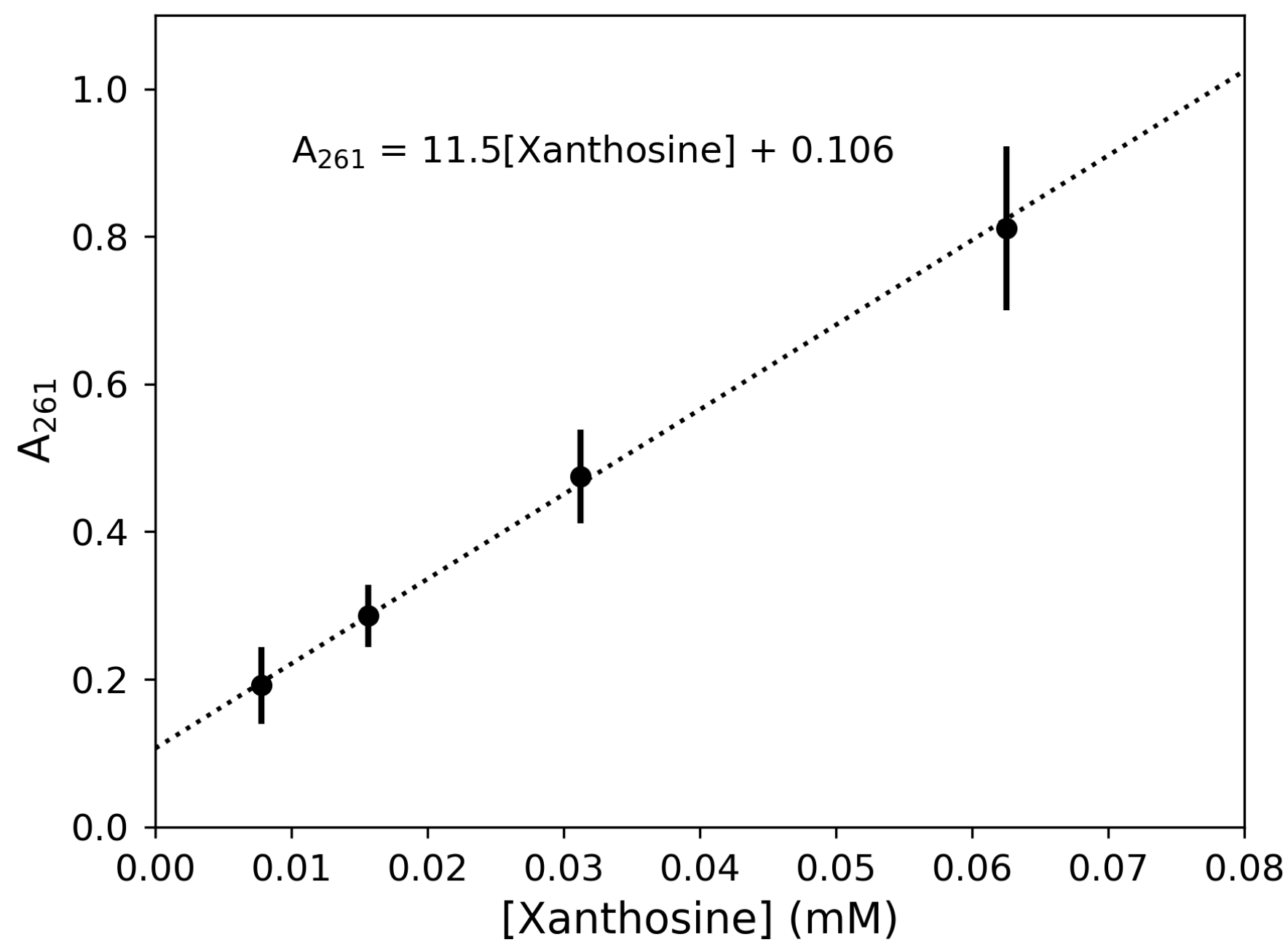

Figure S23: Standard curve for xanthosine.

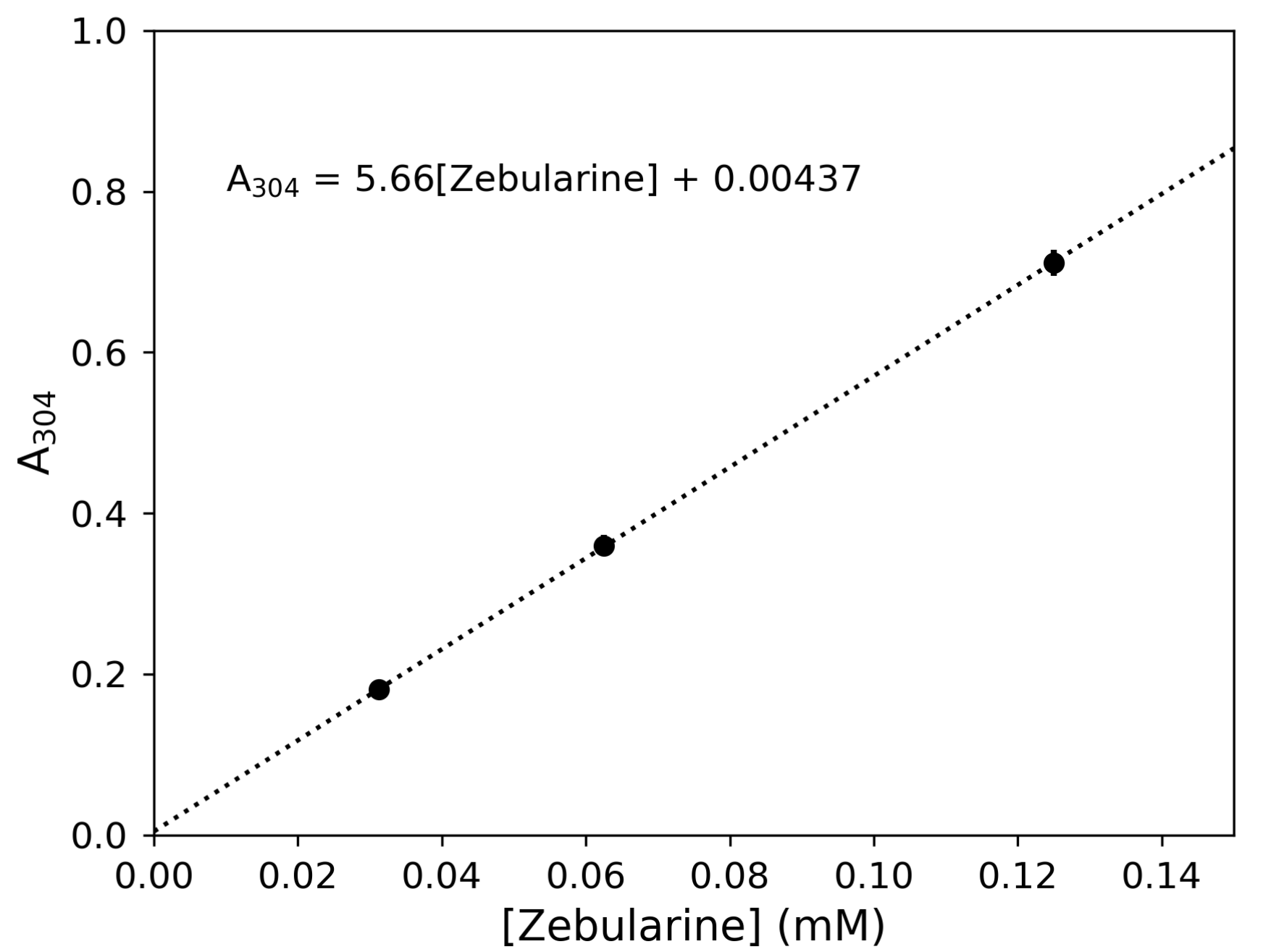

Figure S24: Standard curve for zebularine.

III. UV-Vis spectra and kinetics plots

Figures S25-S50 show all UV-Vis spectra throughout irradiation for each experiment for all molecules. The absorbance at the peak wavelength was converted to concentration at each timepoint using the appropriate standard curve (see SI section II). Then, plots of ln(concentration) vs. time were fit with a linear trendline. In the event that non-linear behavior was observed at later timepoints in the irradiation, we only fit the initial linear portion of the data to calculate the rate constants. The criteria for truncating a data set due to non-linearities is as follows: the first four timepoints of the data set were used to calculate the rate constant and uncertainty due to the goodness of fit for each experiment in the triplicate set. Next, each timepoint was subsequently added and fit with a trendline to determine the new rate constant and associated uncertainty. If the percent error (uncertainty/rate constant x 100%) increased in all three replicates by adding an additional timepoint to the dataset, the data was truncated to include only the previous timepoints. With this criteria, data for the following molecules were truncated and rate constants were reported accordingly: adenine (t<28 min), hypoxanthine (t<22 min), melamine (t<37 min), thymidine (t<10 min), thymine (t<13 min), uracil (t<2.2 min), urazole (t<32 min). Xanthine exhibited non-linear behavior and a shifting peak absorbance during the course of the irradiation. While our truncation criteria was not met for xanthine, we instead truncated the data at t<3.1 min, when the peak absorbance shifted from the original peak at 268 nm, since this peak shifting could suggest the formation of side products with absorption features that could interfere with the calculation of the rate constants.

Similarly, we irradiated 5-methylcytidine and 5-methylcytosine but observed significant shifting of absorption peaks throughout the irradiation. The extent of this peak shifting prevented the calculation of rate constants for these molecules. Their absorption spectra throughout irradiation of 5-methylcytidine and 5-methylcytosine are shown in Figures S49 and S50, respectively.

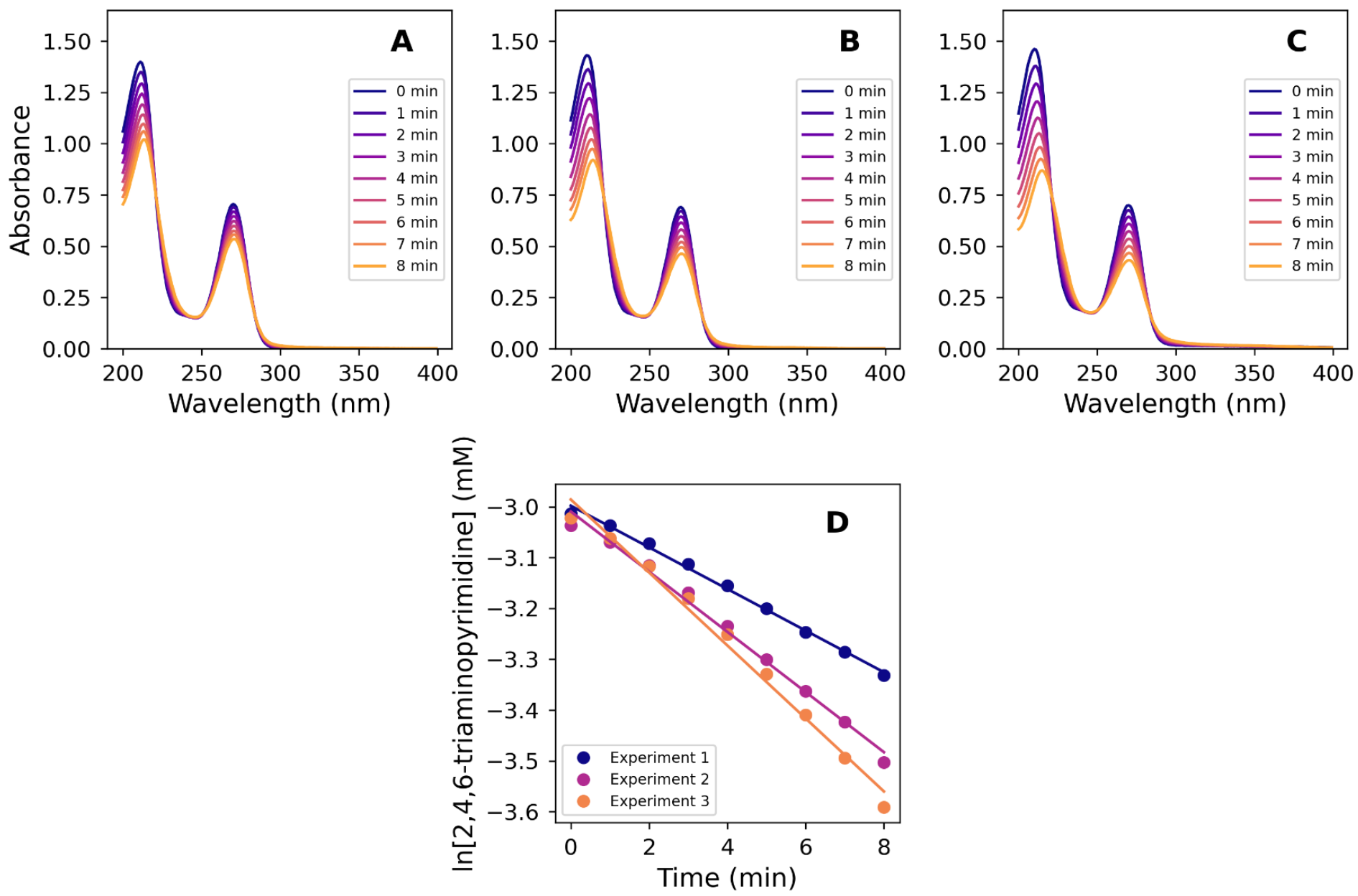

Figure S25: UV-Vis spectra of 2,4,6-triaminopyrimidine throughout irradiation for individual experiments (A-C) and kinetics plot of ln(concentration) vs. time to determine the first-order rate constants (D).

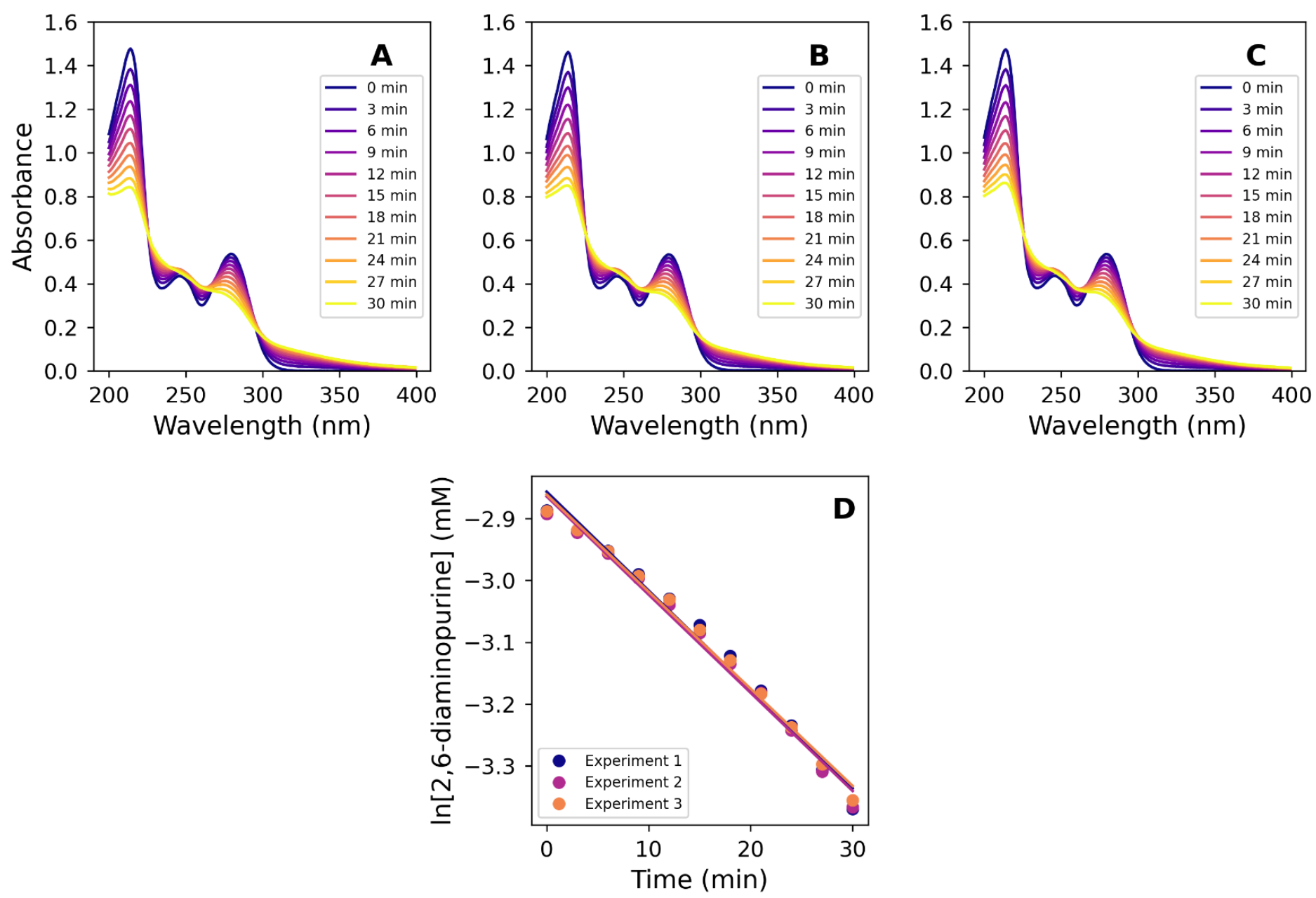

Figure S26: UV-Vis spectra of 2,6-diaminopurine throughout irradiation for individual experiments (A-C) and kinetics plot of ln(concentration) vs. time to determine first-order rate constants (D).

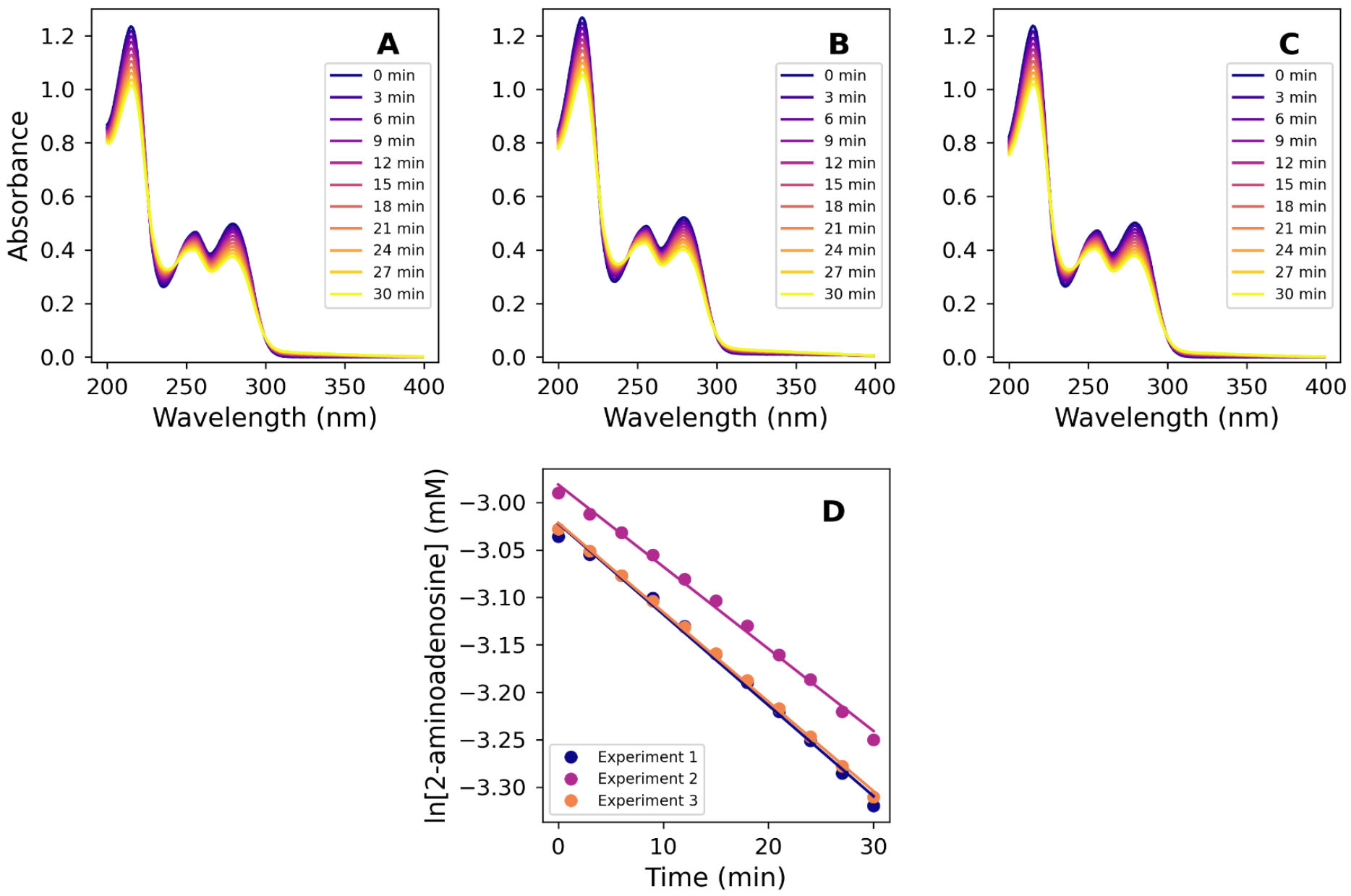

Figure S27: UV-Vis spectra of 2-aminoadenosine throughout irradiation for individual experiments (A-C) and kinetics plot of ln(concentration) vs. time to determine first-order rate constants (D).

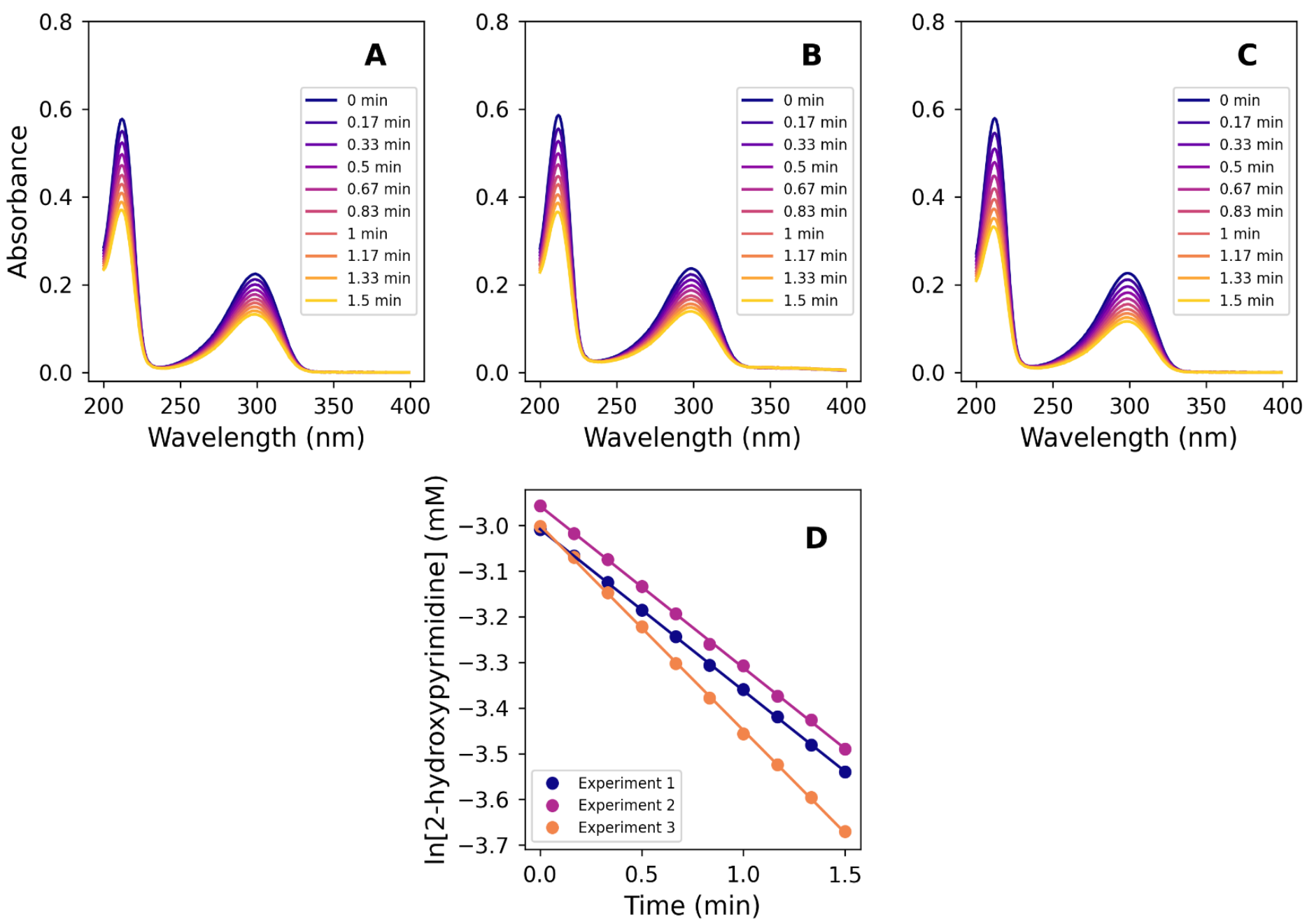

Figure S28: UV-Vis spectra of 2-hydroxypyrimidine throughout irradiation for individual experiments (A-C) and kinetics plot of ln(concentration) vs. time to determine first-order rate constants (D).

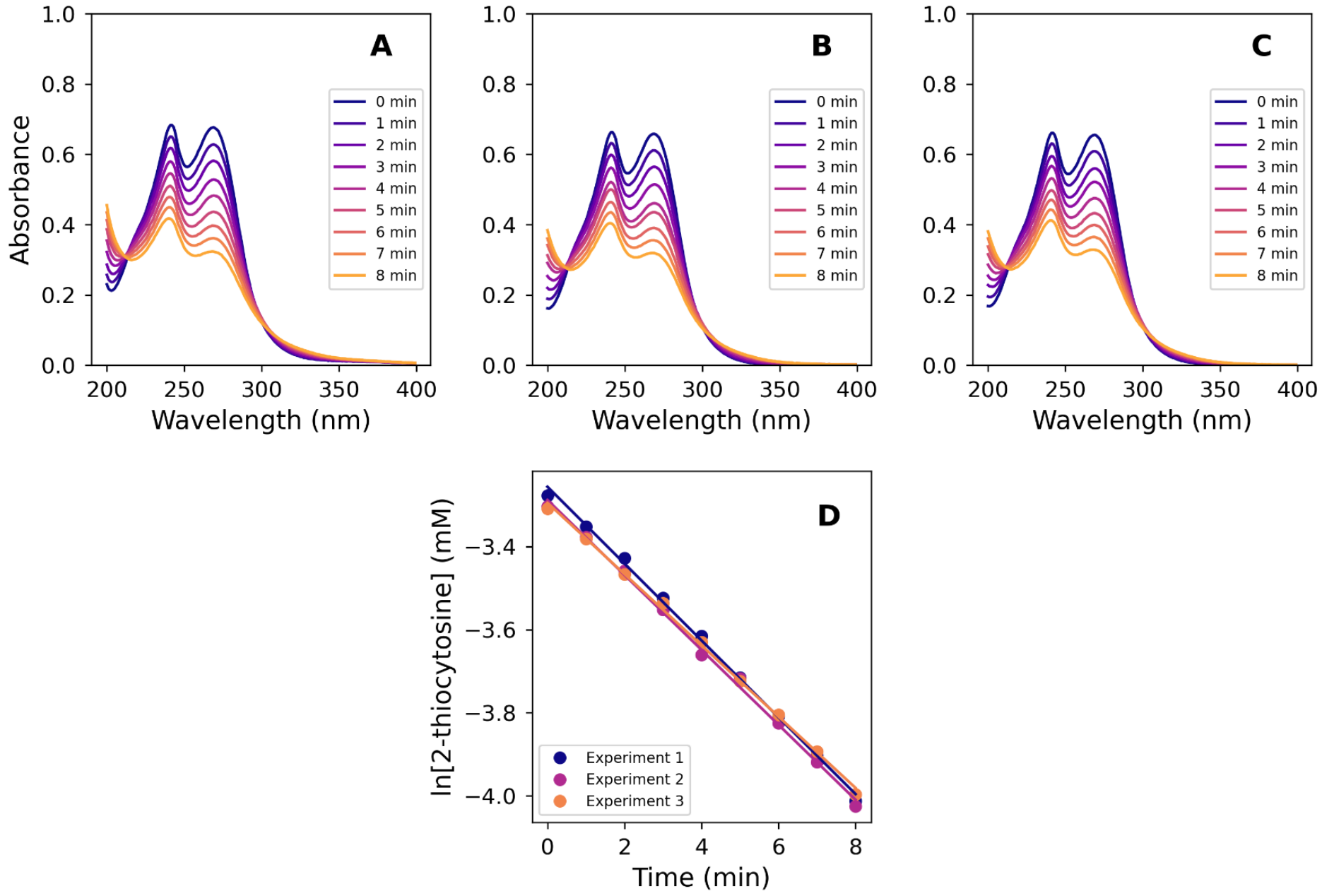

Figure S29: UV-Vis spectra of 2-thiocytosine throughout irradiation for individual experiments (A-C) and kinetics plot of ln(concentration) vs. time to determine first-order rate constants (D).

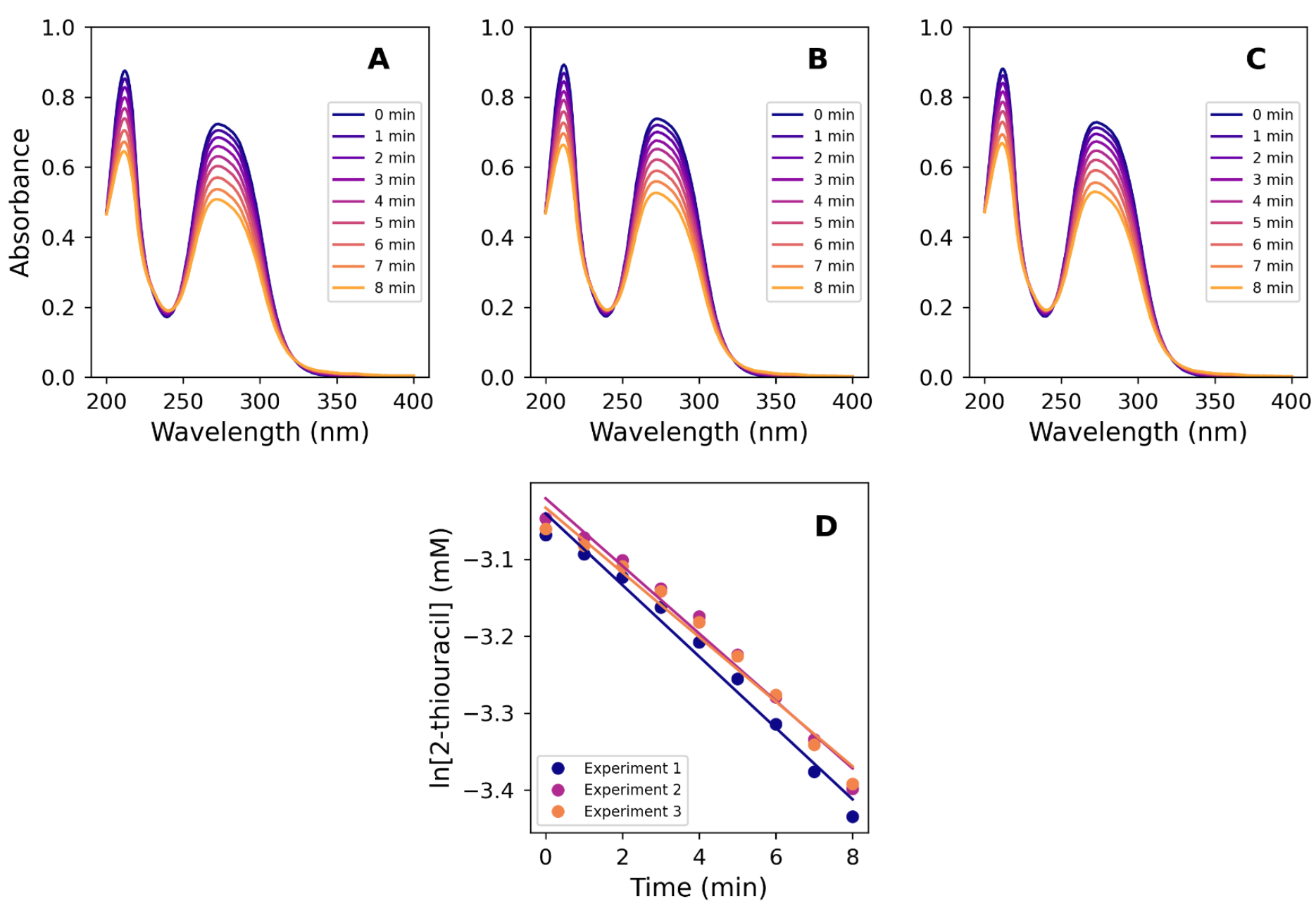

Figure S30: UV-Vis spectra of 2-thiouracil throughout irradiation for individual experiments (A-C) and kinetics plot of ln(concentration) vs. time to determine first-order rate constants (D).

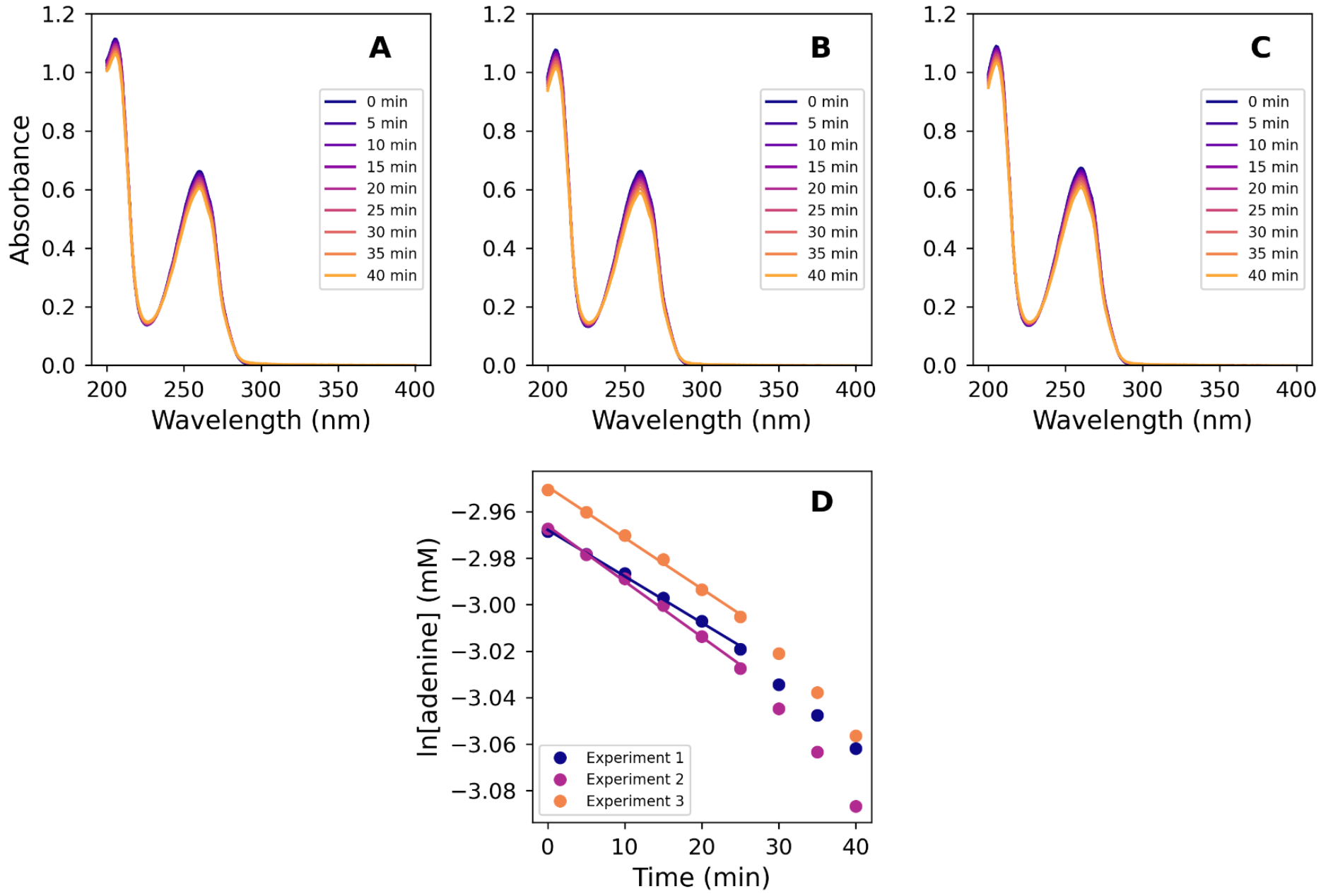

Figure S31: UV-Vis spectra of adenine throughout irradiation for individual experiments (A-C) and kinetics plot of ln(concentration) vs. time to determine first-order rate constants (D).

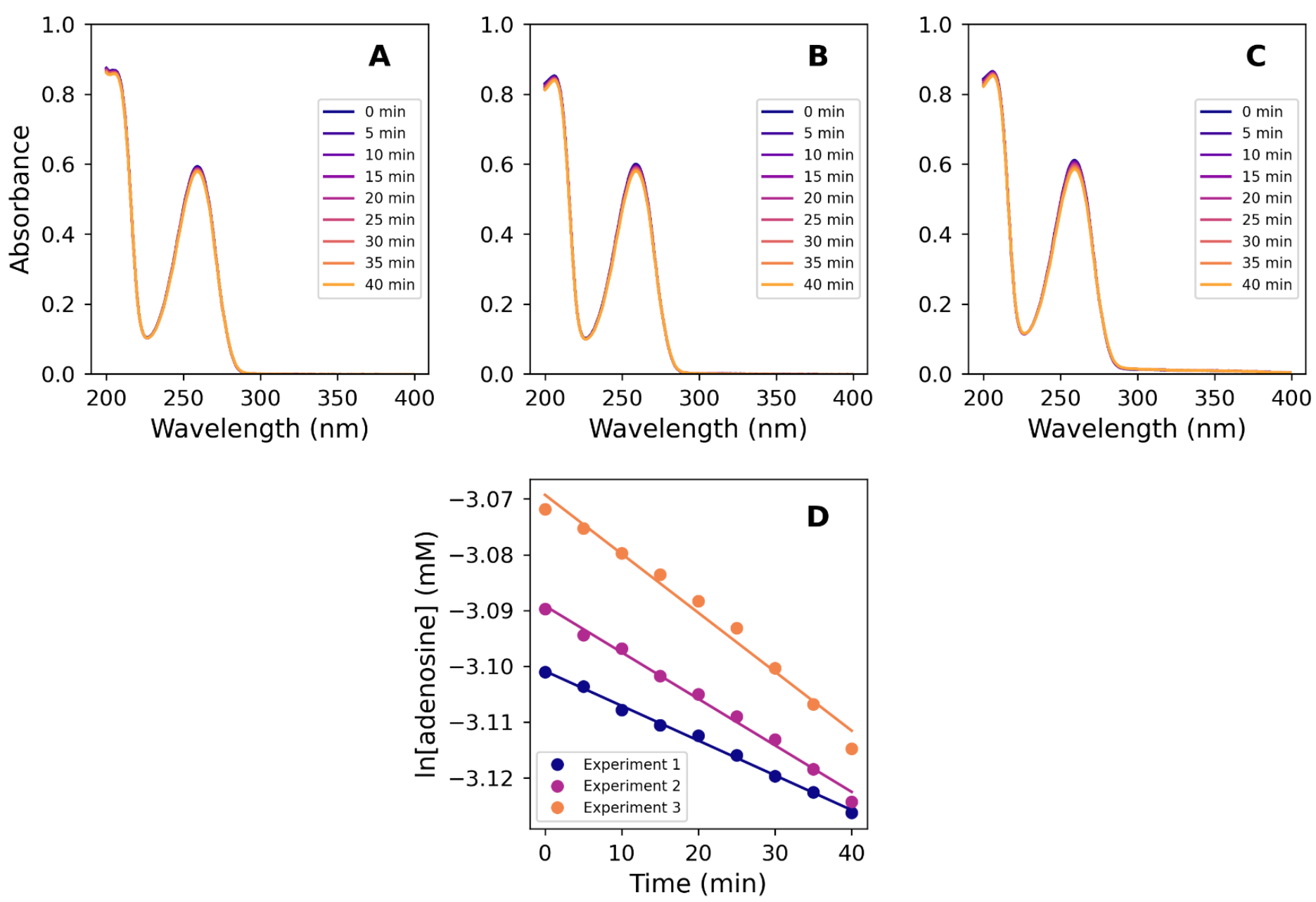

Figure S32: UV-Vis spectra of adenosine throughout irradiation for individual experiments (A-C) and kinetics plot of ln(concentration) vs. time to determine first-order rate constants (D).

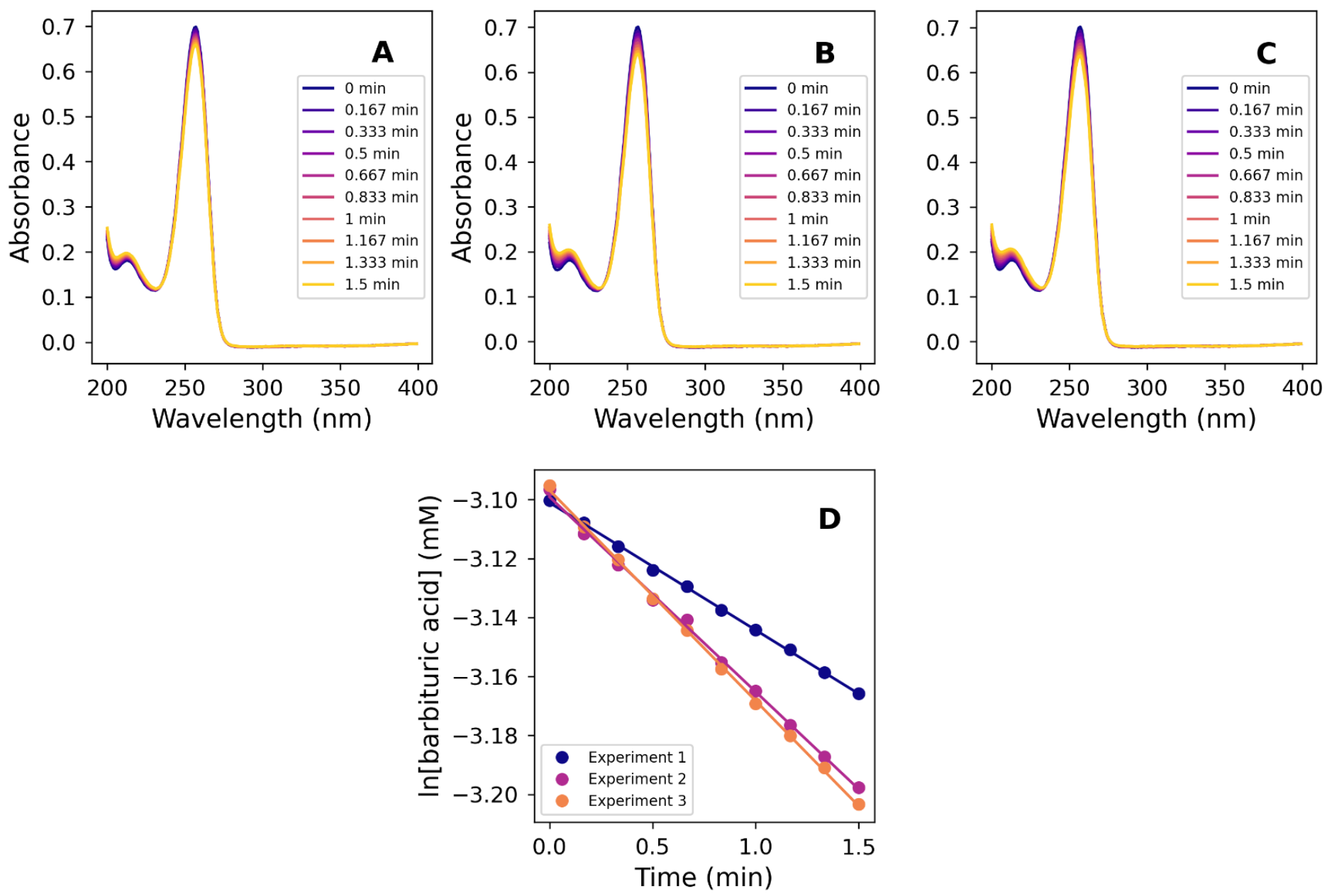

Figure S33: UV-Vis spectra of barbituric acid throughout irradiation for individual experiments (A-C) and kinetics plot of ln(concentration) vs. time to determine first-order rate constants (D).

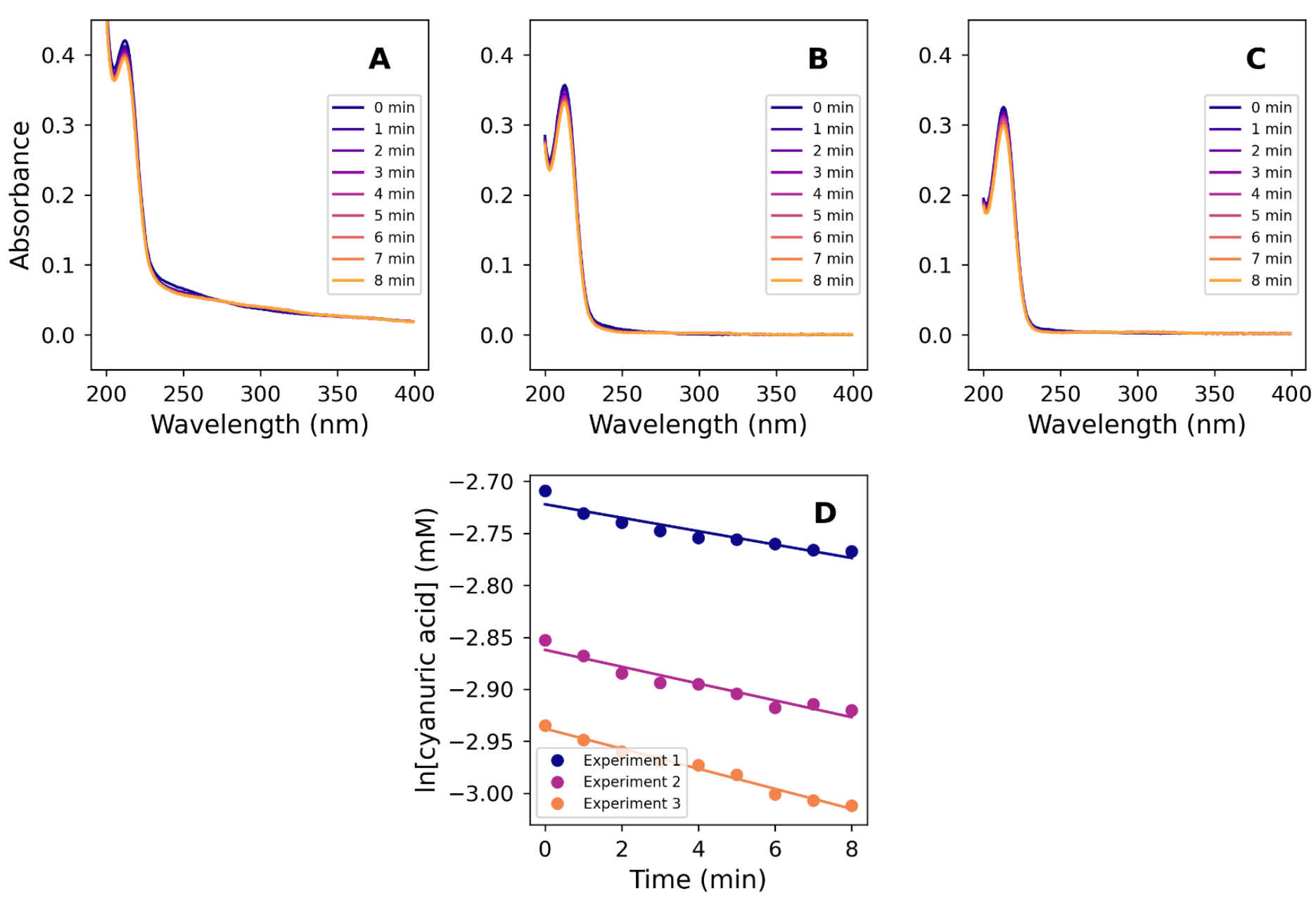

Figure S34: UV-Vis spectra of cyanuric acid (pH 7.30) throughout irradiation for individual experiments (A-C) and kinetics plot of ln(concentration) vs. time to determine first-order rate constants (D).

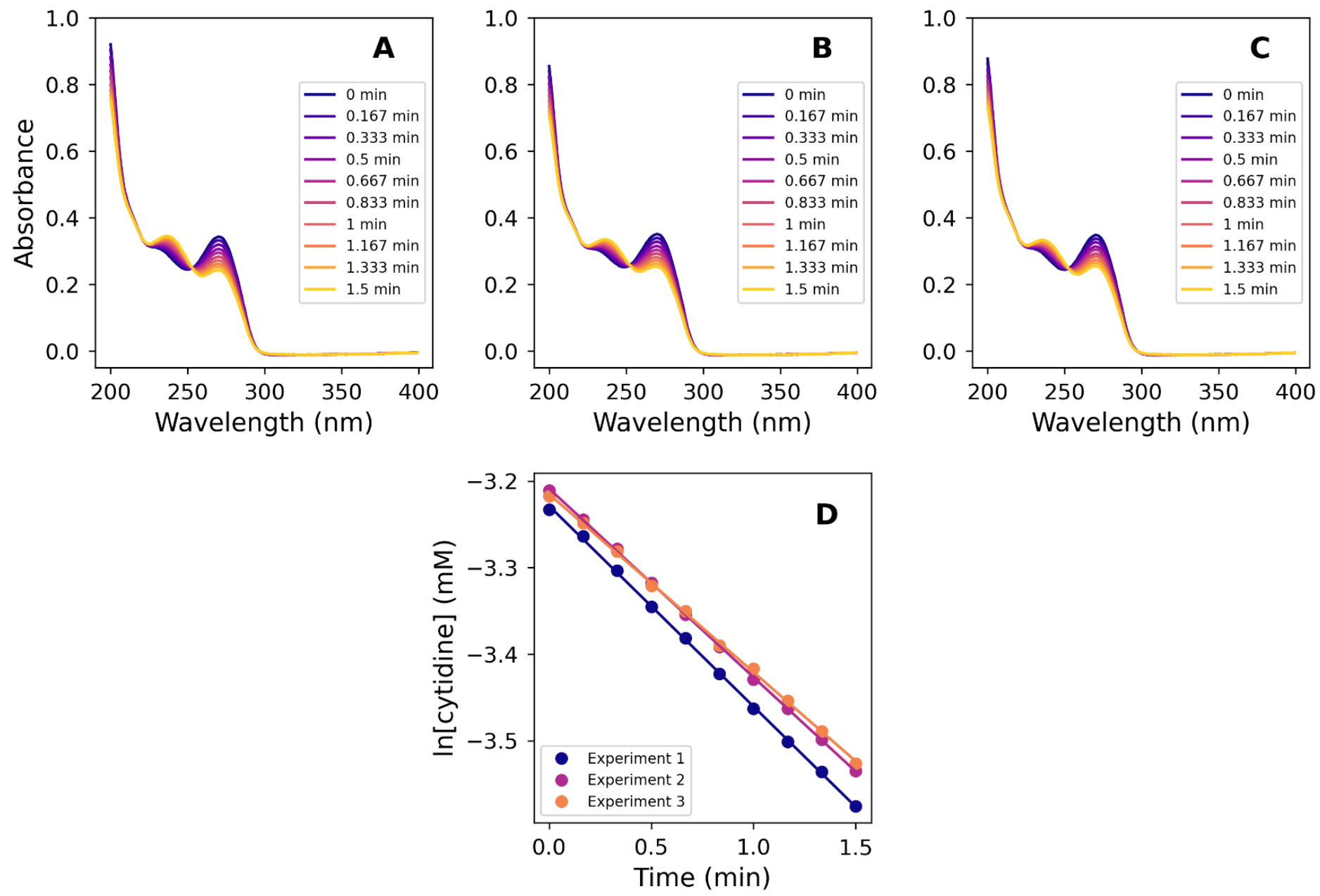

Figure S35: UV-Vis spectra of cytidine throughout irradiation for individual experiments (A-C) and kinetics plot of ln(concentration) vs. time to determine first-order rate constants (D).

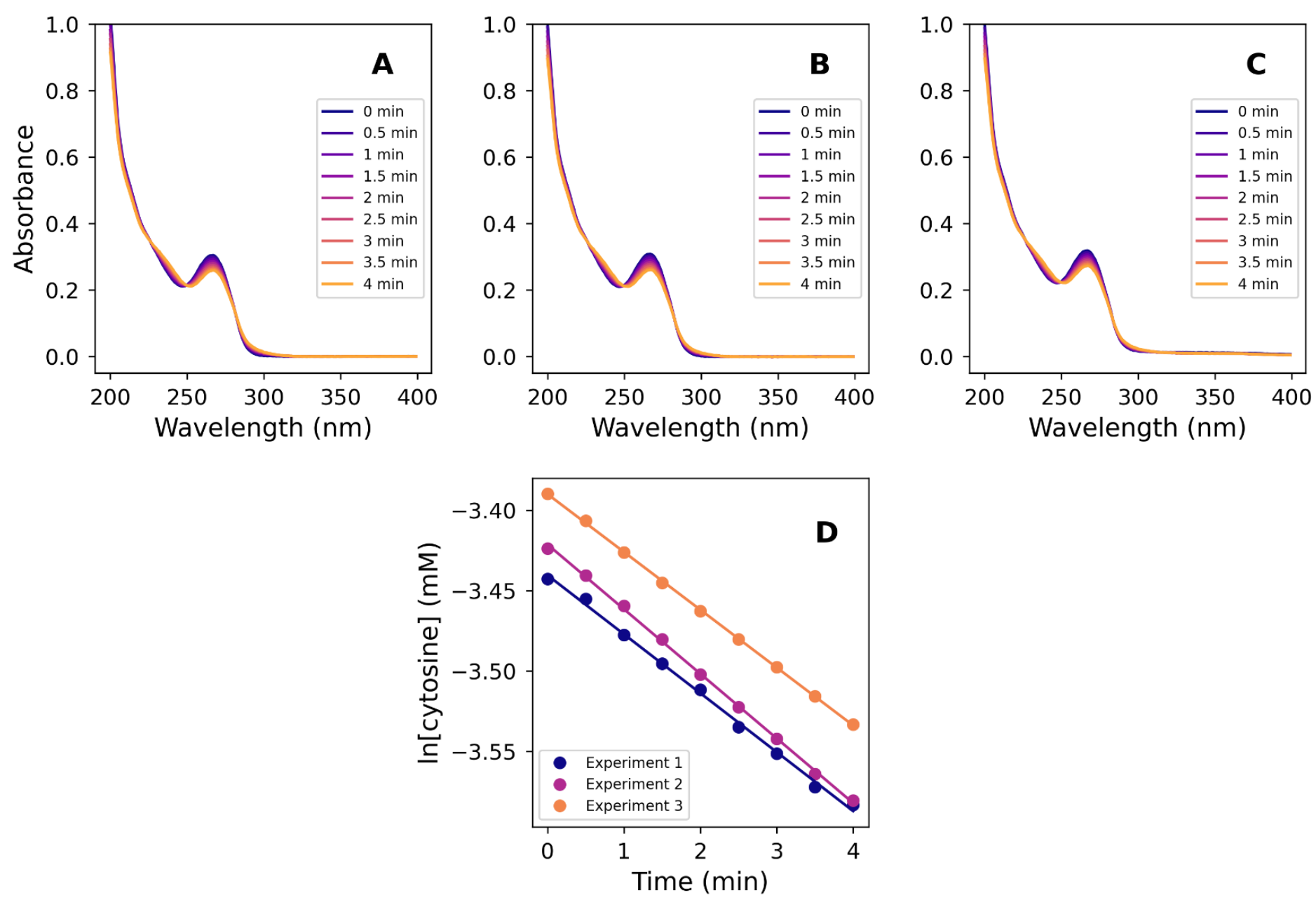

Figure S36: UV-Vis spectra of cytosine throughout irradiation for individual experiments (A-C) and kinetics plot of ln(concentration) vs. time to determine first-order rate constants (D).

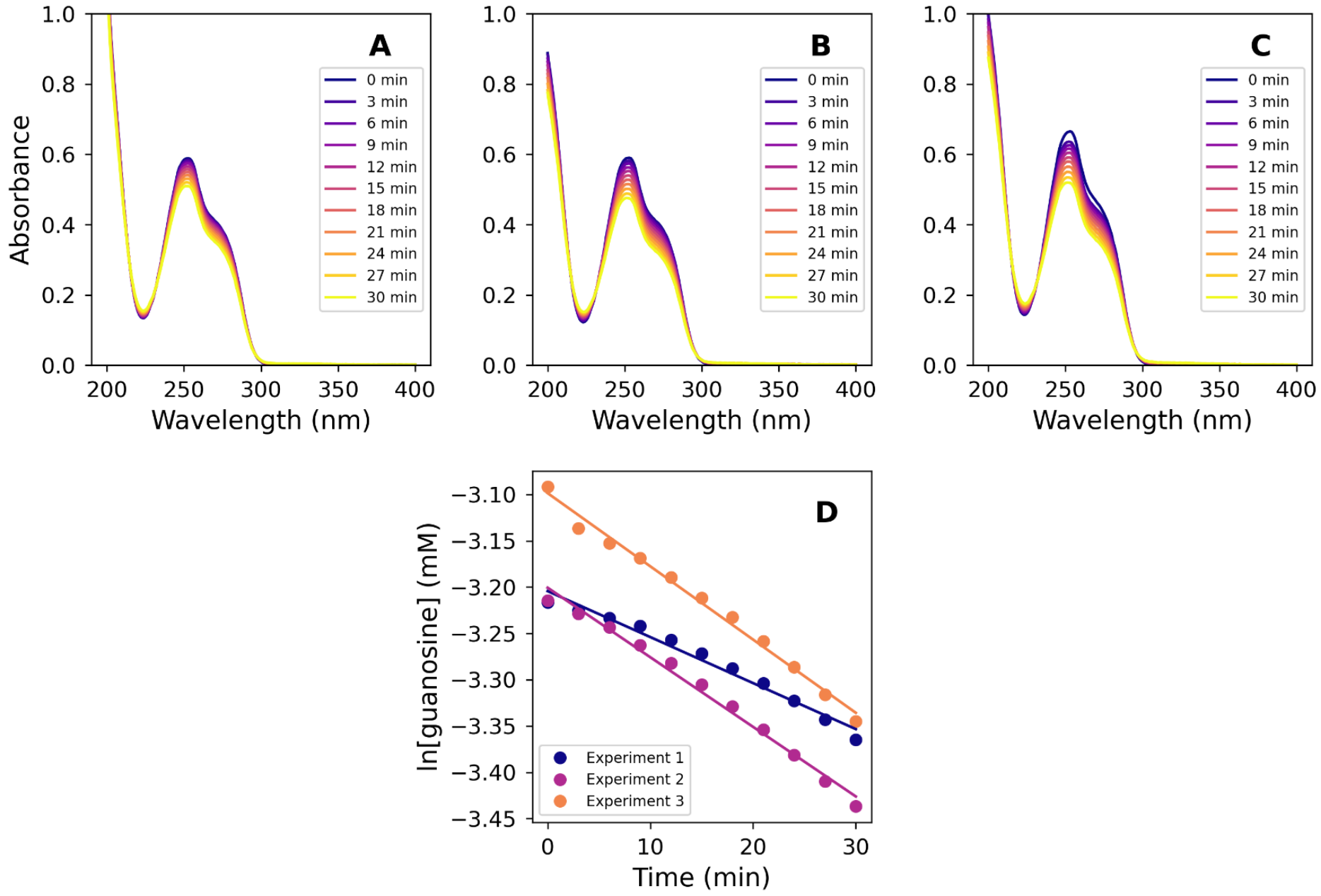

Figure S37: UV-Vis spectra of guanosine throughout irradiation for individual experiments (A-C) and kinetics plot of ln(concentration) vs. time to determine first-order rate constants (D).

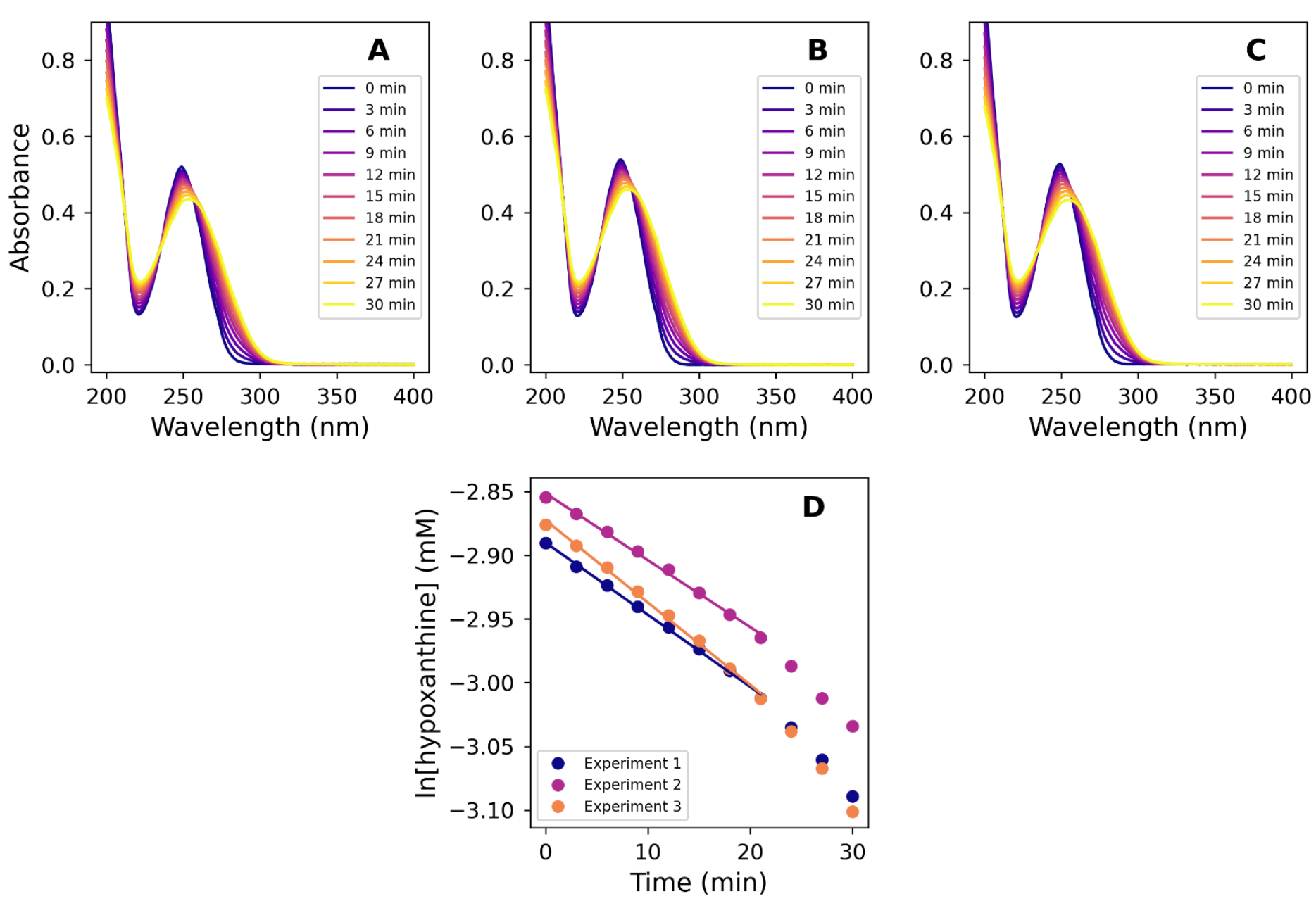

Figure S38: UV-Vis spectra of hypoxanthine throughout irradiation for individual experiments (A-C) and kinetics plot of ln(concentration) vs. time to determine first-order rate constants (D).

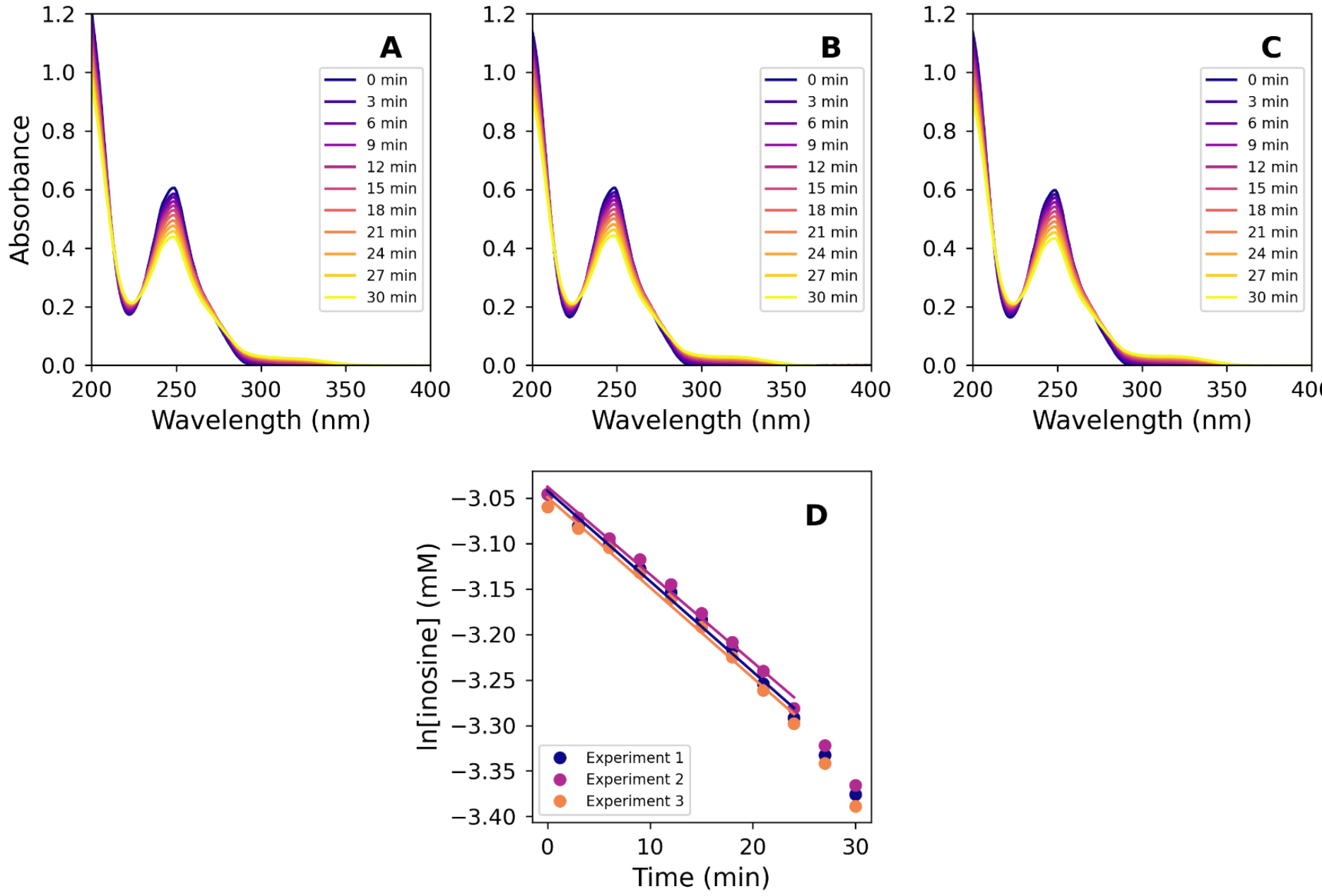

Figure S39: UV-Vis spectra of inosine throughout irradiation for individual experiments (A-C) and kinetics plot of ln(concentration) vs. time to determine first-order rate constants (D).

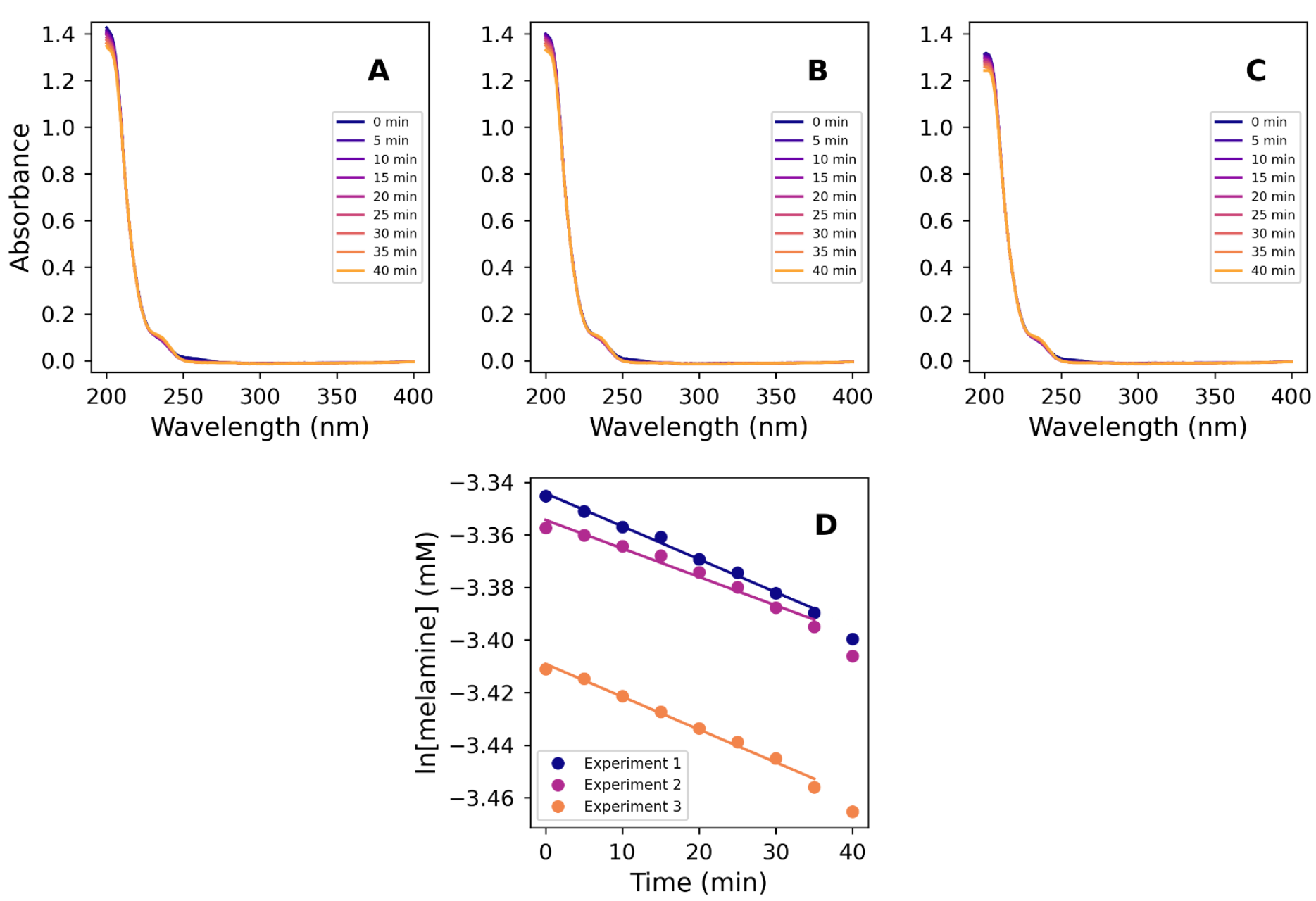

Figure S40: UV-Vis spectra of melamine throughout irradiation for individual experiments (A-C) and kinetics plot of ln(concentration) vs. time to determine first-order rate constants (D).

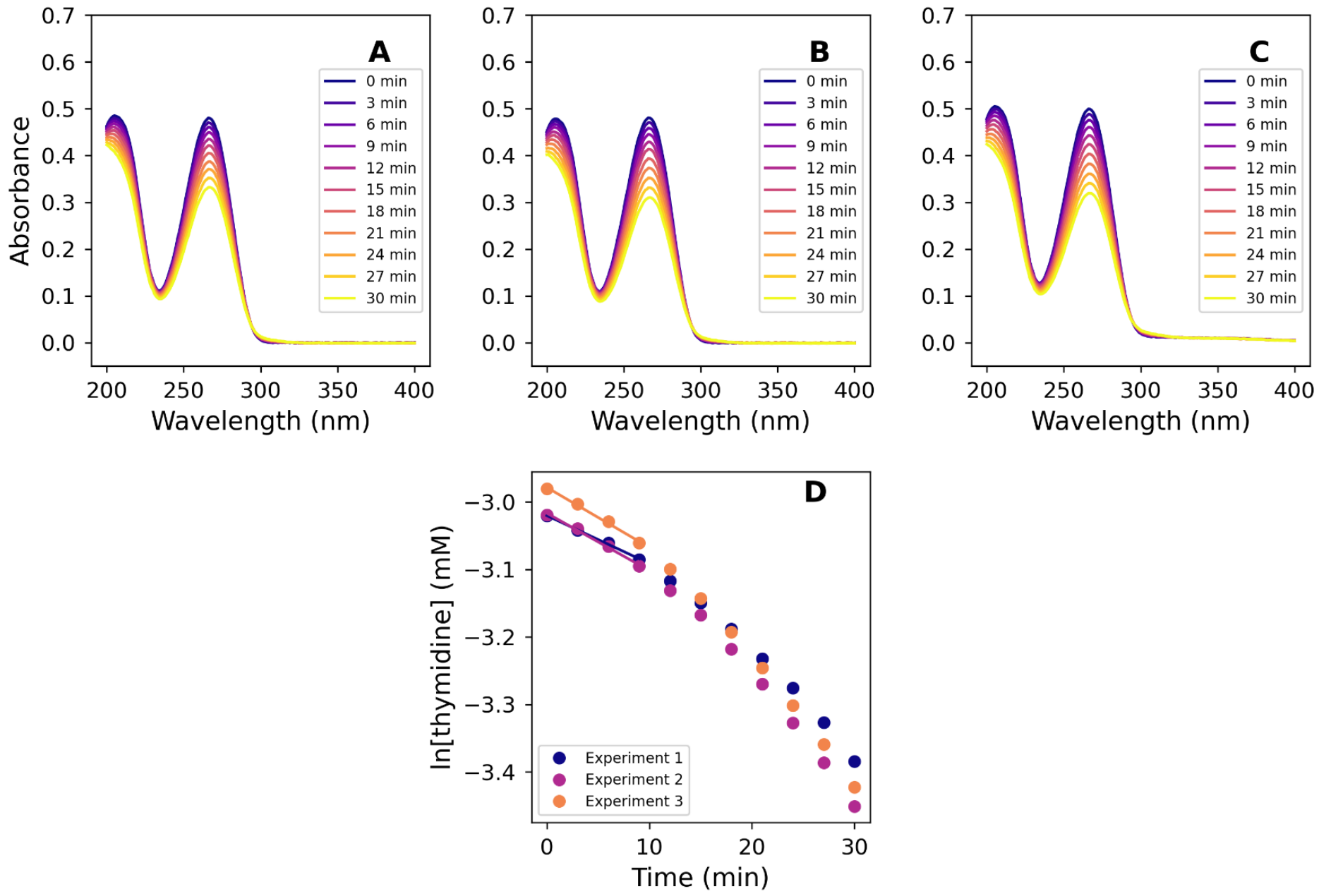

Figure S41: UV-Vis spectra of thymidine throughout irradiation for individual experiments (A-C) and kinetics plot of ln(concentration) vs. time to determine first-order rate constants (D).

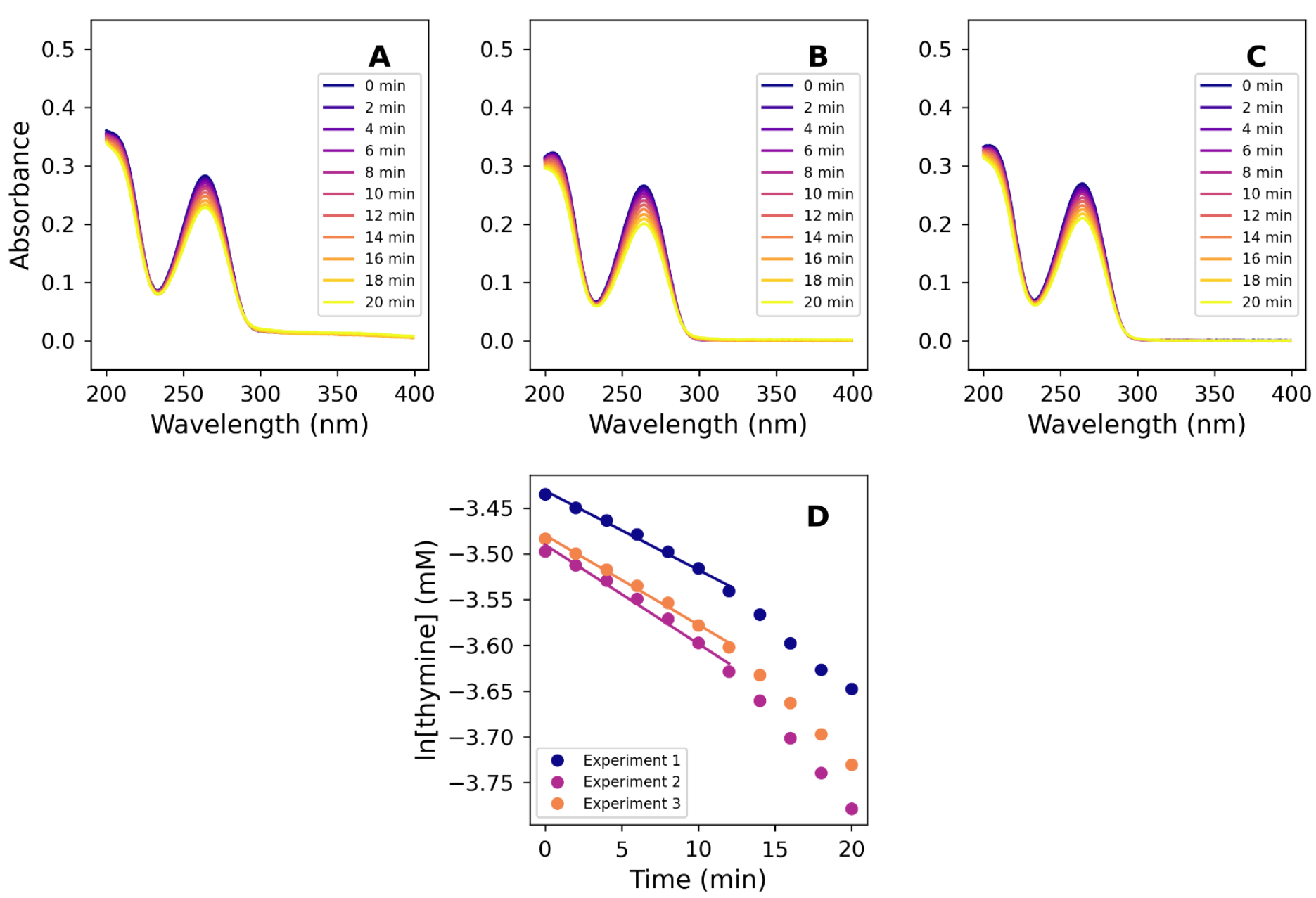

Figure S42: UV-Vis spectra of thymine throughout irradiation for individual experiments (A-C) and kinetics plot of ln(concentration) vs. time to determine first-order rate constants (D).

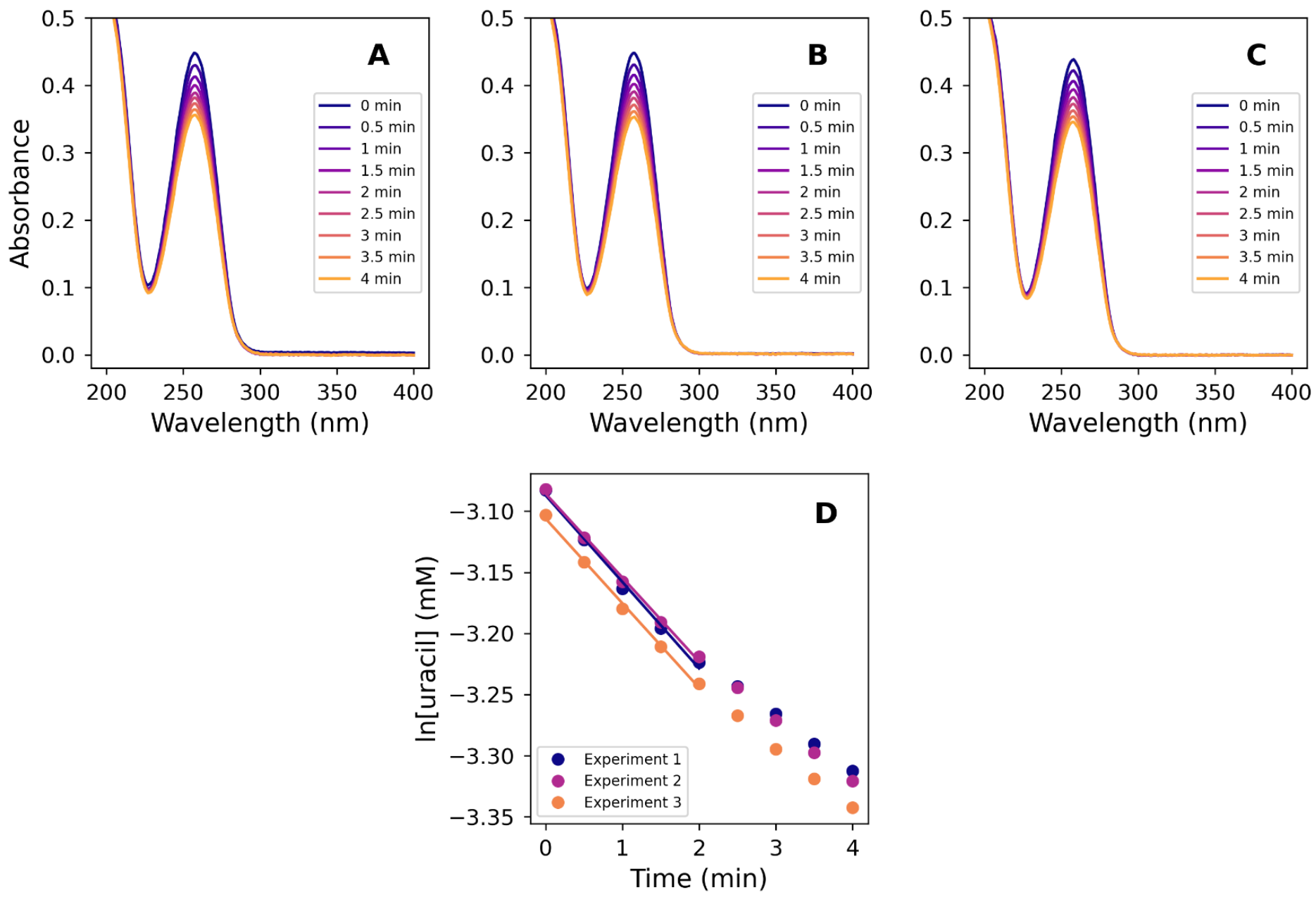

Figure S43: UV-Vis spectra of uracil throughout irradiation for individual experiments (A-C) and kinetics plot of ln(concentration) vs. time to determine first-order rate constants (D).

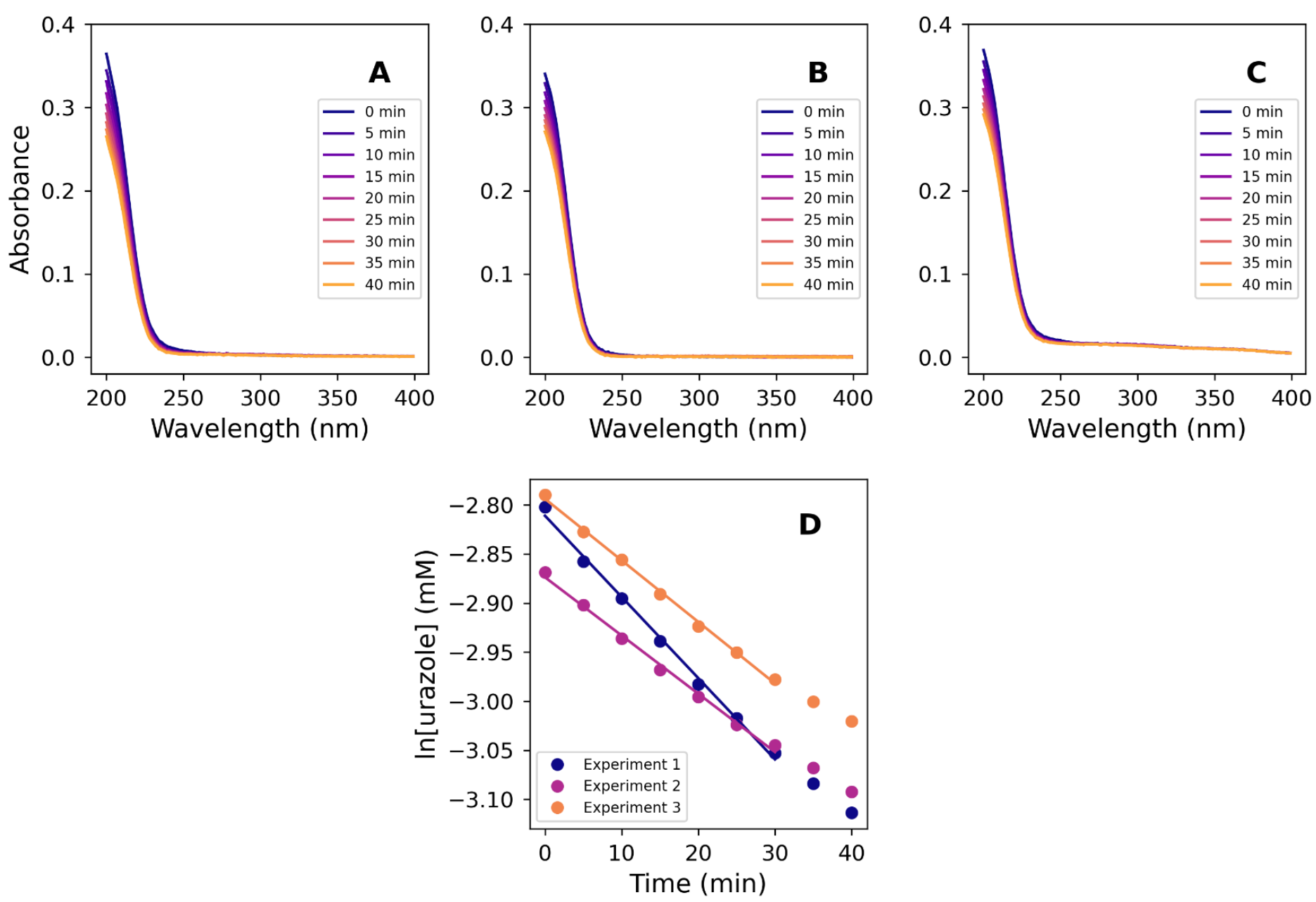

Figure S44: UV-Vis spectra of urazole throughout irradiation for individual experiments (A-C) and kinetics plot of ln(concentration) vs. time to determine first-order rate constants (D).

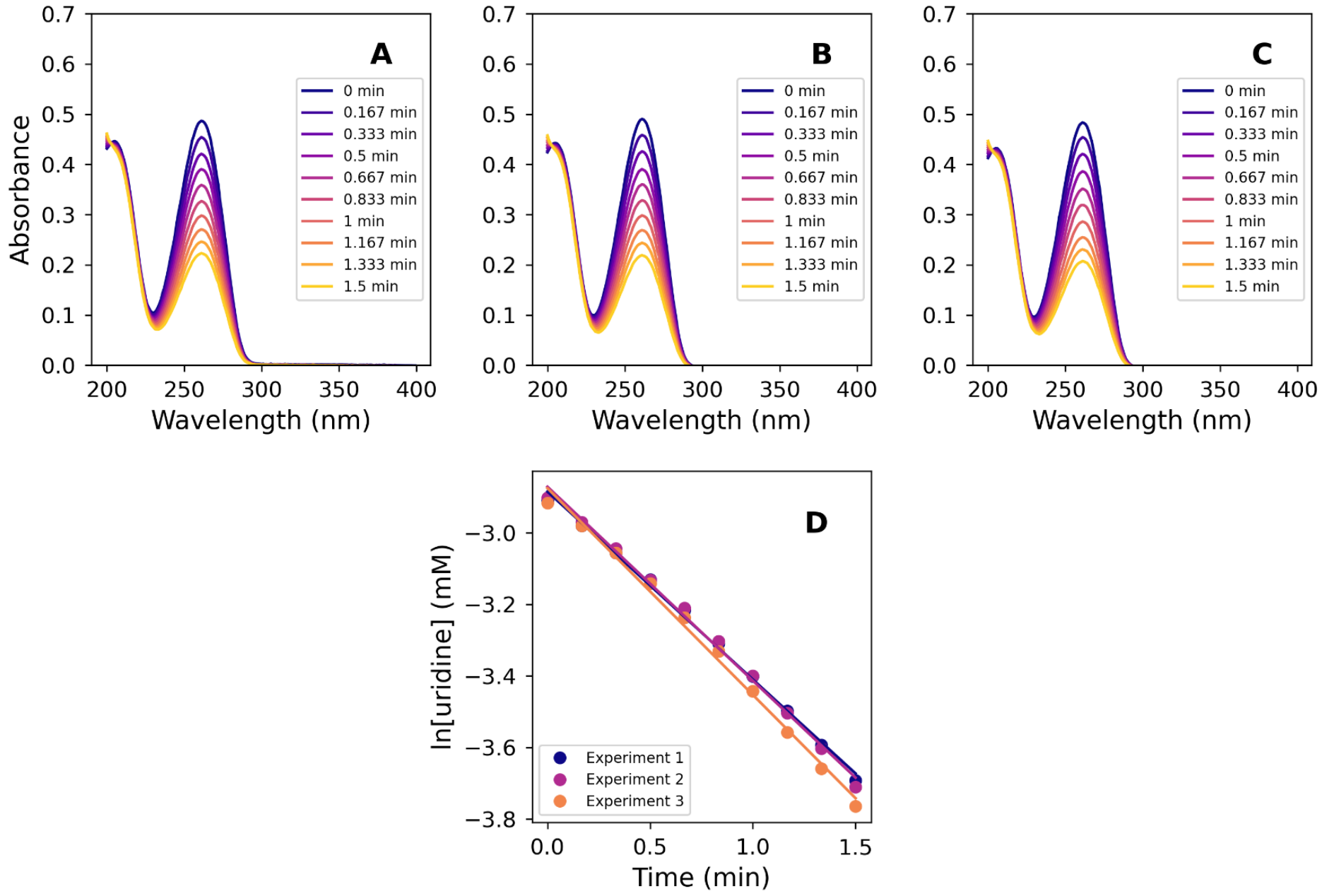

Figure S45: UV-Vis spectra of uridine throughout irradiation for individual experiments (A-C) and kinetics plot of ln(concentration) vs. time to determine first-order rate constants (D).

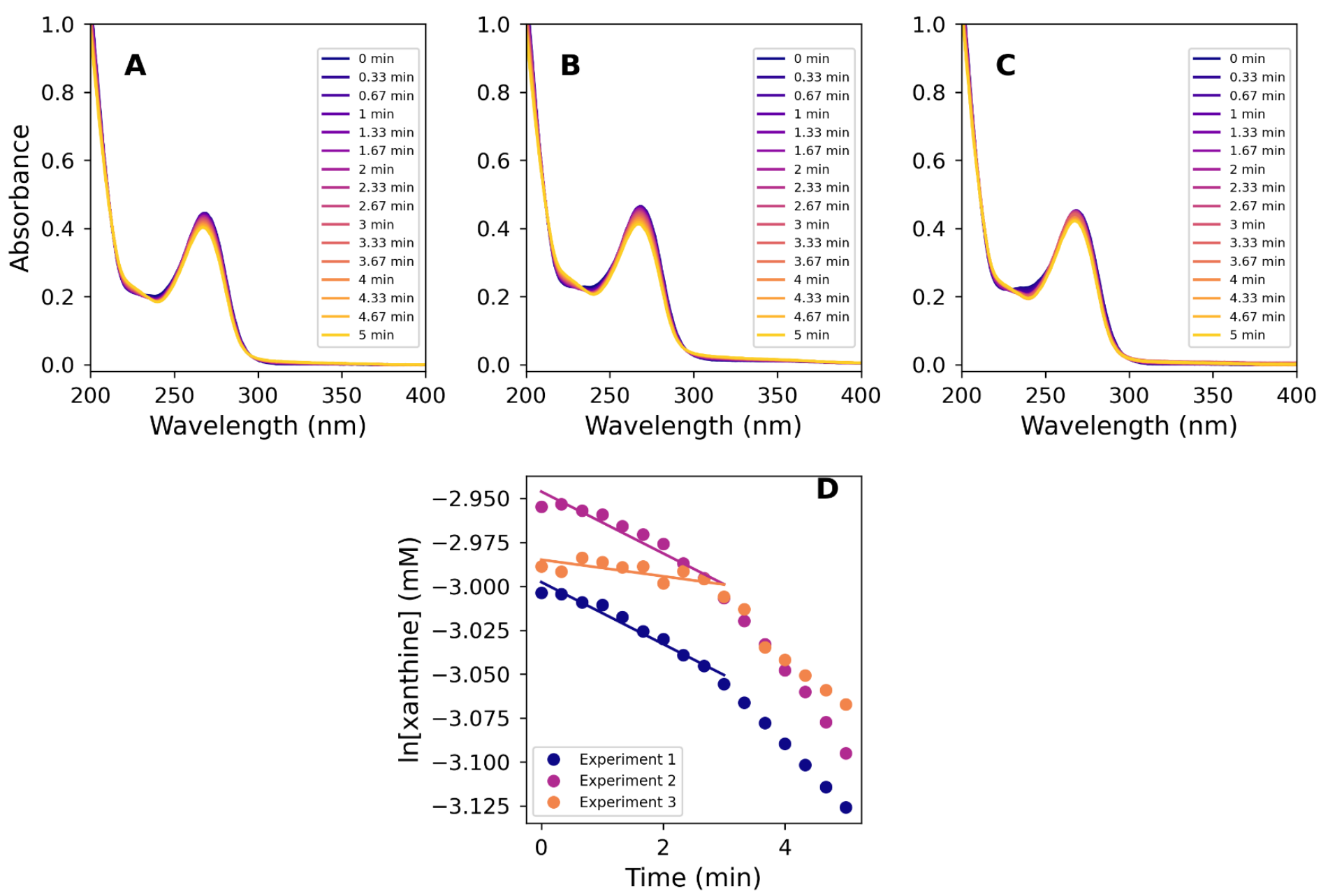

Figure S46: UV-Vis spectra of xanthine throughout irradiation for individual experiments (A-C) and kinetics plot of ln(concentration) vs. time to determine first-order rate constants (D).

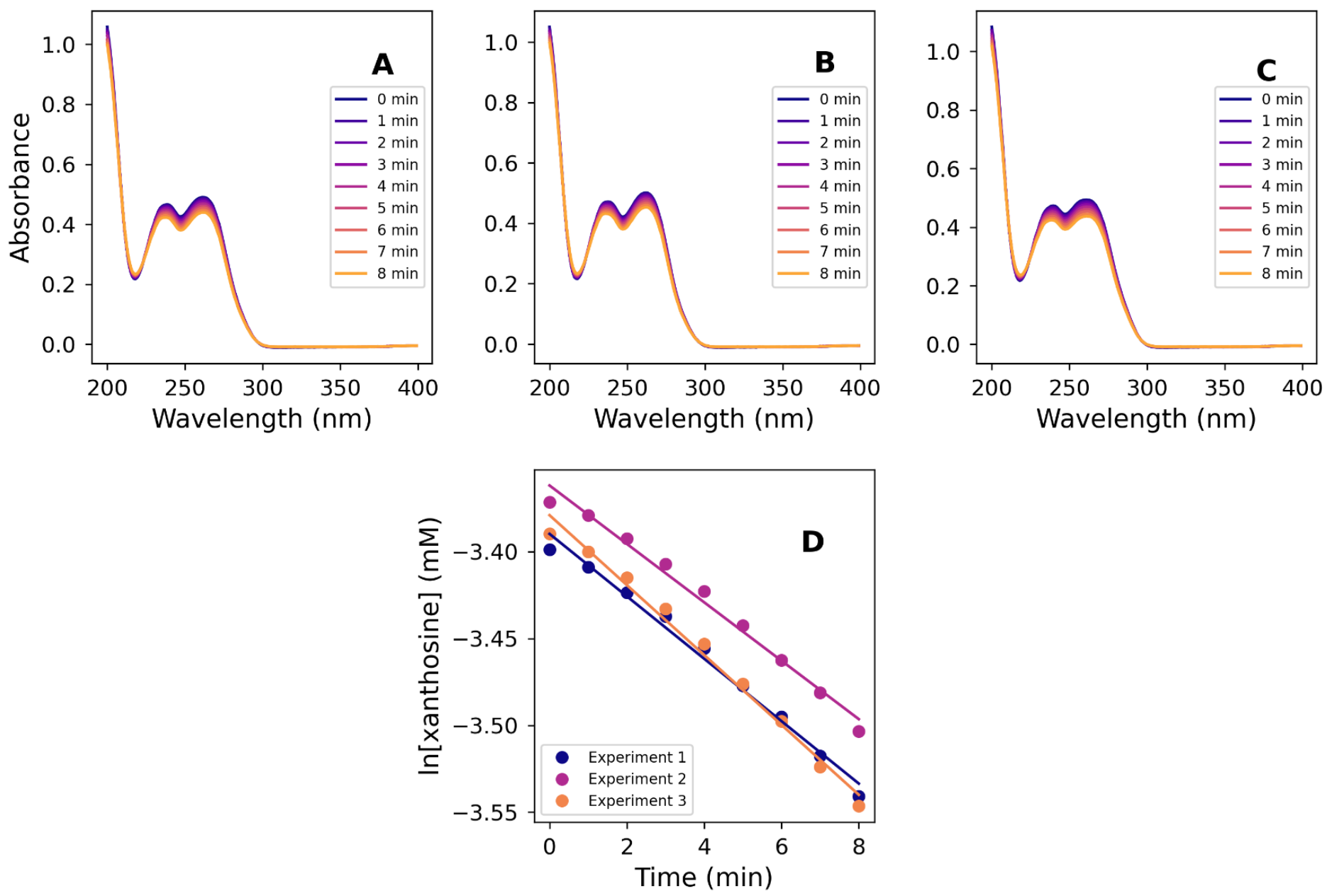

Figure S47: UV-Vis spectra of xanthosine throughout irradiation for individual experiments (A-C) and kinetics plot of ln(concentration) vs. time to determine first-order rate constants (D).

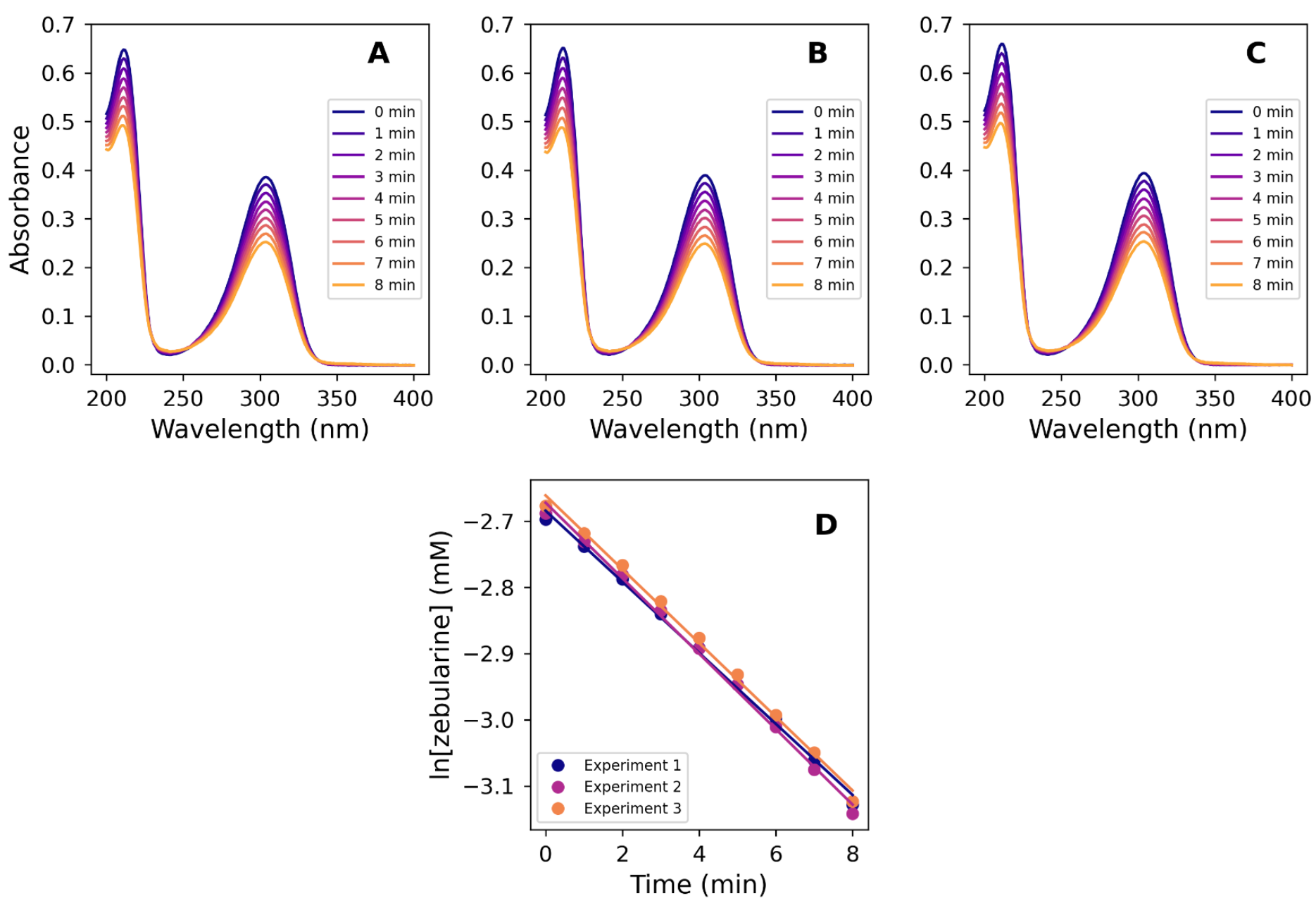

Figure S48: UV-Vis spectra of zebularine throughout irradiation for individual experiments (A-C) and kinetics plot of ln(concentration) vs. time to determine first-order rate constants (D).

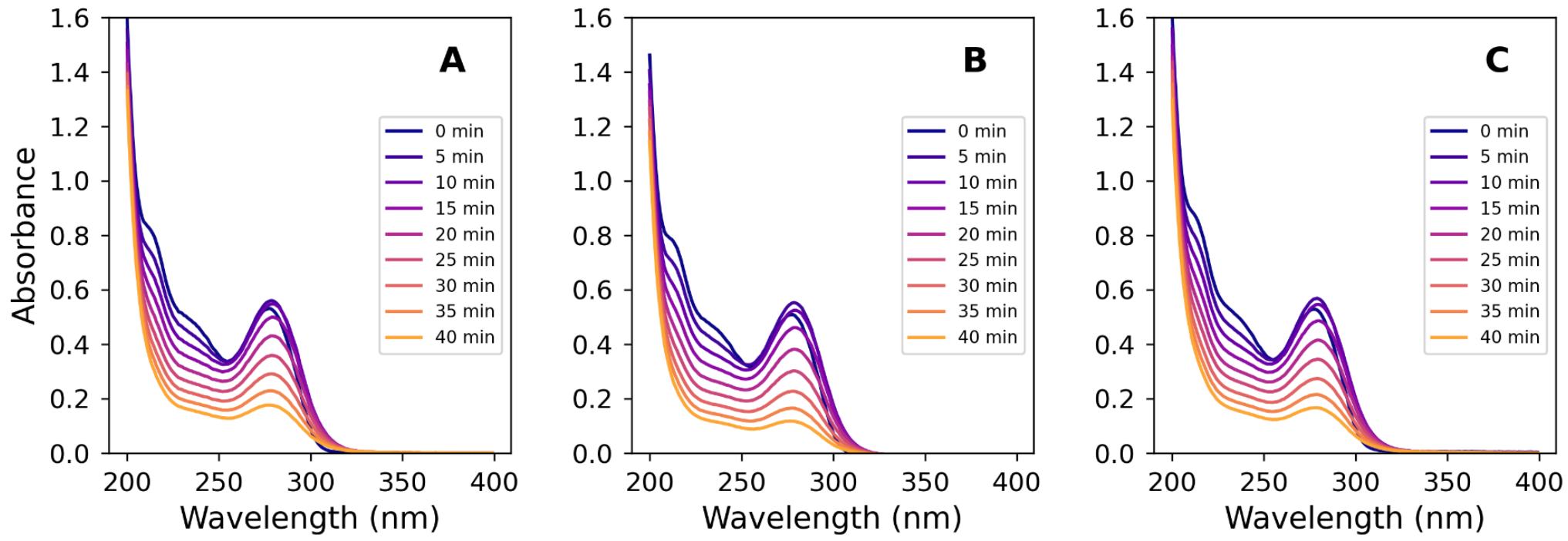

Figure S49: UV-Vis spectra of 5-methylcytidine throughout irradiation for individual experiments (A-C). Note that we could not calculate rates of photodegradation due to shifting absorption features upon irradiation of 5-methylcytosine.

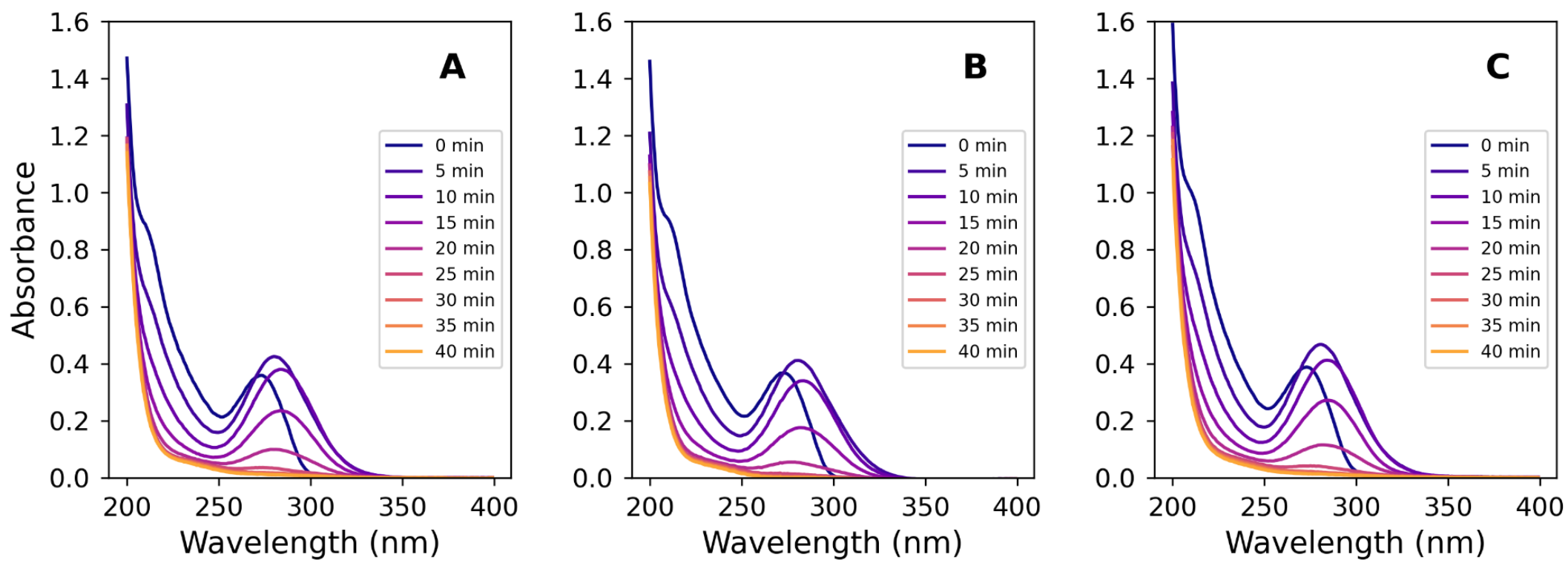

Figure S50: UV-Vis spectra of 5-methylcytosine throughout irradiation for individual experiments (A-C). Note that we could not calculate rates of photodegradation due to shifting absorption features upon irradiation of 5-methylcytidine.

IV. Rate constants and concentrations from individual experiments

Here, we tabulate the rate constants (Table S3) and concentrations (Table S4) from each experiment in the triplicate set for all molecules.

Table S3: Tabulated rate constants for each experiment in the triplicate sets for all molecules.

**Molecule**	**Rate constants *k* (min^{-1})**		
2,4,6-triaminopyrimidine	**Exp 1:** 4.09×10^{-2}	**Exp 2:** 5.92×10^{-2}	**Exp 3:** 7.18×10^{-2}
2,6-diaminopurine	**Exp 1:** 1.60×10^{-2}	**Exp 2:** 1.59×10^{-2}	**Exp 3:** 1.57×10^{-2}
2-aminoadenosine	**Exp 1:** 9.58×10^{-3}	**Exp 2:** 8.66×10^{-3}	**Exp 3:** 9.43×10^{-3}
2-hydroxypyrimidine	**Exp 1:** 3.54×10^{-1}	**Exp 2:** 3.54×10^{-1}	**Exp 3:** 4.49×10^{-1}
2-thiocytosine	**Exp 1:** 9.25×10^{-2}	**Exp 2:** 9.03×10^{-2}	**Exp 3:** 8.58×10^{-2}
2-thiouracil	**Exp 1:** 4.64×10^{-2}	**Exp 2:** 4.39×10^{-2}	**Exp 3:** 4.20×10^{-2}
Adenine	**Exp 1:** 2.00×10^{-3}	**Exp 2:** 2.39×10^{-3}	**Exp 3:** 2.19×10^{-3}
Adenosine	**Exp 1:** 6.23×10^{-4}	**Exp 2:** 8.34×10^{-4}	**Exp 3:** 1.06×10^{-3}
Barbituric acid	**Exp 1:** 4.32×10^{-2}	**Exp 2:** 6.61×10^{-2}	**Exp 3:** 7.14×10^{-2}
Cyanuric acid	**Exp 1:** 6.46×10^{-3}	**Exp 2:** 8.10×10^{-3}	**Exp 3:** 9.64×10^{-3}
Cytidine	**Exp 1:** 2.32×10^{-1}	**Exp 2:** 2.18×10^{-2}	**Exp 3:** 2.06×10^{-2}
Cytosine	**Exp 1:** 3.67×10^{-2}	**Exp 2:** 4.02×10^{-2}	**Exp 3:** 3.60×10^{-2}
Guanosine	**Exp 1:** 4.97×10^{-3}	**Exp 2:** 7.52×10^{-3}	**Exp 3:** 7.91×10^{-3}
Hypoxanthine	**Exp 1:** 5.66×10^{-3}	**Exp 2:** 5.25×10^{-3}	**Exp 3:** 6.46×10^{-3}
Inosine	**Exp 1:** 9.96×10^{-3}	**Exp 2:** 9.64×10^{-3}	**Exp 3:** 9.93×10^{-3}
Melamine	**Exp 1:** 1.25×10^{-3}	**Exp 2:** 1.08×10^{-3}	**Exp 3:** 1.25×10^{-3}
Thymidine	**Exp 1:** 7.11×10^{-3}	**Exp 2:** 8.49×10^{-3}	**Exp 3:** 8.89×10^{-3}
Thymine	**Exp 1:** 8.64×10^{-3}	**Exp 2:** 1.08×10^{-2}	**Exp 3:** 9.80×10^{-3}
Uracil	**Exp 1:** 7.08×10^{-2}	**Exp 2:** 6.86×10^{-2}	**Exp 3:** 6.91×10^{-2}

Urazole	**Exp 1:** 8.28×10^{-3}	**Exp 2:** 5.94×10^{-3}	**Exp 3:** 6.27×10^{-3}
Uridine	**Exp 1:** 5.25×10^{-1}	**Exp 2:** 5.42×10^{-1}	**Exp 3:** 5.78×10^{-1}
Xanthine	**Exp 1:** 1.76×10^{-2}	**Exp 2:** 1.76×10^{-2}	**Exp 3:** 4.71×10^{-3}
Xanthosine	**Exp 1:** 1.80×10^{-2}	**Exp 2:** 1.68×10^{-2}	**Exp 3:** 2.01×10^{-2}
Zebularine	**Exp 1:** 5.37×10^{-2}	**Exp 2:** 5.70×10^{-2}	**Exp 3:** 5.57×10^{-2}

Table S4: Initial concentrations for individual experiments in the triplicate sets for all molecules.

**Molecule**	**Initial Concentrations (μM)**		
2,4,6-triaminopyrimidine	**Exp 1:** 49.1	**Exp 2:** 48.0	**Exp 3:** 48.7
2,6-diaminopurine	**Exp 1:** 55.8	**Exp 2:** 55.4	**Exp 3:** 55.7
2-aminoadenosine	**Exp 1:** 48.0	**Exp 2:** 50.3	**Exp 3:** 48.4
2-hydroxypyrimidine	**Exp 1:** 49.4	**Exp 2:** 52.0	**Exp 3:** 49.7
2-thiocytosine	**Exp 1:** 37.8	**Exp 2:** 36.8	**Exp 3:** 36.6
2-thiouracil	**Exp 1:** 46.5	**Exp 2:** 47.5	**Exp 3:** 46.9
Adenine	**Exp 1:** 51.4	**Exp 2:** 51.4	**Exp 3:** 52.3
Adenosine	**Exp 1:** 45.0	**Exp 2:** 45.5	**Exp 3:** 46.3
Barbituric acid	**Exp 1:** 45.0	**Exp 2:** 45.2	**Exp 3:** 45.3
Cyanuric acid	**Exp 1:** 66.6	**Exp 2:** 57.7	**Exp 3:** 53.1
Cytidine	**Exp 1:** 39.4	**Exp 2:** 40.3	**Exp 3:** 40.1

Cytosine	**Exp 1:** 32.0	**Exp 2:** 32.6	**Exp 3:** 33.7
Guanosine	**Exp 1:** 40.1	**Exp 2:** 40.2	**Exp 3:** 45.4
Hypoxanthine	**Exp 1:** 55.6	**Exp 2:** 57.6	**Exp 3:** 56.4
Inosine	**Exp 1:** 47.6	**Exp 2:** 47.6	**Exp 3:** 46.9
Melamine	**Exp 1:** 35.3	**Exp 2:** 34.8	**Exp 3:** 33.0
Thymidine	**Exp 1:** 48.8	**Exp 2:** 48.9	**Exp 3:** 50.7
Thymine	**Exp 1:** 32.2	**Exp 2:** 30.3	**Exp 3:** 30.7
Uracil	**Exp 1:** 45.8	**Exp 2:** 45.9	**Exp 3:** 44.9
Urazole	**Exp 1:** 60.7	**Exp 2:** 56.8	**Exp 3:** 61.4
Uridine	**Exp 1:** 54.5	**Exp 2:** 54.9	**Exp 3:** 54.1
Xanthine	**Exp 1:** 49.6	**Exp 2:** 52.1	**Exp 3:** 50.4
Xanthosine	**Exp 1:** 33.4	**Exp 2:** 34.3	**Exp 3:** 33.7
Zebularine	**Exp 1:** 67.4	**Exp 2:** 68.0	**Exp 3:** 68.8

V. pH of samples before and after irradiation

Here, we tabulate the average initial and final pH values of all samples. The pH of each replicate for a given molecule was measured initially before irradiation, and the average of the three samples is reported here. Only 1 mL of sample was irradiated for each replicate, and our pH meter requires >2 mL for an accurate measurement. So, to measure the final pH, all three replicates were pooled together and the pH of the combined sample was measured. Multiple readings (n=6) were taken of this sample, which were then averaged together to yield the pH values shown here. Some molecules showed a small drift in pH in either direction upon irradiation. Only three molecules had a final pH >1 pH unit from the initial pH: cytidine, inosine, and uridine. It should be noted

that pH measurements of unbuffered solutions are challenging using a pH meter, so these pH readings should be interpreted as approximate.

To ensure that the changing pH was not playing a role in the measured degradation during irradiation experiments, we constructed solutions of 50 μM of cytidine or uridine (initial pH 6.15 and 6.29, respectively). We then adjusted the pH to near the final value observed after irradiation using small volumes of NaOH (pH of 7.30 and 6.95 for cytidine and uridine, respectively). We then monitored the UV-Vis spectra of these samples over the same time period as the irradiations, but when the samples were left in the dark. We saw no significant degradation or changes in the UV-Vis spectra as a function of time at the adjusted pH values, indicating that pH changes do not play a significant role in the degradation we observe during the irradiations.

Table S5: Average Initial and Final pH values

Molecule	Avg. Initial pH	Avg. Final pH
2,4,6-triaminopyrimidine	6.69	6.65
2,6-diaminopurine	6.77	6.28
2-aminoadenosine	7.34	7.23
2-hydroxypyrimidine	4.62	4.75
2-thiocytosine	6.71	6.17
2-thiouracil	5.90	4.83
5-methylcytidine	N/A	N/A
5-methylcytosine	N/A	N/A
Adenine	4.69	4.80
Adenosine	5.53	5.44
Barbituric acid	4.49	4.78
Cyanuric acid	7.34 (adjusted)	7.18
Cytidine	6.25	7.35
Cytosine	6.61	6.85
Guanosine	7.14	6.73
Hypoxanthine	6.46	6.93
Inosine	6.29	6.94
Melamine	5.93	5.98
Thymidine	6.03	5.78
Thymine	6.24	6.37
Uracil	5.75	6.19
Urazole	5.47	5.33
Uridine	5.49	6.85
Xanthine	7.55	7.15
Xanthosine	5.46	5.42
Zebularine	5.78	6.11

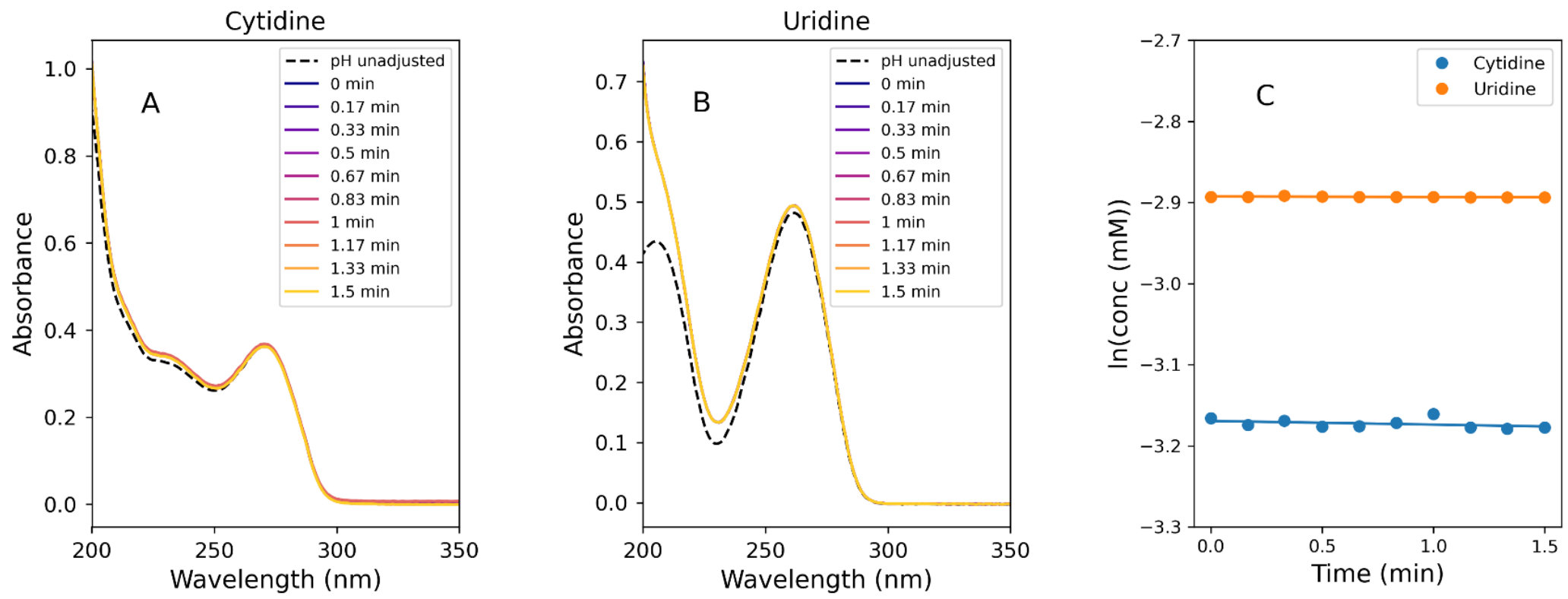

Figure S51: (A) Cytidine pH control test. The initial pH (6.15, black dashed line) was adjusted to approximately 7.30 by adding small volumes of 0.1 M NaOH. This solution was then allowed to sit in the dark for 1.5 minutes total, taking UV-Vis spectra every 10 seconds to match the frequency and duration of the irradiation experiments. (B) Uridine pH control test. The solution (initial pH 6.29, black dashed line) was adjusted to pH 6.95 by adding small volumes of 0.1 M NaOH. This solution was then allowed to sit in the dark for 1.5 minutes total, taking UV-Vis spectra every 10 seconds to match the frequency and duration of the irradiation experiments. (C) Plot of ln(concentration) vs. time, where concentrations were calculated using the standard curves for each molecule. Linear trendlines were fit to calculated the "rate constant" for each molecule at the adjusted pH. The variance in the slopes were also calculated from the goodness of the fit to estimate the uncertainties. For uridine, we find $k_{pH,uridine} = (6.15 \pm 0.0011) \times 10^{-4}\ min^{-1}$ and for cytidine, we find $k_{pH,cytidine} = (4.51 \pm 0.015) \times 10^{-3}\ min^{-1}$. These values for pH-dependent changes are two and three orders of magnitude smaller than the rate constants for photodegradation ($k_{cytidine} = 0.218 \pm 0.00755\ min^{-1}$ and $k_{uridine} = 0.548 \pm 0.0154\ min^{-1}$), respectively. This indicates that the change in pH upon irradiation does not play a significant role in the degradation of the molecules.

VI. Irradiation Geometry and Actinometry to Determine Photon Flux

The geometry of the irradiation setup can influence the photon flux incident upon the samples, and therefore affect the measured degradation rate constants. It is thus important to ensure uniform irradiation of the samples and have an accurate estimate for the actinic photon flux, which we describe here.

The irradiation experiments were carried out in a Rayonet RPR-200 reactor, with samples placed in a RMA-500 sample holder (Figure S52), which was allowed to spin during irradiation. The spinning of the sample holder ensures consistent irradiation of all samples, such that variations in lamp brightness or positioning relative to the lamps does not cause artifacts in the measured rate constants.

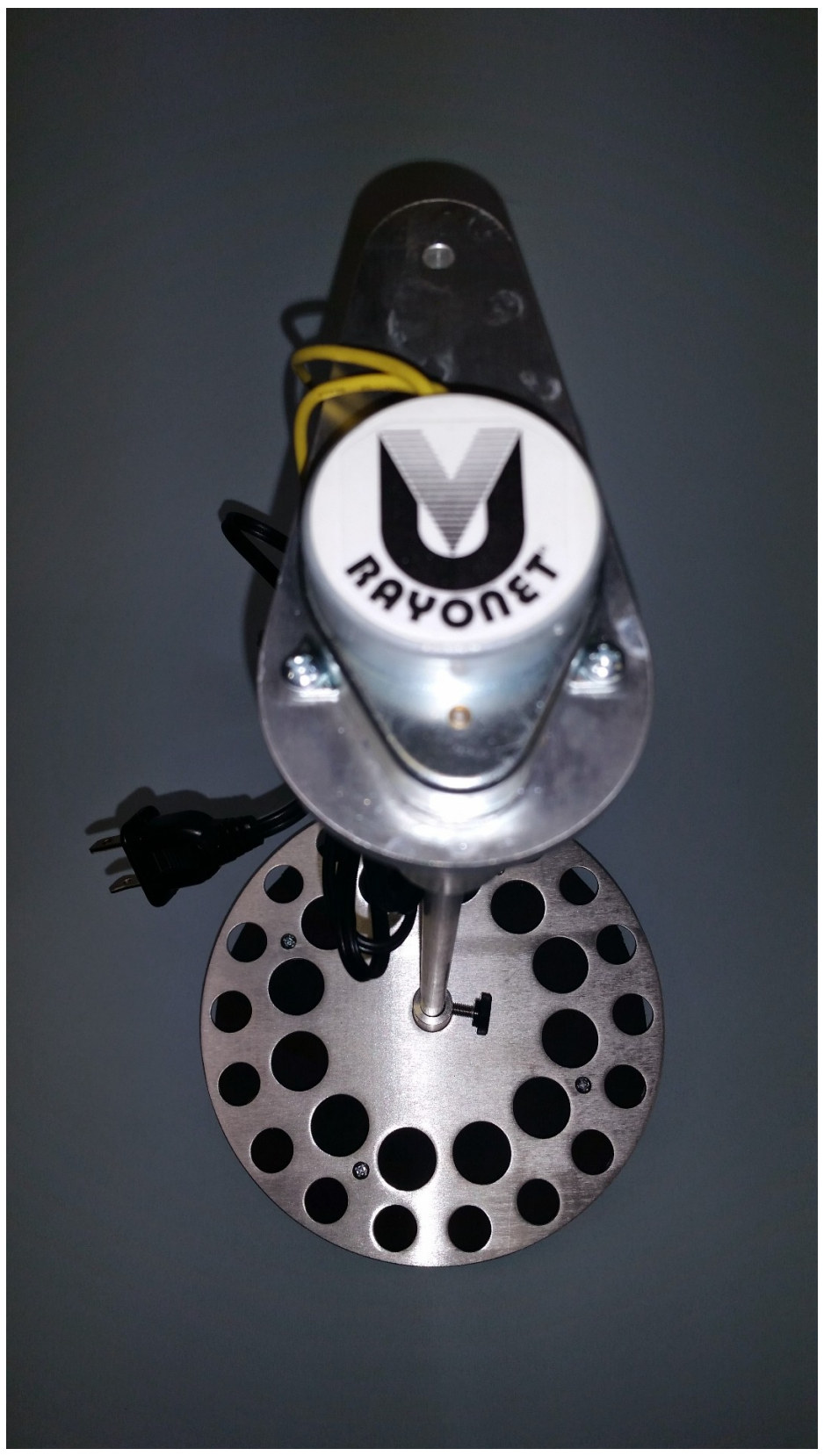

Figure S52: RMA-500 accessory for the Rayonet RPR-200, which can hold multiple samples and allow for spinning to ensure uniform irradiation. Credit: The Southern New England Ultraviolet Co. (https://www.rayonet.org/product.php?part=RMA-500)

Chemical actinometry is a method that can be used to determine the photon flux ($\frac{phot}{s\,cm^2}$) incident on a sample (1). In order to determine the incident photon flux, we follow the treatment for rectangular vessels (i.e. our cuvettes) from Roca et al. (2):

$$\Phi_X = \frac{\frac{d[X]}{dt}|_0 \times l}{I_0(1-10^{-A})},$$

where Φ_X is the quantum yield of species *X*, $\frac{d[X]}{dt}$ is the change in concentration of species *X* with time during the initial photochemical reaction, l is the path length of the vessel (1 cm in our case

for the cuvettes we use), I_0 is the incident photon flux, and A is the absorbance at the irradiation wavelength, which is 254 nm in our case.
We use uridine as a chemical actinometer, which has a reported quantum yield of 0.025 ± 0.001 under a 254 nm irradiation (3).

We constructed a solution of 50 uM uridine from a 10 mM uridine stock solution, degassed the solution by sparging with nitrogen gas, and added 1 mL to six different cuvettes. The six cuvettes were then placed into our Rayonet reactor with the sample holder spinning. We irradiated the samples for 1.5 minutes total, taking 10 second timepoints where we measured the UV-Vis spectra. These spectra were recorded and converted into the concentration of uridine using our standard curve. Plots of concentration vs. time were made for each of the replicates. Only the first 60 seconds were used to determine the initial rate of degradation ($\frac{d[U]}{dt}|_0$), since some signs of non-linear behavior were observed at later timepoints. The initial absorbance at 254 nm was also recorded for each replicate.
We determined the photon flux for each replicate, according to the following equation:

$$I_0 = \frac{\frac{d[U]}{dt}|_0 \times l}{\Phi_U(1 - 10^{-A_{254}})},$$

We calculated the average photon flux and standard error of the mean to be $(1.67 \pm 0.033) \times 10^{16} \frac{phot}{s\ cm^2}$. This is in good agreement with the expected photon flux quoted for Rayonet RPR-200 reactors at 254 nm of $1.65 \times 10^{16} \frac{phot}{s\ cm^3}$ (Southern New England Ultraviolet Co., https://www.rayonet.org/product.php?part=RPR-200) and our irradiation vessel (a 1 cm path length cuvette).

VII. Calculation of estimated photodegradation under suggested UV environment on early Earth

We estimate the photodegradation rate constants and corresponding half lives of each molecule under the expected UV environment on the surface of the early Earth as follows.

$$k_{tot} = k_{254} \frac{\int_{200}^{400} A_\lambda SI_\lambda d\lambda}{I_{0,254} A_{254}}$$

where k_{tot} is the estimated photodegradation rate constant under the UV environment expected on the early Earth, k_{254} is our experimentally determined rate constant using 254 nm irradiation, A_λ is the wavelength-dependent absorption spectra of a given molecule, SI_λ is the wavelength-dependent surface intensity estimated on the early Earth (in $\frac{phot}{s\ cm^2}$), I_0 is the incident photon flux in our irradiation setup (determined to be $(1.67 \pm 0.033) \times 10^{16} \frac{phot}{s\ cm^2}$ using chemical actinometry), and A_{254} is the absorbance of a given molecule at 254 nm. The spectral surface intensity is calculated for a N_2/CO_2 dominated atmosphere (4-6). To calculate the absorbance-

weighted surface intensity, the average absorbance spectra of each triplicate set is calculated as a function of wavelength. The spectral surface intensity is interpolated along the same wavelengths and the two quantities are multiplied by each other. The integral is calculated as the trapezoidal sum of area under the curve of this product. The absorbance at 254 nm is calculated as the average of the triplicate set for each molecule. We calculate the error on k_{tot} by propagating the errors from k_{254} and A_{254}.

The half-life expected under early Earth UV conditions is calculated as:

$t_{1/2,tot} = \frac{ln(2)}{k_{tot}}$. We compare this to the half-life from our experimental conditions, which is calculated as $t_{1/2,254} = \frac{ln(2)}{k_{254}}$.

VIII. Calculation of Errors

Here, we describe the procedure for calculating errors on experimental rate constants and calculated half lives.

Experimental rate constants and half-lives

Each molecule was tested in triplicate; averages and standard errors were calculated from this triplicate set. We further conservatively estimate a 5% error in weighing out the masses of solids when preparing stock solutions and a 7% error for pipetting. Chemical actinometry was used to determine the incident photon flux of $(1.67 \pm 0.033) \times 10^{16} \frac{phot}{s\ cm^2}$, which yields a 1.97% error. Errors were propagated taking into account all four sources of uncertainty to yield the total estimated error on the rate constants, which we report in the main text.

The errors in the experimental half lives were determined from the same sources of uncertainty as the rate constants. Since half life and rate constant are indirectly proportional, the error in the half life (σ_{thalf}) can simply be calculated as:

$$\frac{\sigma_{thalf}}{t_{half}} = \frac{\sigma_k}{k},$$

Where σ_k is the error in the rate constant, calculated as described previously.

Estimated rate constants and half-lives under UV environment on early Earth

For the calculation of estimated photodegradation under an early Earth UV environment, uncertainties were determined as follows. The errors from our experimentally determined rate constants (σ_k, calculated as described previously and now denoted as $\sigma_{k,254}$ to indicate these are determined for 254 nm irradiation) were propagated along with errors in the incident photon flux at 254 nm (1.97%, as described previously), and the standard error from the triplicate measurements of A_{254}. The uncertainty in estimated half lives under an early Earth UV

environment is calculated from the uncertainty in the photodegradation rate estimated under early Earth UV in the same way as for the rate constants and half lives under 254 nm irradiation shown above.

IX. Penetration depths of 254 nm photons in samples

Due to the differing absorption properties of each molecule, the 254 nm incident photons will penetrate to different average depths in the samples during an experiment. These differences could play a role in the measured degradation rate constants, which introduces potential caveats to our study. However, since photochemical reactions have been observed to display strong, non-linear concentration dependencies (7,8), we intentionally choose to hold concentrations constant at 50 μM to allow for comparison of photostability on a per molecule basis. Nevertheless, the complete picture of photostability is undoubtedly more complicated, which our estimate of the total photodegradation rate constant under realistic planetary conditions helps to address.

Here, we calculate the penetration depths of 254 nm photons for each molecule following the treatment described in Todd et al. (9). From the Beer-Lambert Law, we have:

$$log_{10}(\frac{I_0}{I}) = \epsilon cl,$$

where I_0 is the incident spectral intensity, I is the transmitted spectral intensity, ϵ is the molar decadic extinction coefficient, c is the concentration, and l is the path length. We define the penetration depth as the path length for the transmitted intensity to drop by a factor of 1/e, i.e.

$$\frac{I}{I_0} = \frac{1}{e}$$

Rearranging and solving for the penetration depth, denoted as δ_p, yields:

$$\delta_p = \frac{-log_{10}(\frac{1}{e})}{\epsilon c} = \frac{0.4343}{\epsilon c}$$

To calculate the penetration depths for our samples, we take the average initial concentration (calculated from the absorbance and standard curves) and the average initial absorbance at 254 nm. We use the Beer-Lambert law to calculate the molar decadic extinction coefficient at 254 nm for each molecule as follows:

$$\epsilon_{254} = \frac{A_{254}}{cl}$$

Then, we calculate the penetration depth for 254 nm photons as:

$$\delta_{p,254} = \frac{0.4343}{\epsilon_{254}c}$$

Table S6 shows the penetration depths of 254 nm photons for all molecules. For comparison, the path length of our irradiation vessels is 1 cm. The penetration depths are generally on the same order or longer than the path length of the cuvettes, indicating that most molecules receive fairly uniform irradiation throughout the sample.

Table S6: Penetration depths of 254 nm photons (in cm) for all samples used in experiments.

**Molecule**	**Penetration depth, 254 nm (cm)**
2,4,6-triaminopyrimidine	1.88
2,6-diaminopurine	1.16
2-aminoadenosine	0.920
2-hydroxypyrimidine	11.8
2-thiocytosine	0.780
2-thiouracil	1.12
Adenine	0.718
Adenosine	0.776
Barbituric acid	0.649
Cyanuric acid	17.5
Cytidine	1.69
Cytosine	1.81
Guanosine	0.708
Hypoxanthine	0.896
Inosine	0.875
Melamine	55.0
Thymidine	1.21
Thymine	1.95
Uracil	1.01
Urazole	46.1
Uridine	1.01
Xanthine	1.42
Xanthosine	0.929
Zebularine	12.2

X. Dilution spectra of all molecules

To assess the possible influence of degradation products influencing the absorption spectra obtained throughout the course of the irradiation experiments, we also performed serial dilutions of each molecule and measured their absorption spectra. Comparisons between these spectra can suggest when formation of degradation products may be significantly influencing the UV-Vis absorption spectra.

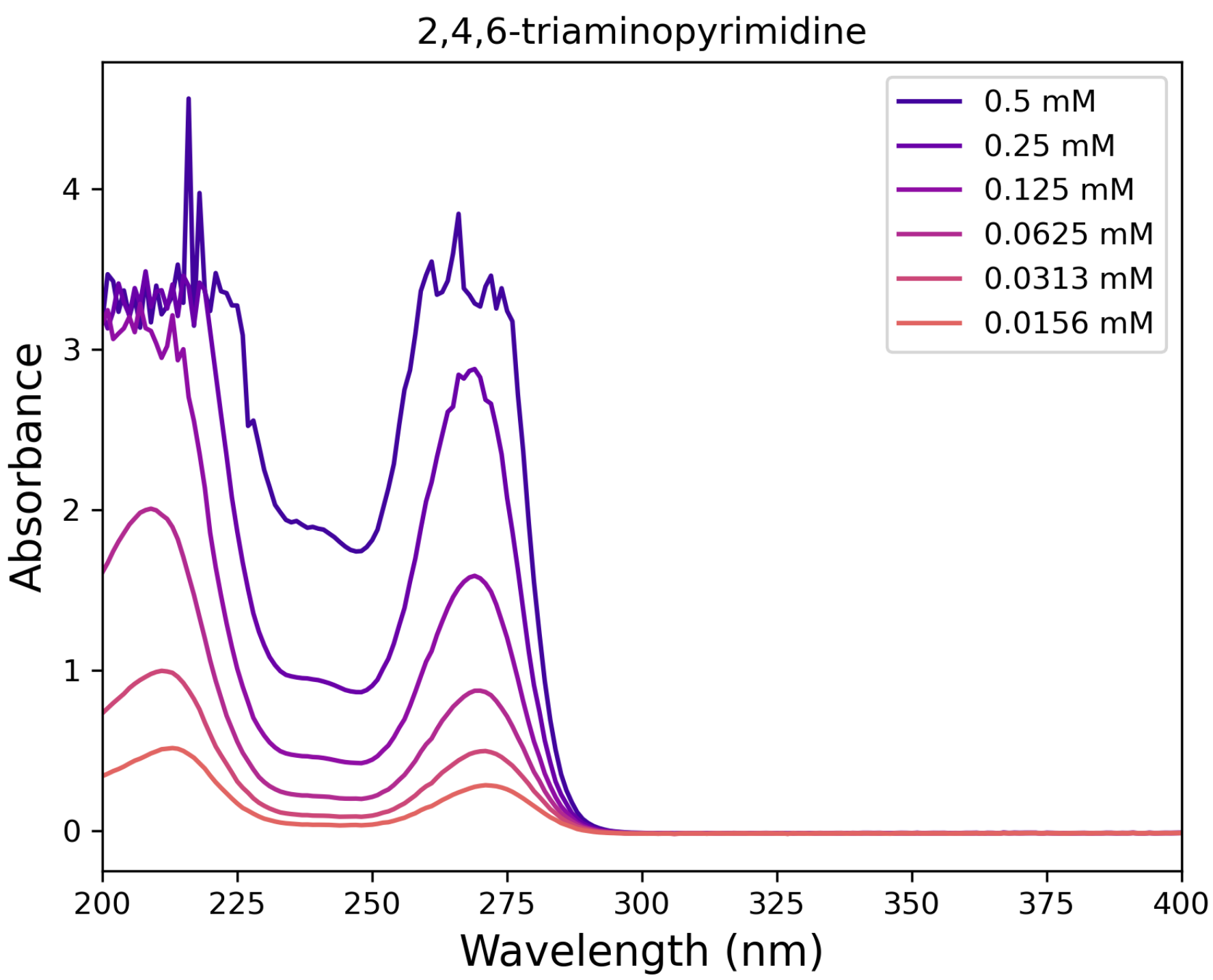

Figure S53: Absorbance spectra of a serial dilution of 2,4,6-triaminopyrimidine.

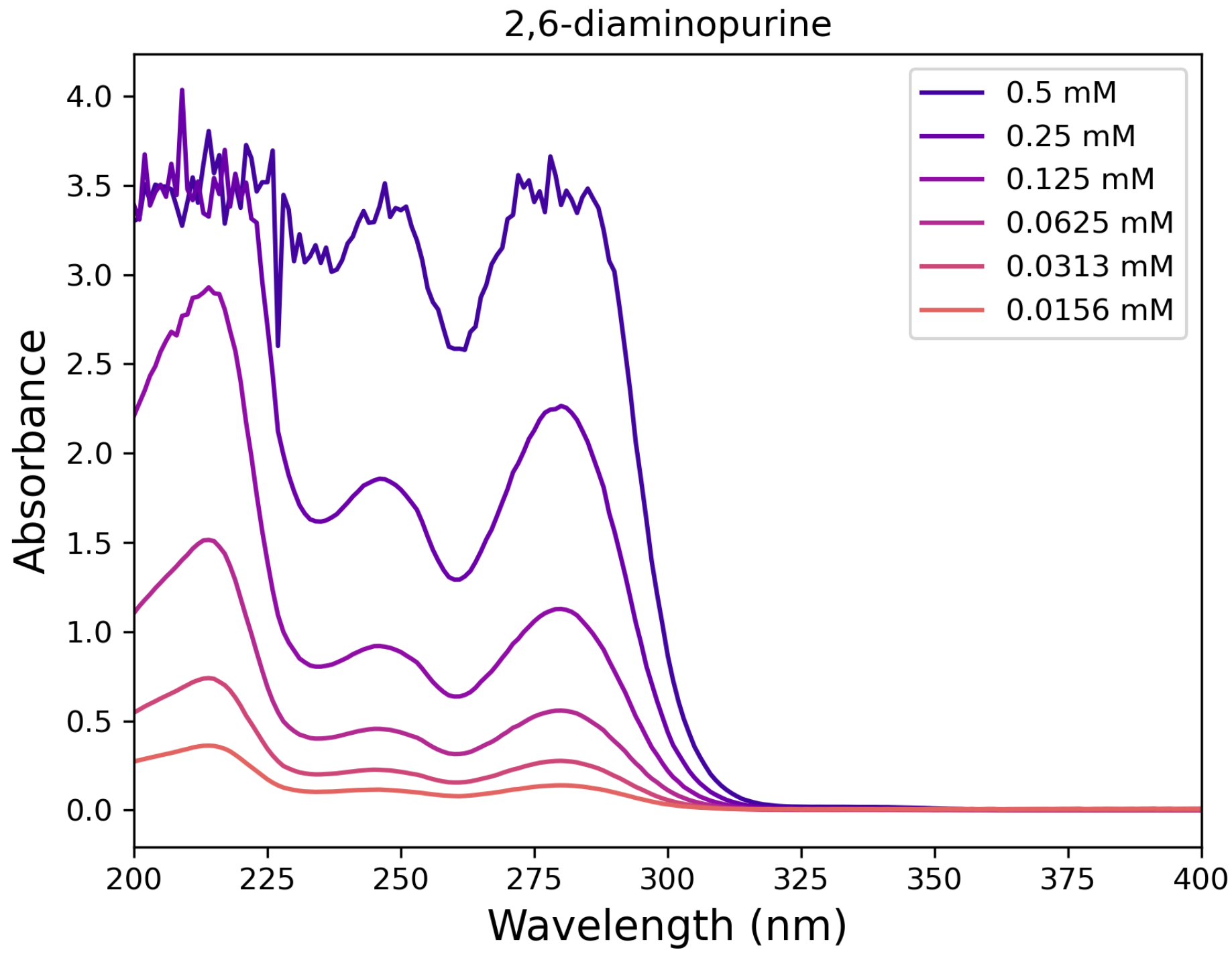

Figure S54: Absorbance spectra of a serial dilution of 2,6-diaminopurine.

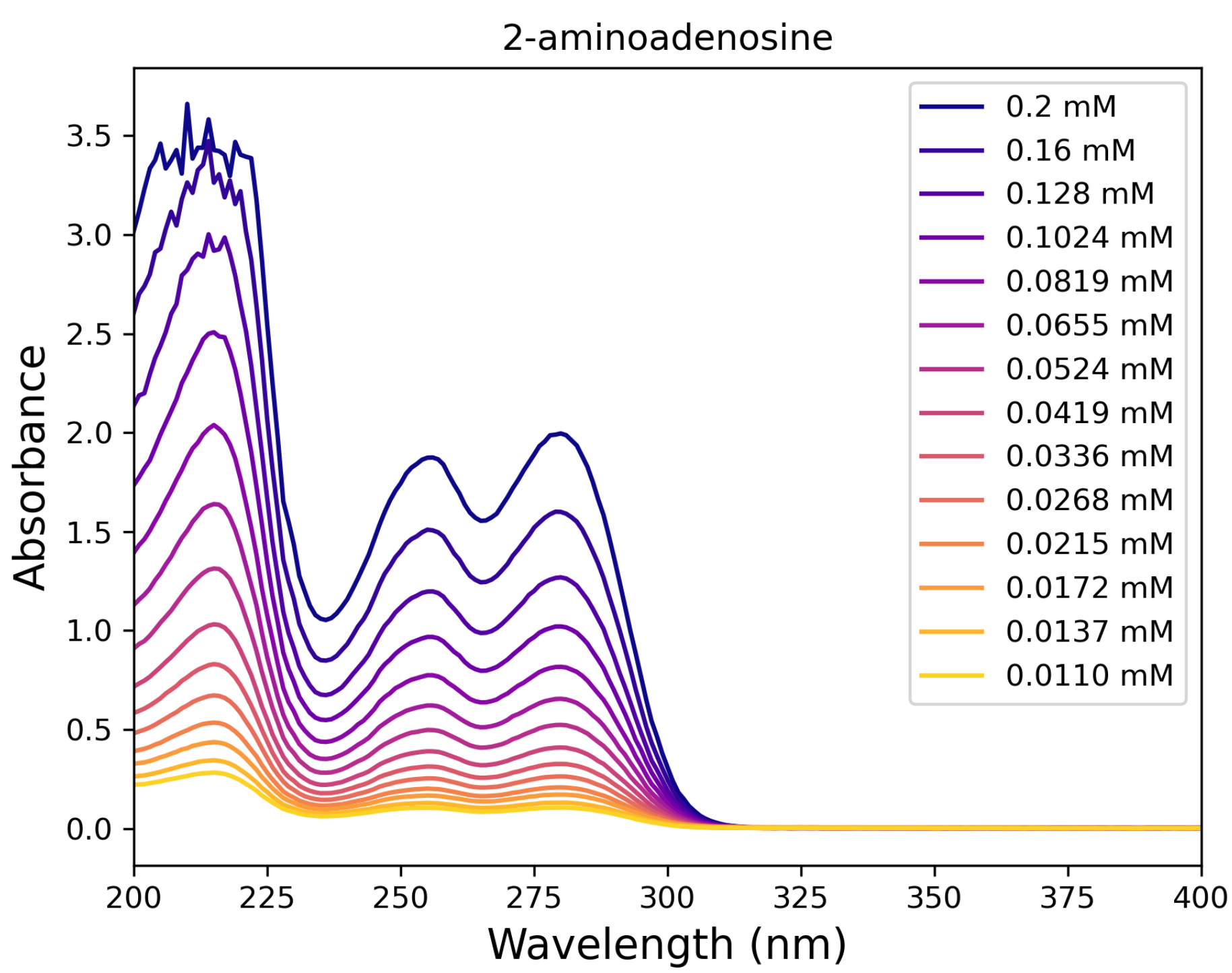

Figure S55: Absorbance spectra of a serial dilution of 2-aminoadenosine.

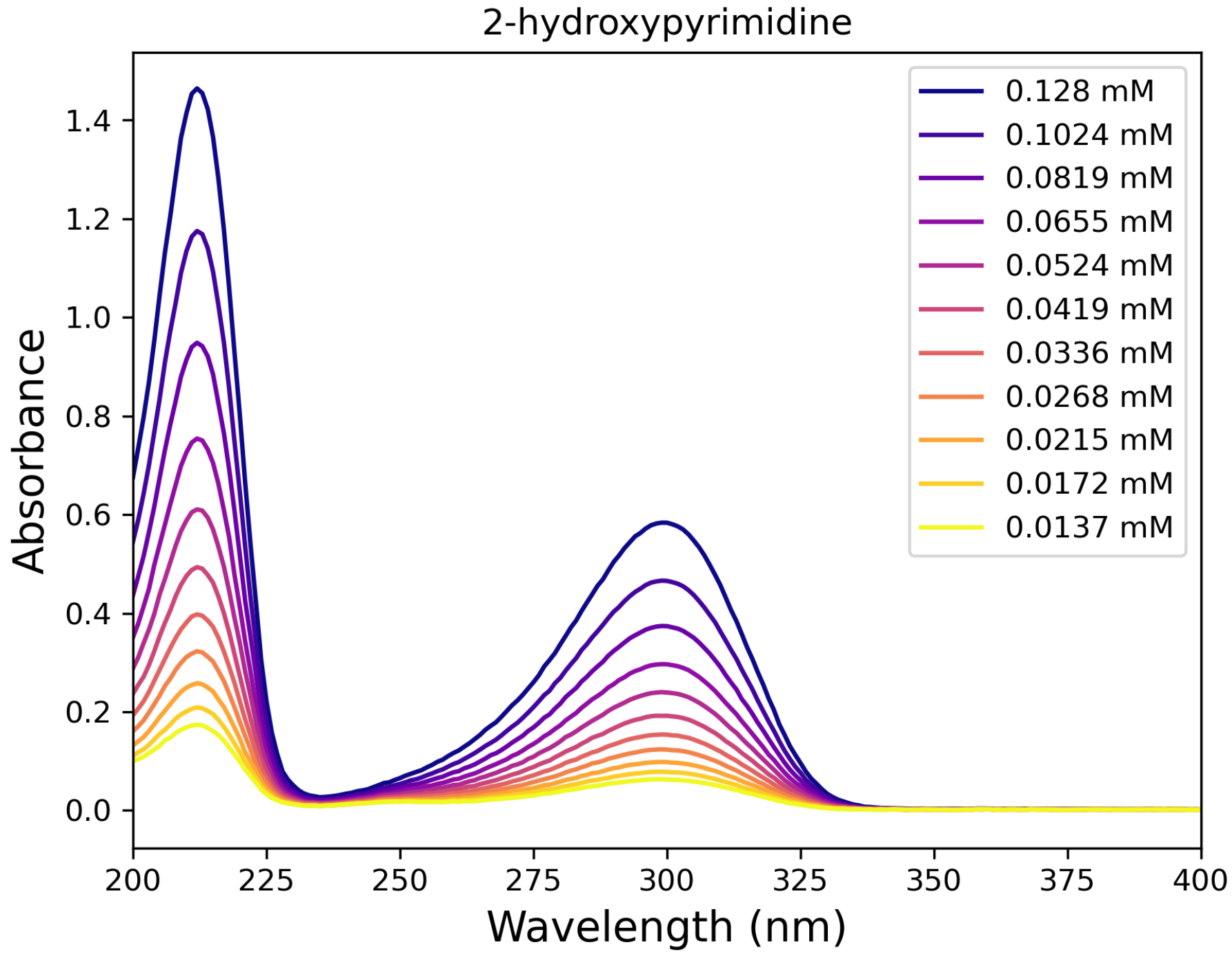

Figure S56: Absorbance spectra of a serial dilution of 2-hydroxypyrimidine (also known as 2-pyrimidinone).

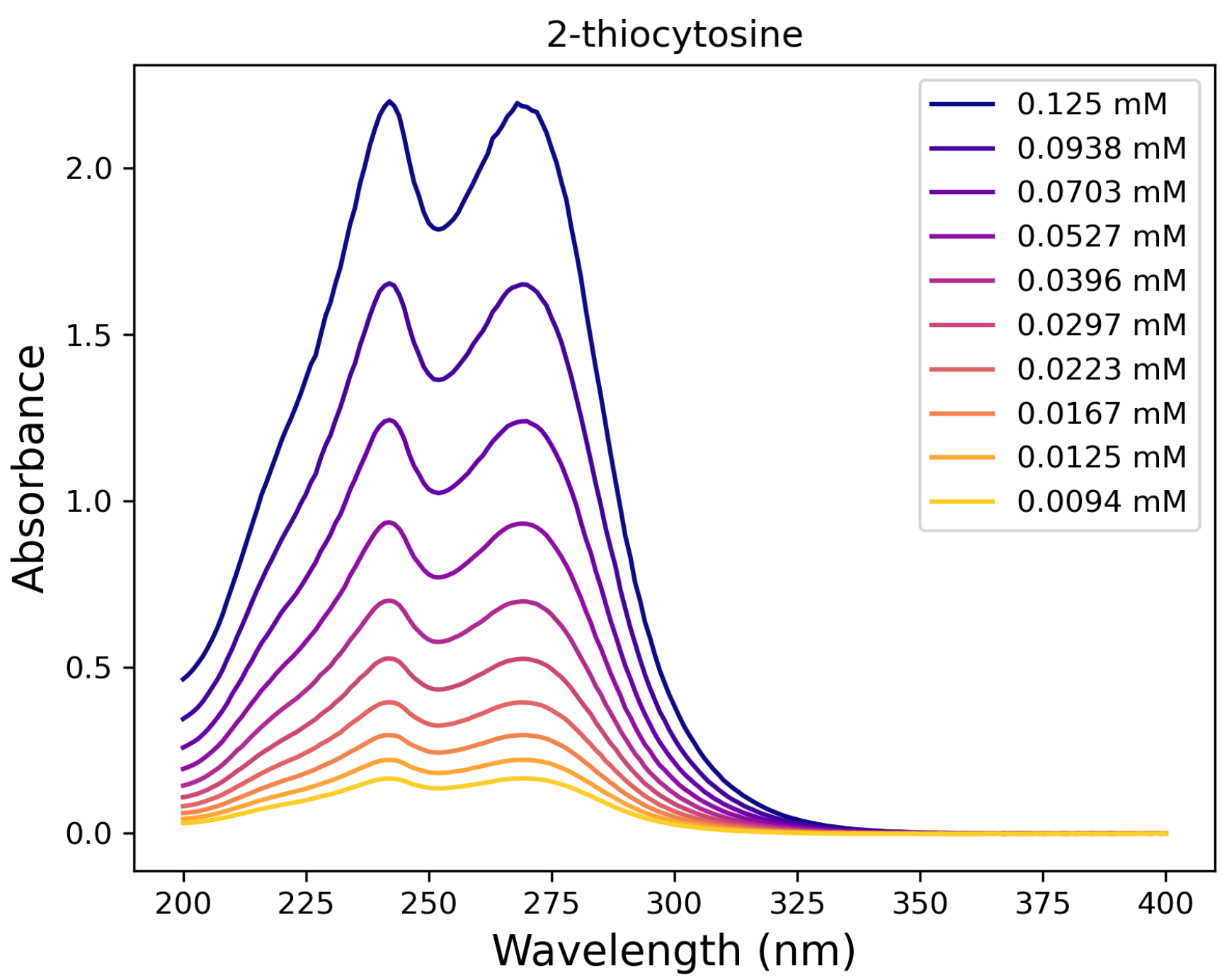

Figure S57: Absorbance spectra of a serial dilution of 2-thiocytosine.

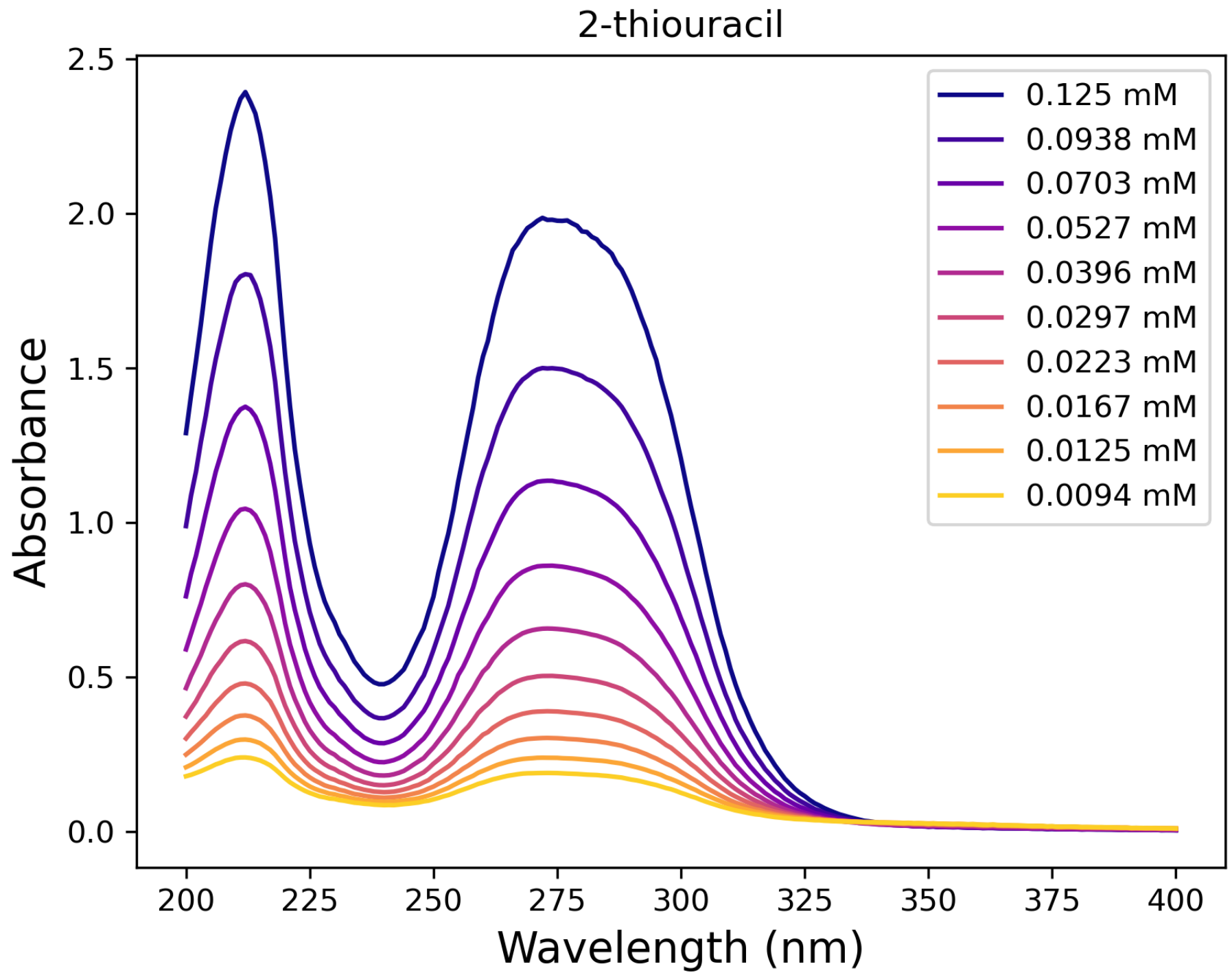

Figure S58: Absorbance spectra of a serial dilution of 2-thiouracil.

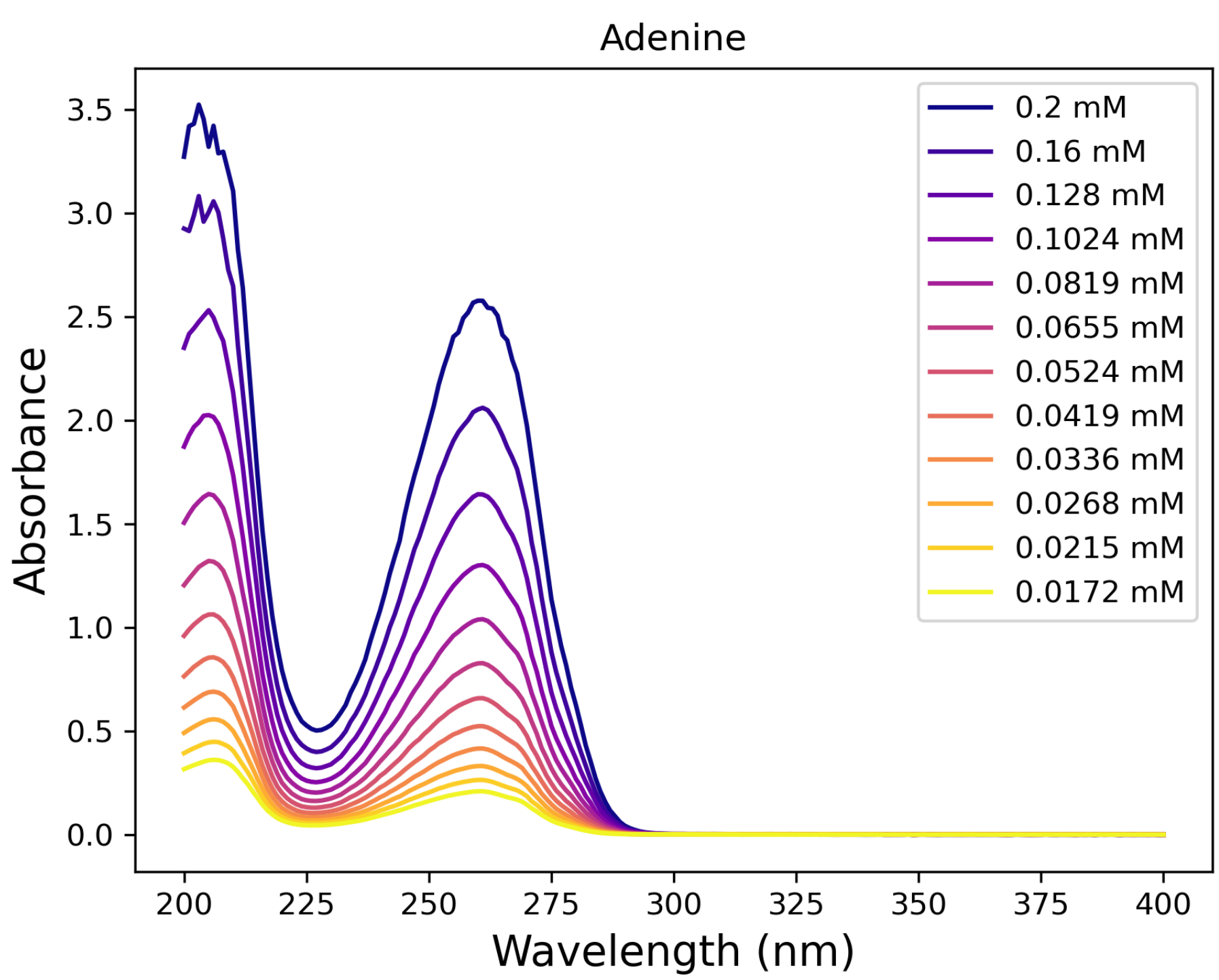

Figure S59: Absorbance spectra of a serial dilution of adenine.

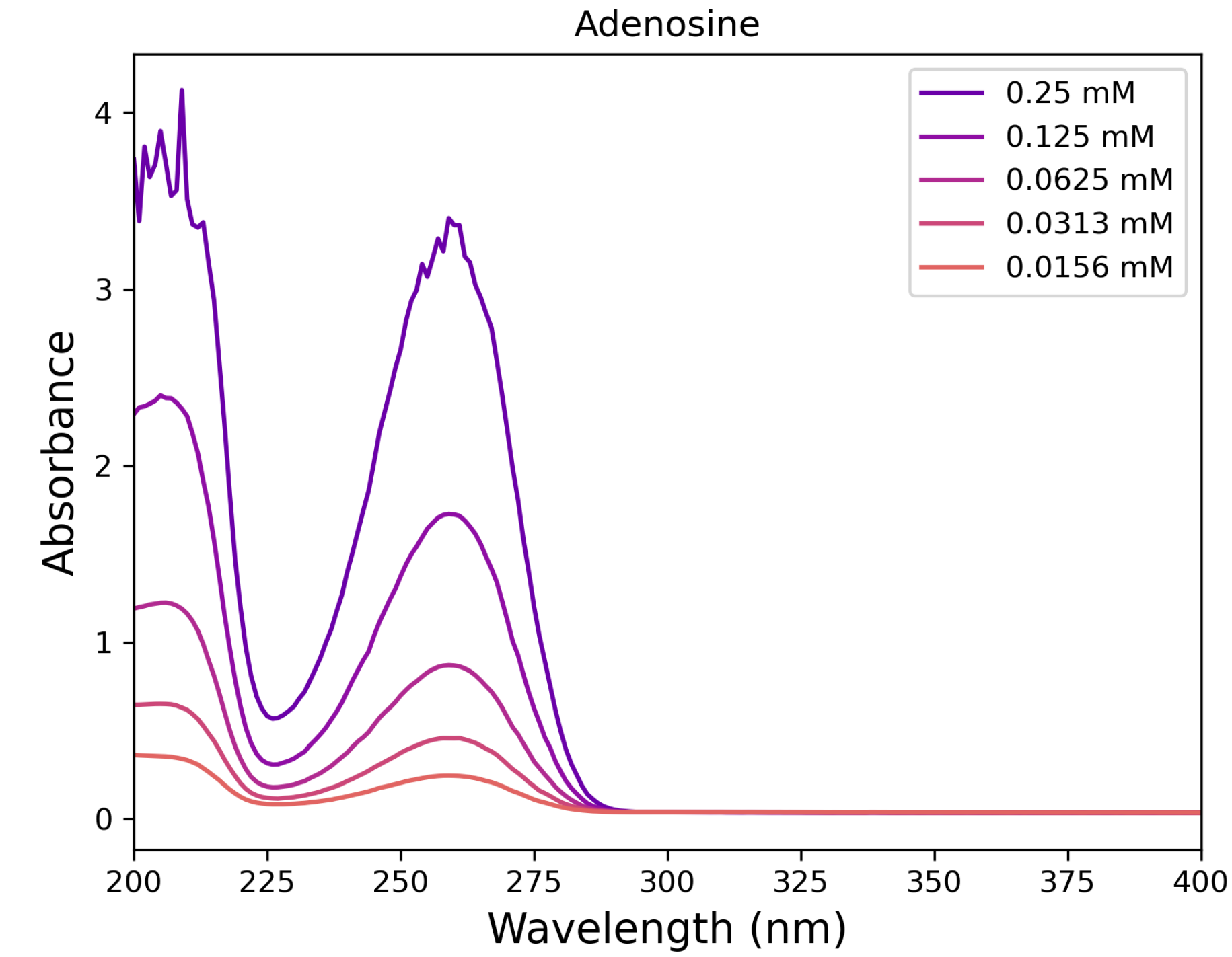

Figure S60: Absorbance spectra of a serial dilution of adenosine.

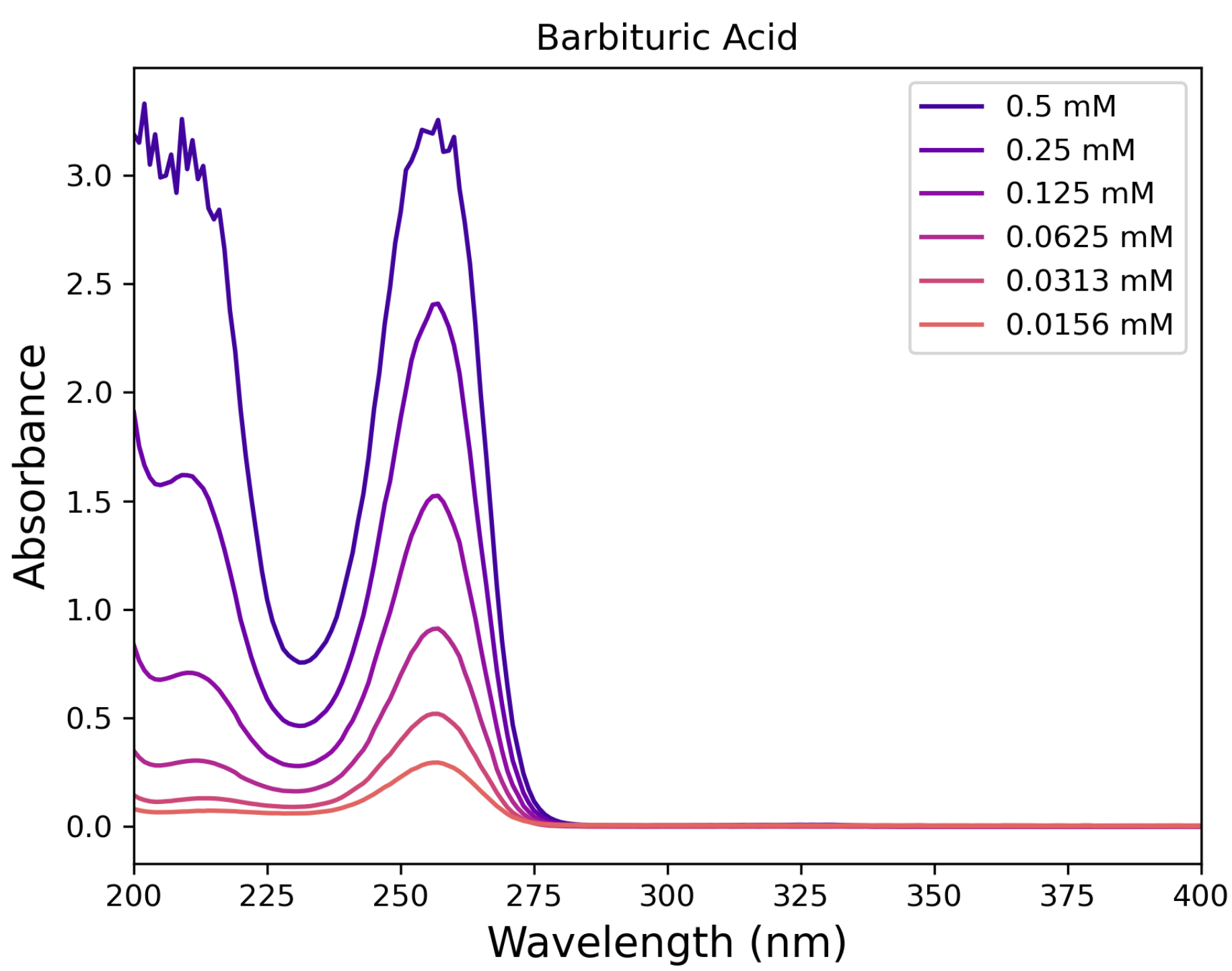

Figure S61: Absorbance spectra of a serial dilution of barbituric acid.

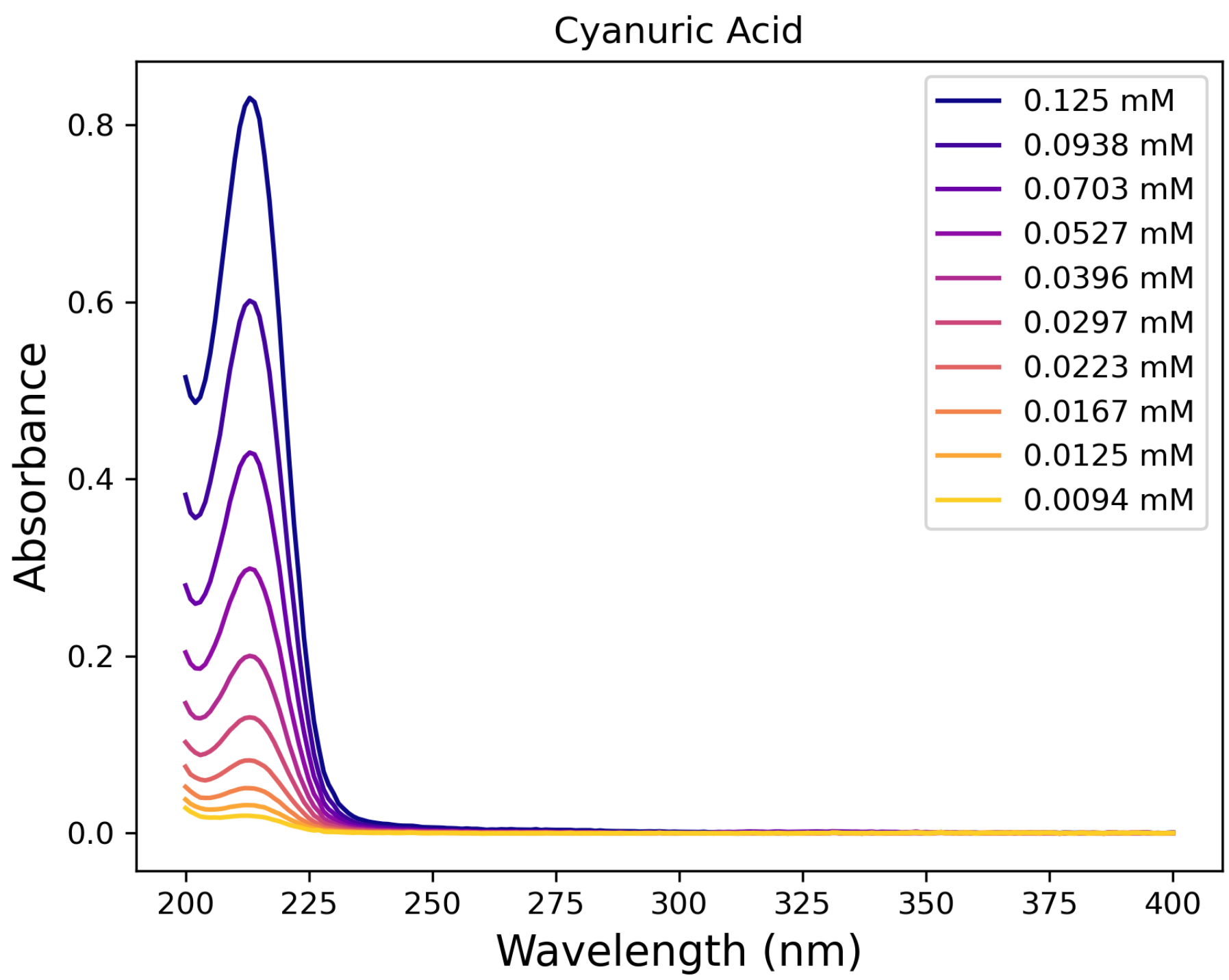

Figure S62: Absorbance spectra of a serial dilution of cyanuric acid (pH adjusted to 7.30).

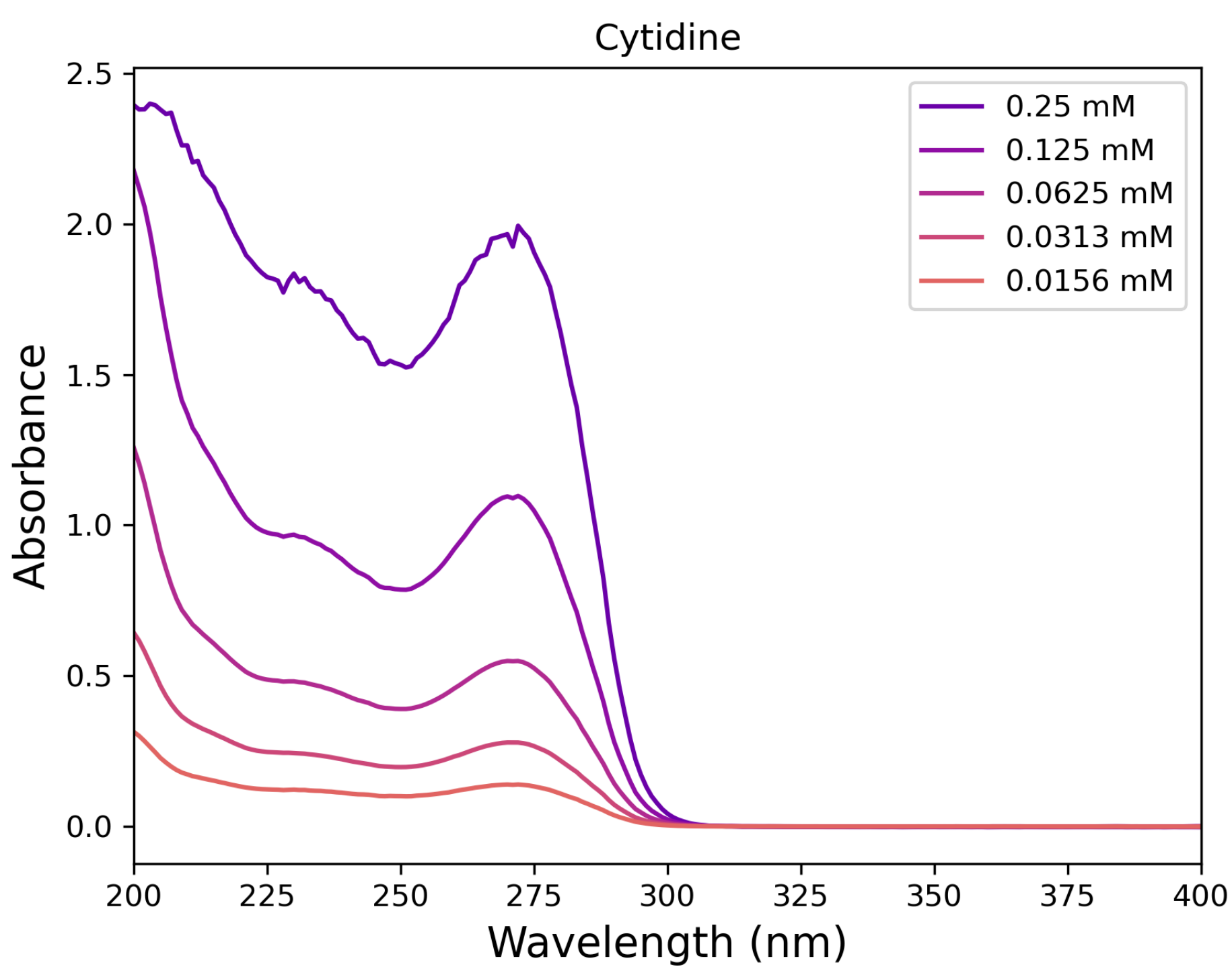

Figure S63: Absorbance spectra of a serial dilution of cytidine.

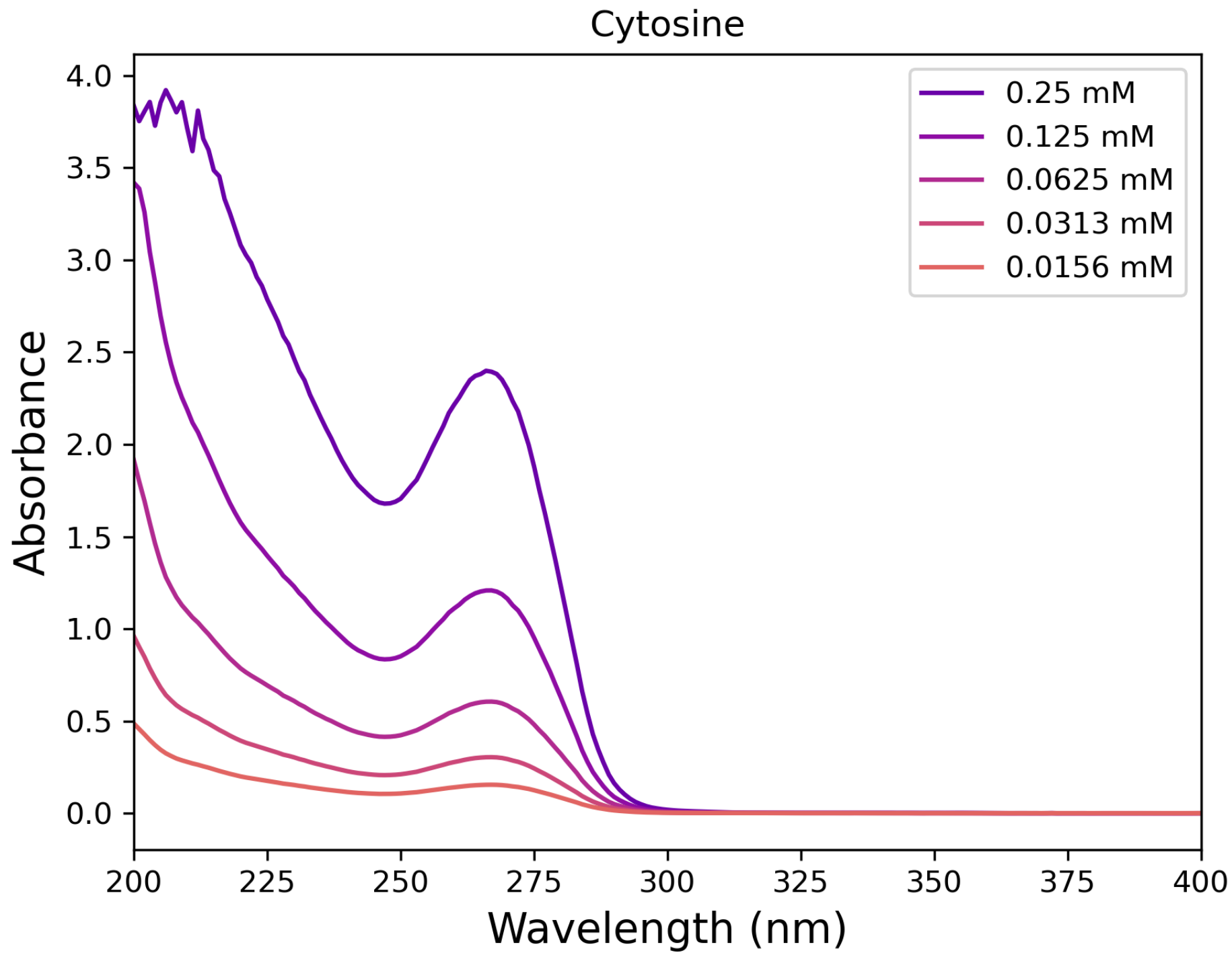

Figure S64: Absorbance spectra of a serial dilution of cytosine.

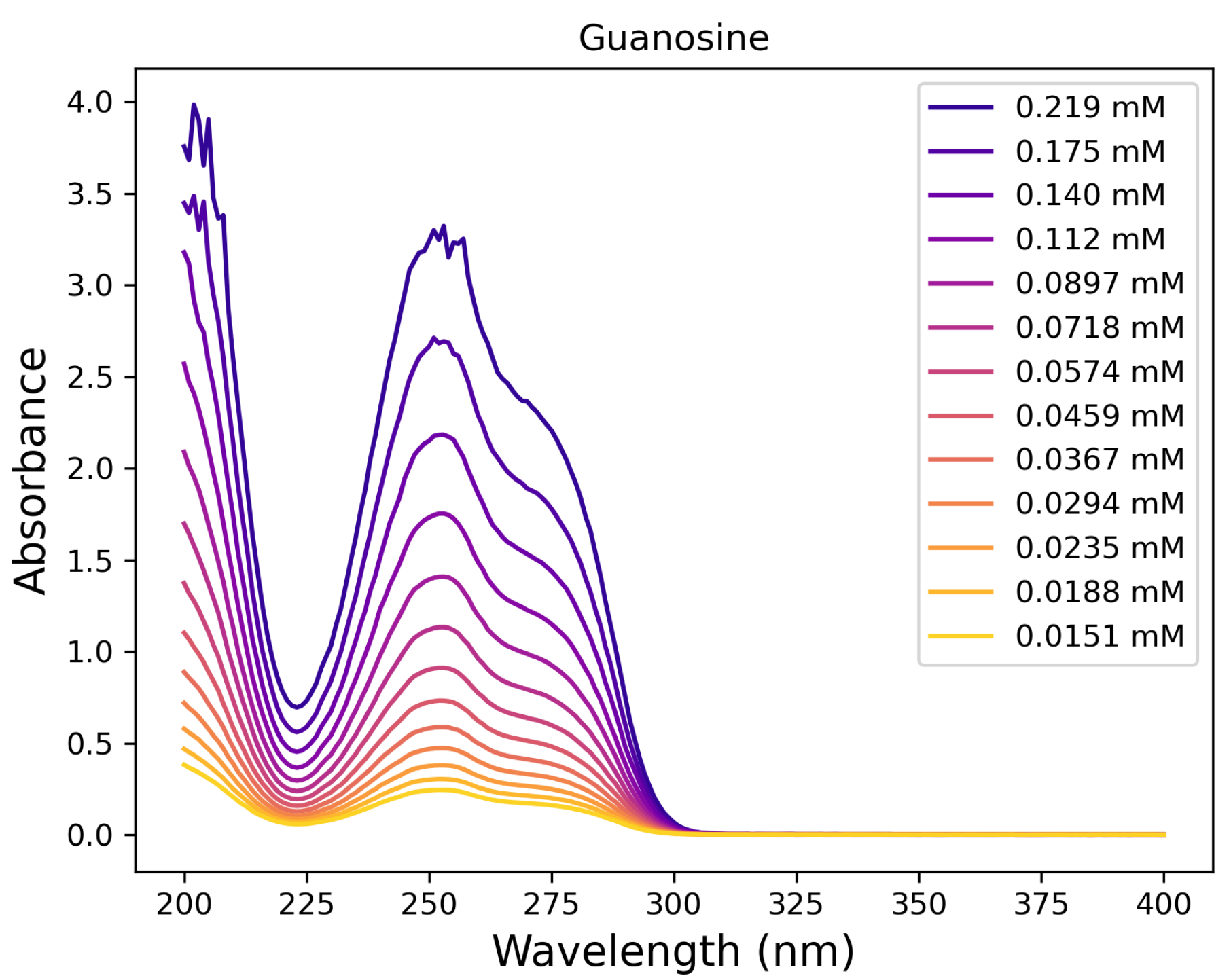

Figure S65: Absorbance spectra of a serial dilution of guanosine.

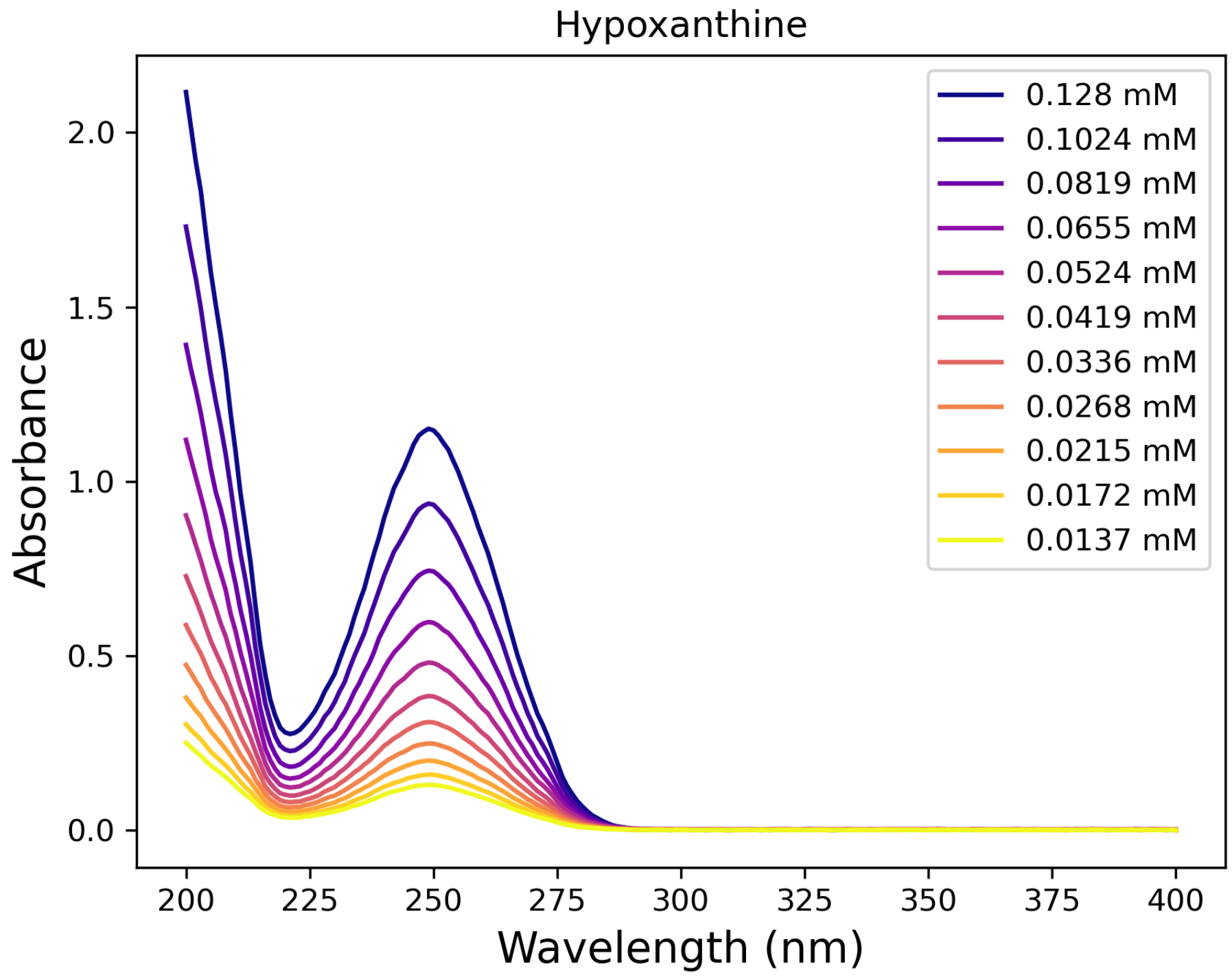

Figure S66: Absorbance spectra of a serial dilution of hypoxanthine.

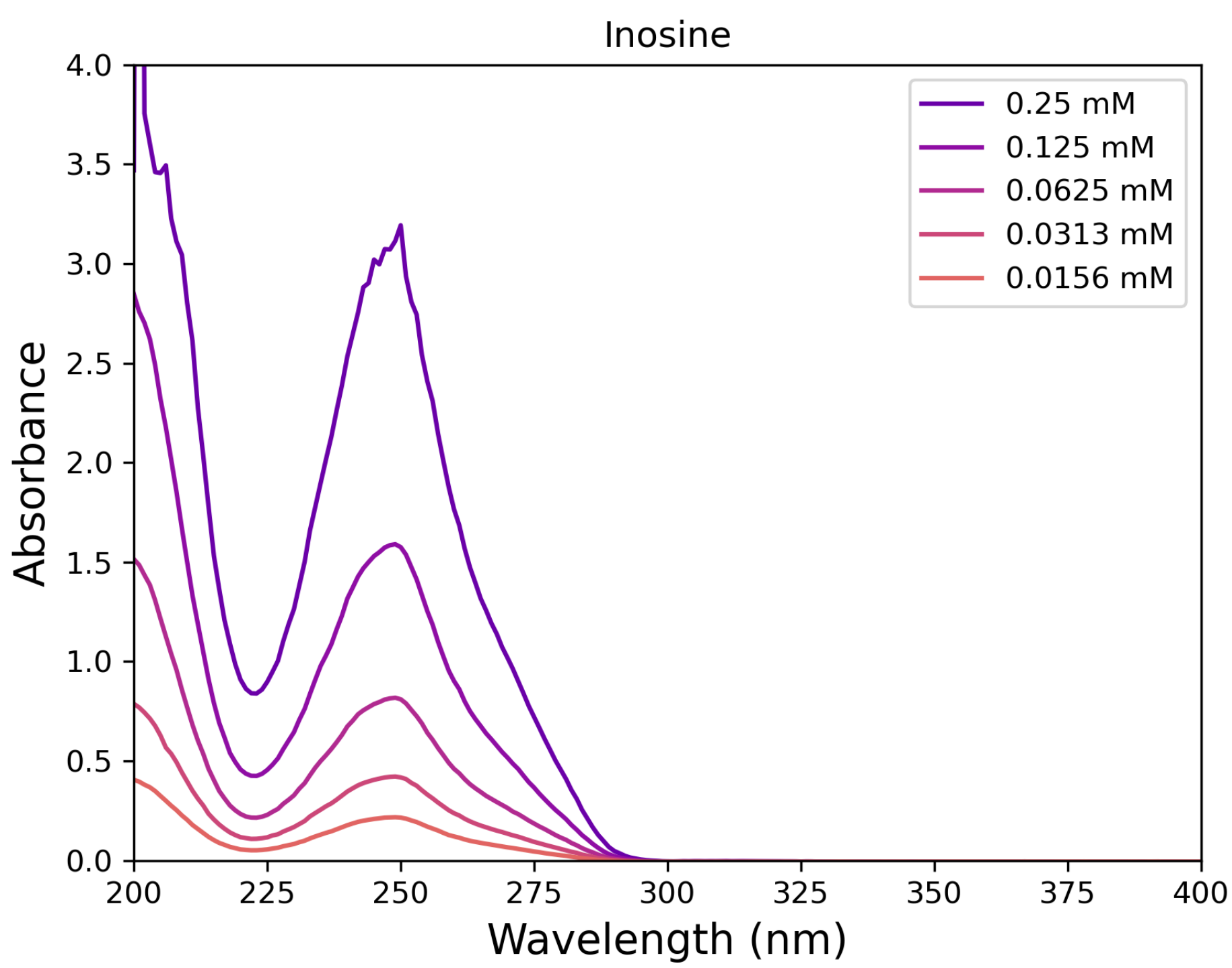

Figure S67: Absorbance spectra of a serial dilution of inosine.

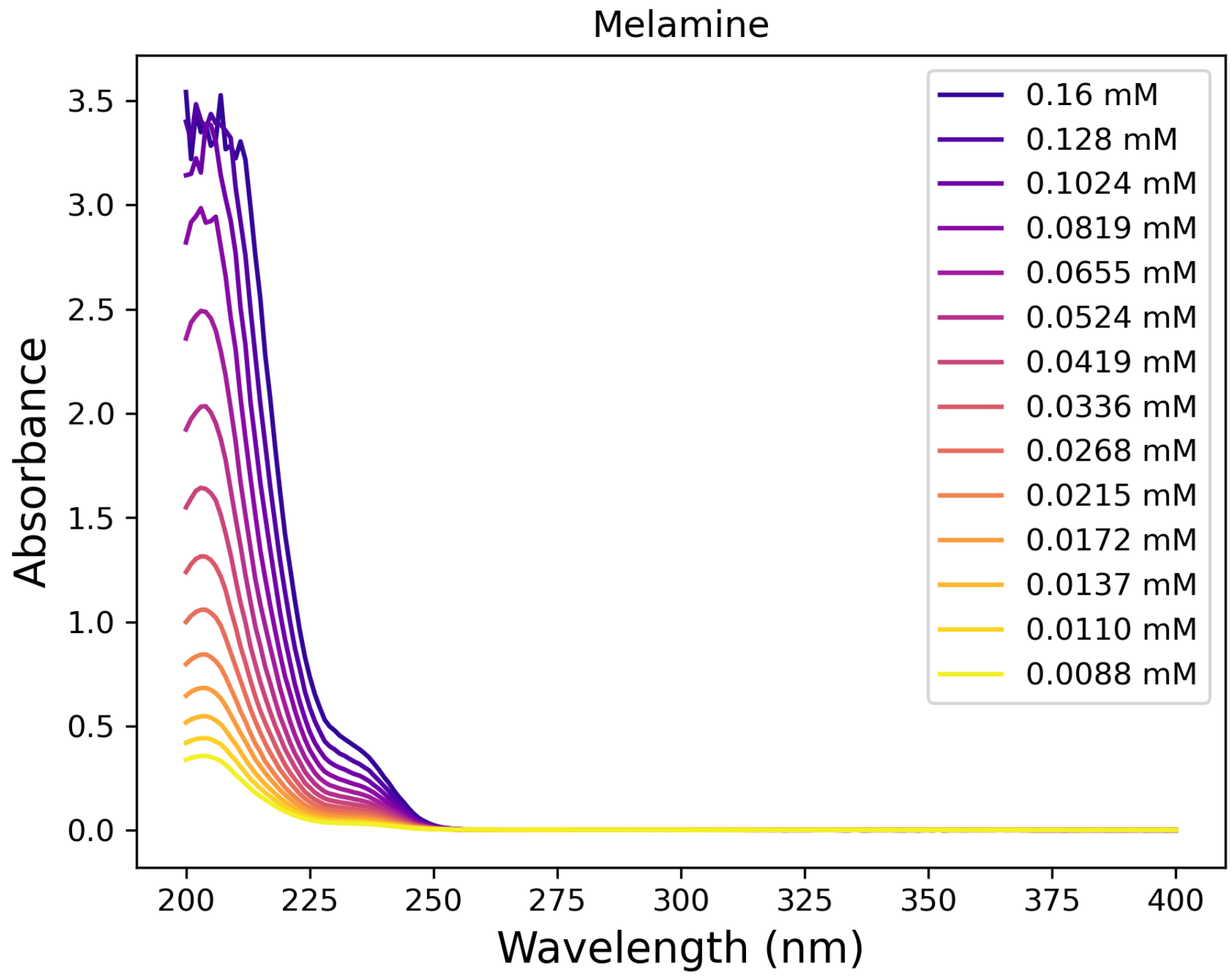

Figure S68: Absorbance spectra of a serial dilution of melamine.

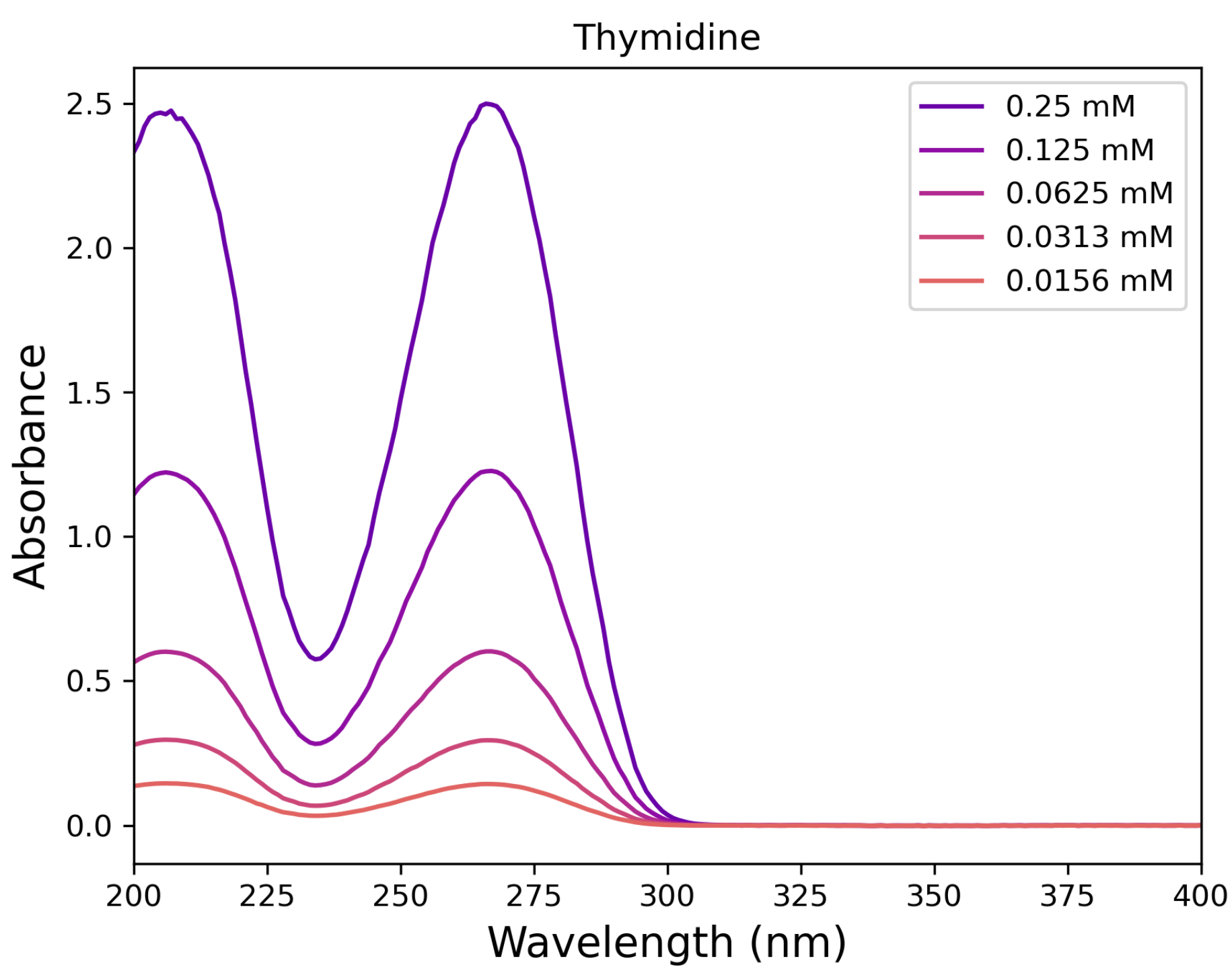

Figure S69: Absorbance spectra of a serial dilution of thymidine.

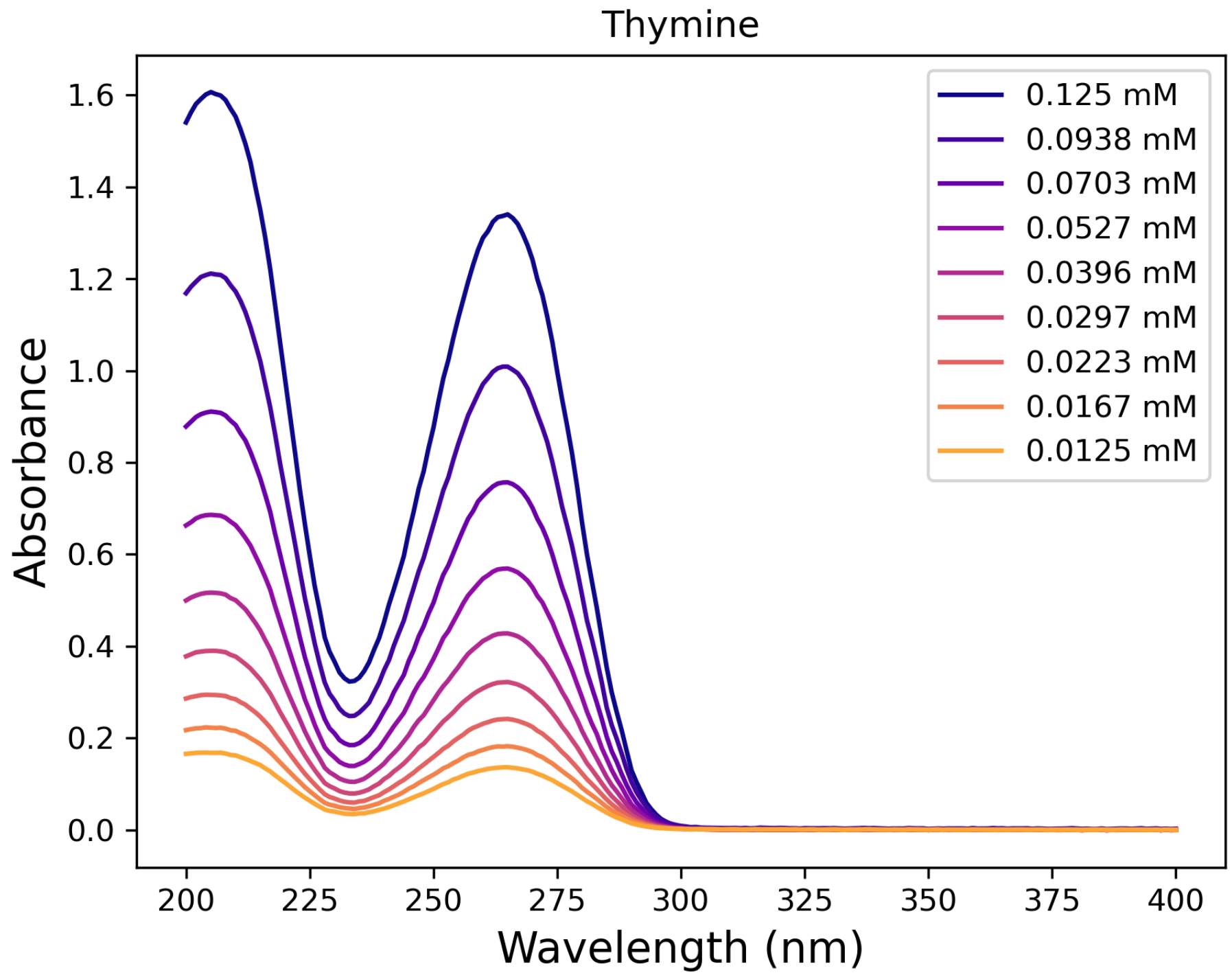

Figure S70: Absorbance spectra of a serial dilution of thymine.

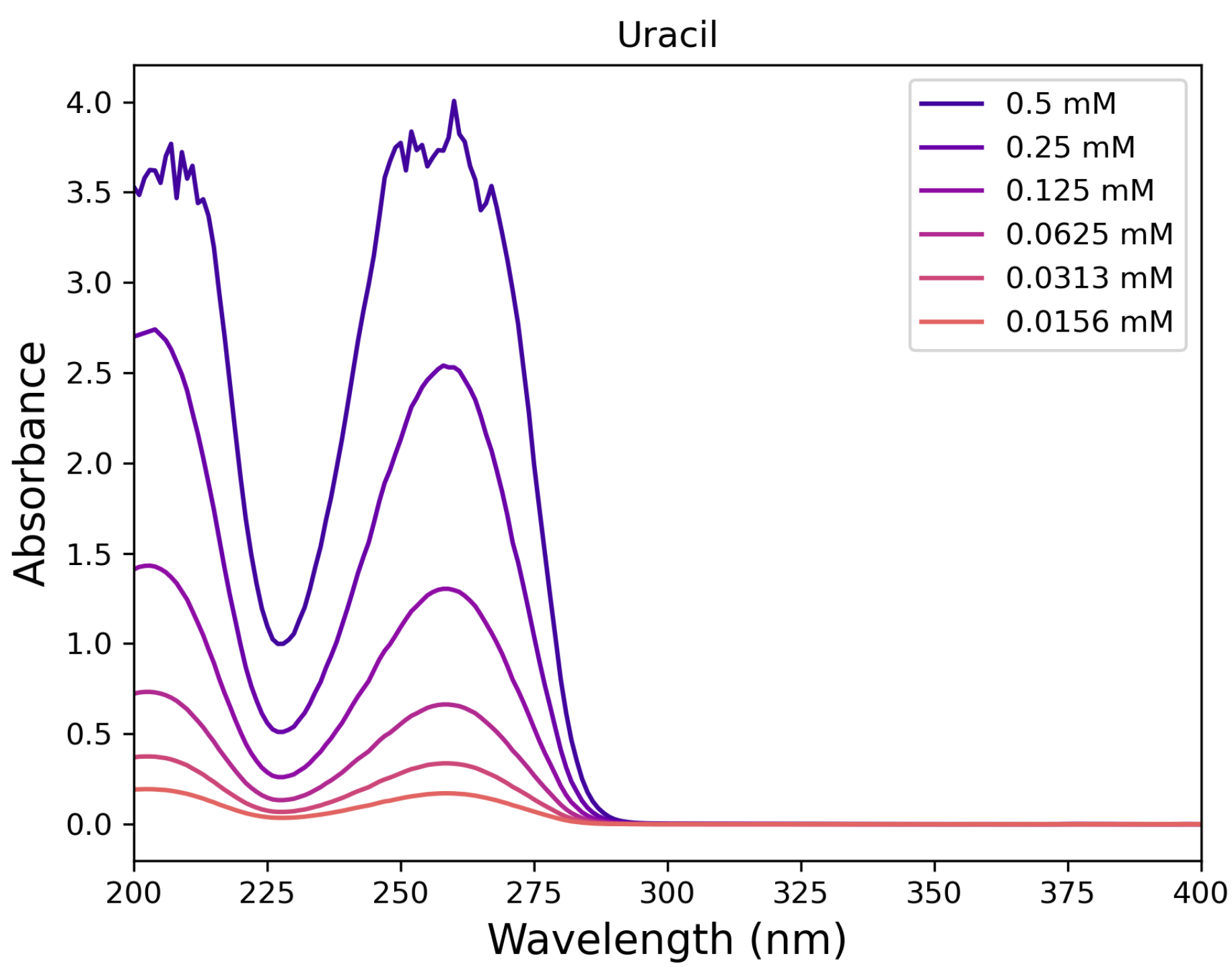

Figure S71: Absorbance spectra of a serial dilution of uracil.

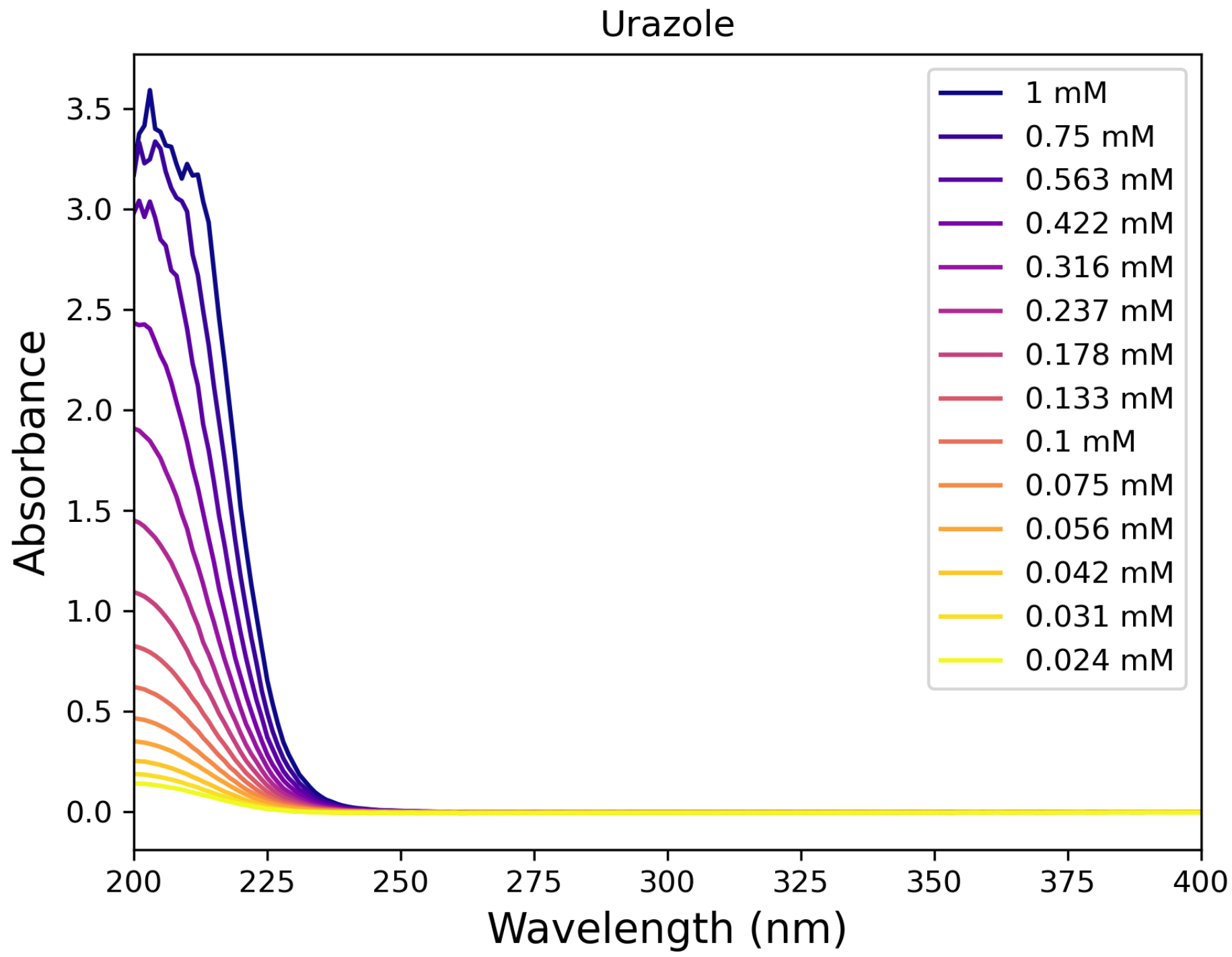

Figure S72: Absorbance spectra of a serial dilution of urazole.

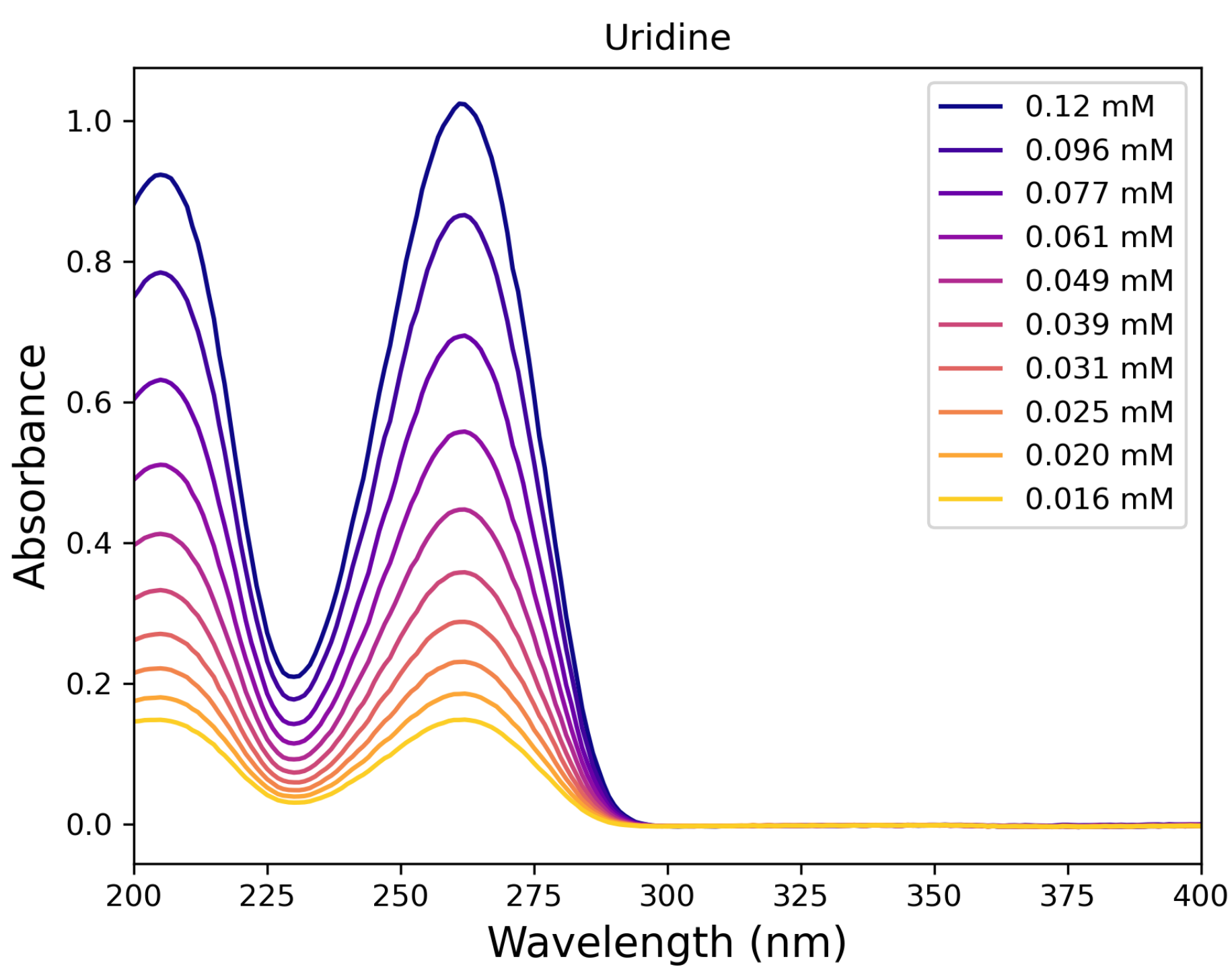

Figure S73: Absorbance spectra of a serial dilution of uridine.

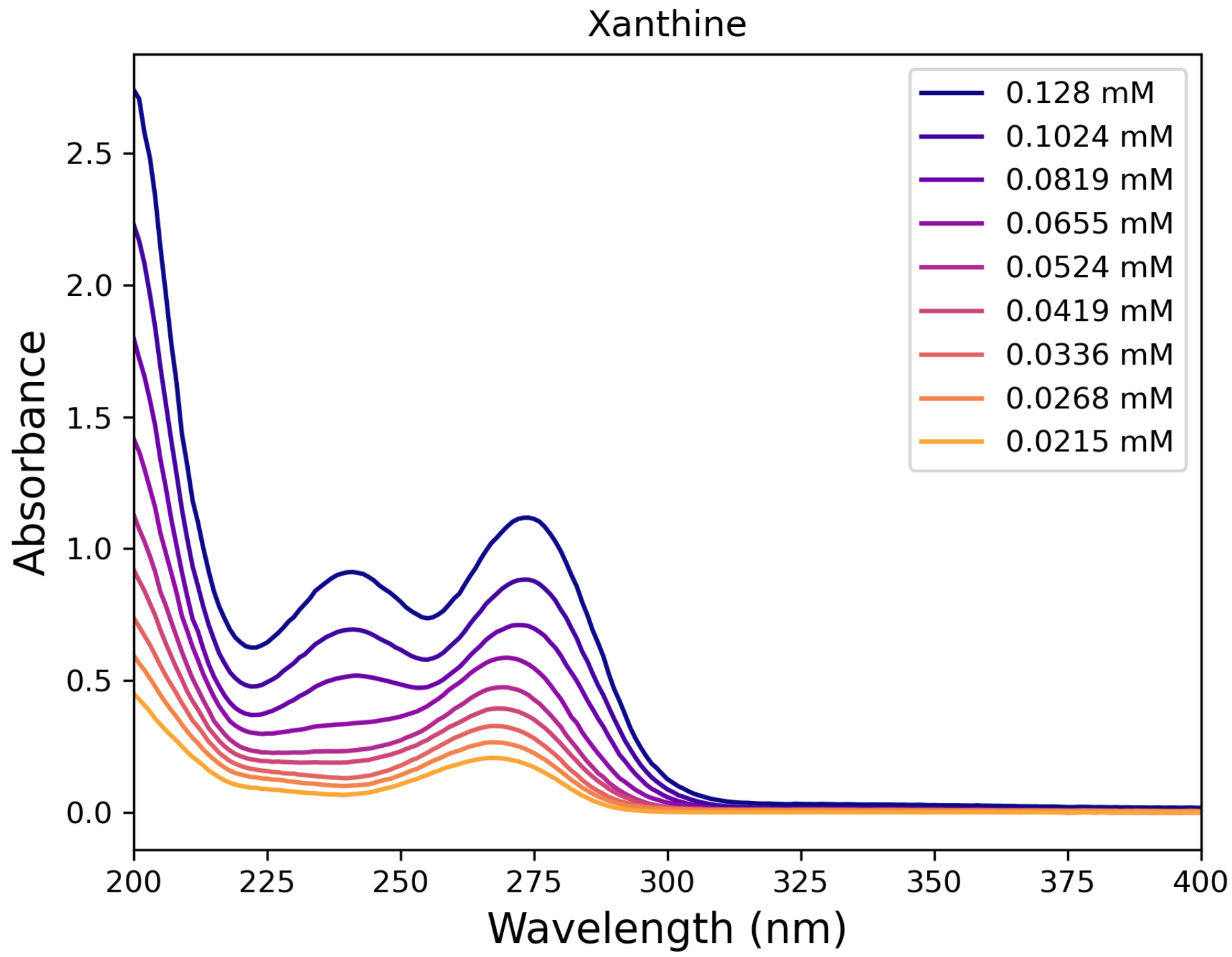

Figure S74: Absorbance spectra of a serial dilution of xanthine.

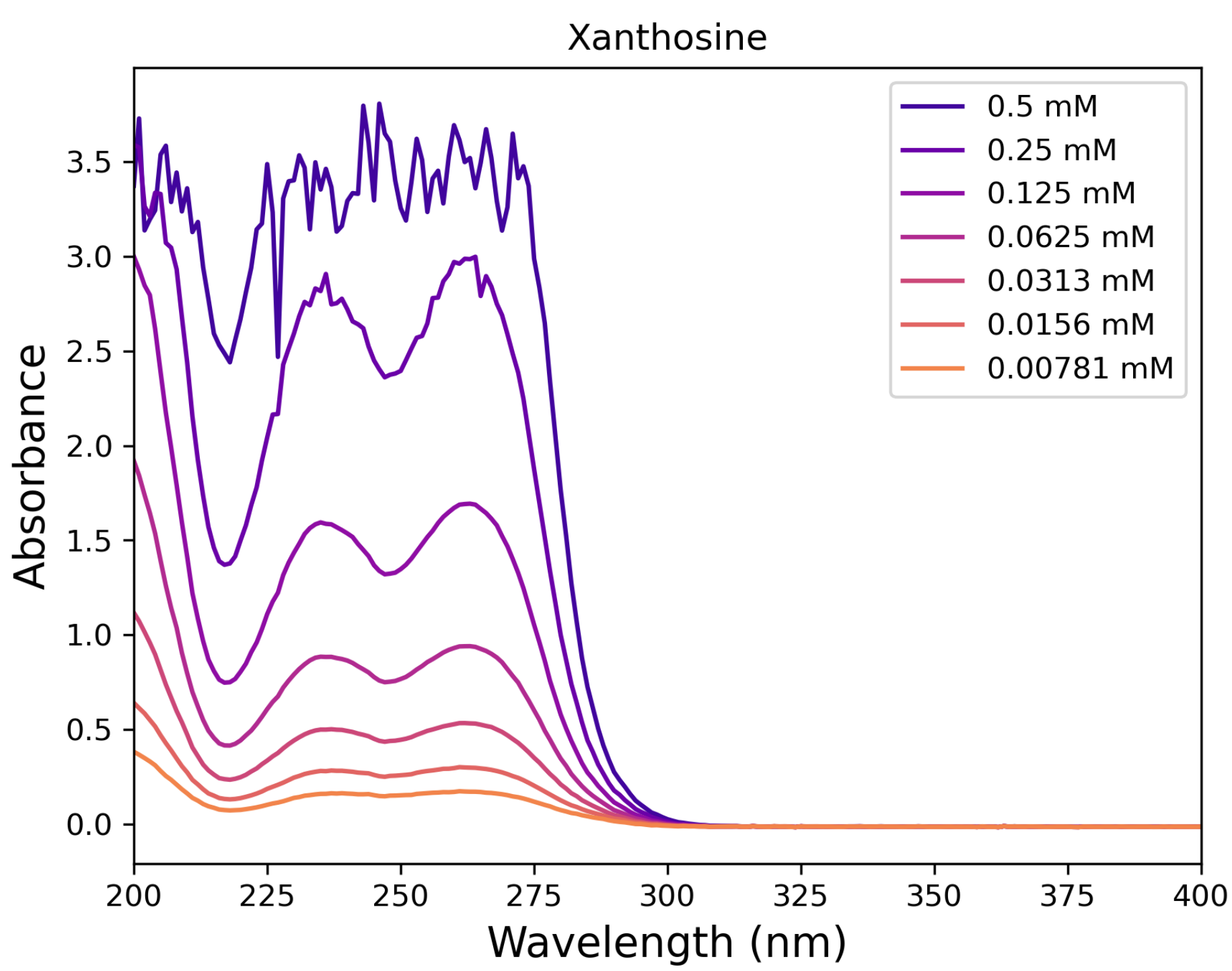

Figure S75: Absorbance spectra of a serial dilution of xanthosine.

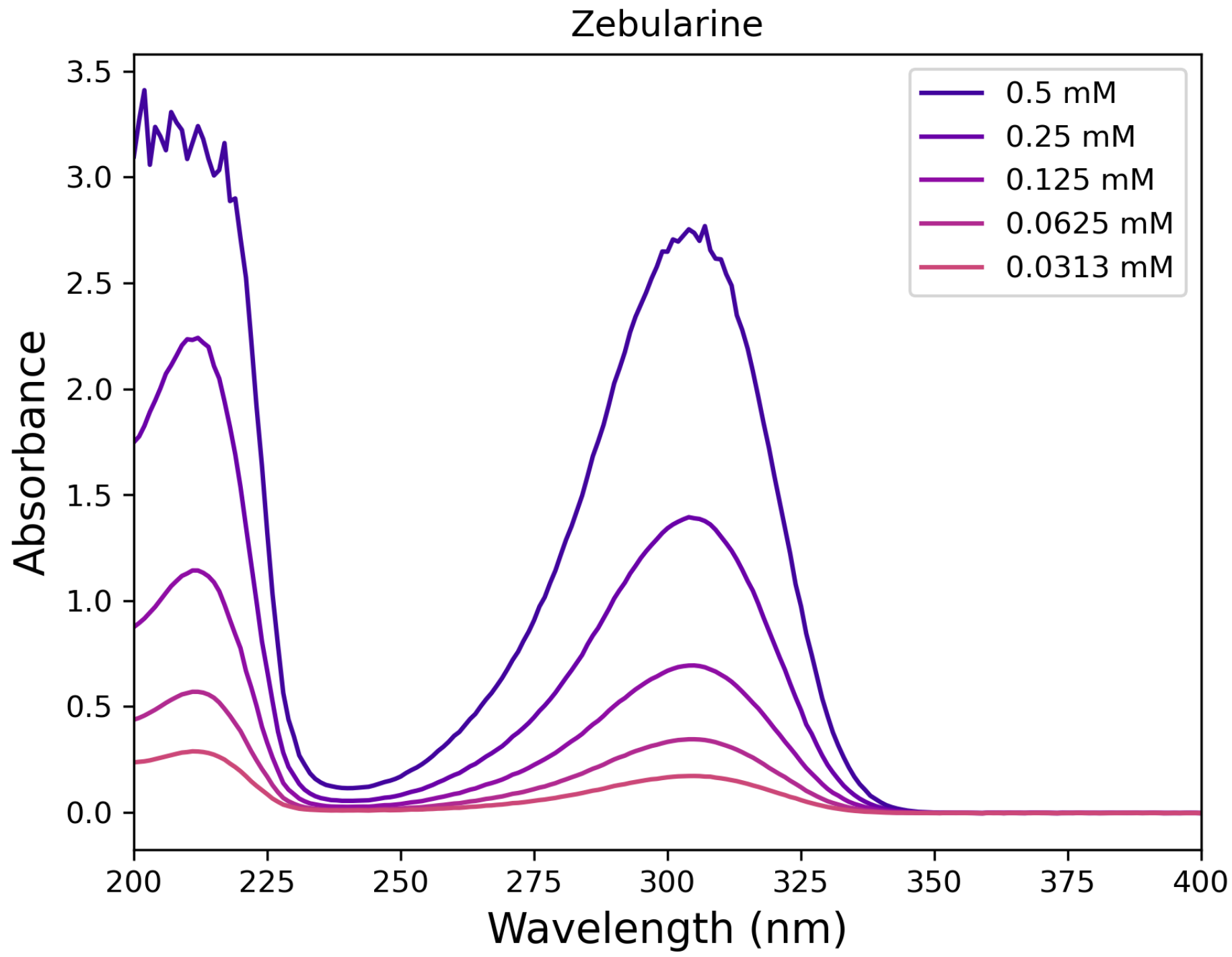

Figure S76: Absorbance spectra of a serial dilution of zebularine.

XI. References

1. Rabani, J., Mamane, H., Pousty, D., Bolton, J. R. (2021) Practical Chemical Actinometry - A Review. *Photochem Photobiol*, 97, 873-902.

2. Roca, M., Zahardis, J., Bone, J., El-Maazawi, M., Grassian, V. H. (2008) 310 nm Irradiation of atmospherically relevant concentrated aqueous nitrate solutions: nitrite production and quantum yields. *J Phys Chem A,* 112, 13275-13281.

3. Pousty, D., Mamane, H., Cohen-Yaniv, V., Bolton, J. R. (2022) Ultraviolet actinometry - Determination of the incident photon flux and quantum yields for photochemical systems using low-pressure and ultraviolet light-emitting diode light sources. *Journal of Environmental Chemical Engineering*, 10, 107947.

4. Rugheimer, S., Segura, A., Kaltenegger, L., Sasselov, D. (2015) UV surface environment of Earth-like planets orbiting FGKM stars through geological evolution. *Astrophys J*, 806, 137.

5. Ranjan, S. and Sasselov, D. D. (2016) Influence of the UV environment on the synthesis of prebiotic molecules. *Astrobiology*, 16, 68-88.

6. Ranjan, S. and Sasselov, D. D. (2017) Constraints on the early terrestrial surface UV environment relevant to prebiotic chemistry. *Astrobiology*, 17, 169-204.

7. Todd, Z. R., Szabla, R., Szostak, J. W., Sasselov, D. D. (2019) UV photostability of three 2-aminoazoles with key roles in prebiotic chemistry on the early Earth. *Chem Commun*, 55, 10388-10391.

8. Todd, Z. R., Lozano, G. G., Kufner, C. L., Sasselov, D. D., Catling, D. C. (2022) Ferrocyanide survival under near ultraviolet (300-400 nm) irradiation on early Earth. *Geochim Cosmochim Acta*, 335, 1-10.

9. Todd, Z. R., Lozano, G. G., Kufner, C. L., Ranjan, S., Catling, D. C., Sasselov, D. D. (2024) UV transmission in prebiotic environments on early Earth. *Astrobiology*, 24, 559-569.
